# Comorbidities and complications of COVID-19 associated with disease severity, progression, and mortality in China with centralized isolation and hospitalization: A systematic review and meta-analysis

**DOI:** 10.3389/fpubh.2022.923485

**Published:** 2022-08-16

**Authors:** Zhe Chen, Yingying Peng, Xiaolei Wu, Bo Pang, Fengwen Yang, Wenke Zheng, Chunxiang Liu, Junhua Zhang

**Affiliations:** ^1^Evidence-Based Medicine Center, Tianjin University of Traditional Chinese Medicine, Tianjin, China; ^2^First Teaching Hospital of Tianjin University of Traditional Chinese Medicine, Tianjin, China; ^3^National Clinical Research Center for Chinese Medicine Acupuncture and Moxibustion, Tianjin, China

**Keywords:** comorbidities, complications, COVID-19, centralized isolation and hospitalization, systematic review

## Abstract

**Background:**

Coronavirus disease 2019 (COVID-19) causes life-threatening with the high-fatality rates and spreads with high-infectious disease worldwide. We aimed to systematically review the comorbidities and complications of COVID-19 that are associated with various disease severity, progression, and mortality in China, to provide contemporary and reliable estimates in settings with centralized isolation and hospitalization.

**Methods:**

In this systematic review and meta-analysis, we searched four main English language databases, and four main Chinese language databases for observational studies published from inception to January 2022, to identify all the related comorbidities and complications of COVID-19, in the China region with centralized isolation and hospitalization, with disease severity, progression, and mortality. Literature search, data extraction, and quality assessment were independently conducted by two reviewers. We used the generalized linear mixed model to estimate pooled effect sizes for any comorbidities and complications, and subgroup in gender ratio was done to further address the potential heterogeneity.

**Results:**

Overall, 187 studies describing 77,013 patients, namely, 54 different comorbidities and 46 various complications of COVID-19, were identified who met our inclusion criteria. The most prevalent comorbidities were hypertension [20.37% 95% CI (15.28–26.63), 19.29% (16.17–22.85), 34.72% (31.48–38.10), and 43.94% (38.94–49.06)] and diabetes [7.84% (5.78–10.54), 8.59% (7.25–10.16), 17.99% (16.29–19.84), and 22.68% (19.93–25.69)] in mild, moderate, severe, and critical cases. The most prevalent complications were liver injury [10.00% (1.39–46.72), 23.04% (14.20–35.13), and 43.48% (39.88–47.15)] in mild, moderate, and severe cases, and acute respiratory distress syndrome [ARDS; 94.17% (20.78–99.90)] and respiratory failure [90.69% (28.08–99.59)] in critical cases. Renal insufficiency [odds ratio (OR) 17.43 (6.69–45.43)] in comorbidities and respiratory failure [OR 105.12 (49.48–223.33)] in complications were strongly associated in severe/critical than in mild/moderate cases. The highest estimated risk in intensive care unit (ICU) admission, progression, and mortality was an autoimmune disease, nervous system disease, and stroke in comorbidities, shock, and ARDS in complications.

**Conclusion:**

Comorbidities and complications in inpatients with COVID-19 were positively associated with increased risk in severe and critical cases, ICU admission, exacerbation, and death during centralized isolation and hospitalization. Prompt identification of comorbidities and complications in inpatients with COVID-19 can enhance the prevention of disease progression and death and improve the precision of risk predictions.

## Introduction

The coronavirus disease 2019 (COVID-19) pandemic poses a formidable challenge to socioeconomic development and population health (1, 2). COVID-19 is associated with high global morbidity and in-hospital fatality rates, continues to spread due to its highly infectious nature, and is prevalent worldwide (3–5). In 2020–2022, COVID-19 increasingly emerged as a major cause of mortality worldwide, had a catastrophic effect on public health, and was largely responsible for comorbidities and complications in infected individuals (3, 6, 7).

More recent COVID-19 data indicate that severe disease conditions, disease progression, and mortality risk substantially depend on the underlying comorbidities and specific complications, which cause higher risk and poor prognosis (8–10). Existing comorbidities and short-term complications can cause more severe outcomes, such as exacerbations and death, compared with an initial infection by severe acute respiratory distress syndrome coronavirus 2 (SARS-CoV-2) (7, 11). Identifying these prognosis factors in a clinical setting is significant in managing inpatients with COVID-19 and also contributes to future clinical decisions to resolve health threats in healthcare systems (12–14). However, comprehensive studies summarizing the ongoing relevance regarding comorbidities and complications in patients with COVID-19 are sparse, particularly in settings with the centralized isolation and active treatment.

Epidemic control in risk populations, including positive cases and in cases of proximity contact, can considerably alleviate SARS-CoV-2 transmission (15, 16). As the first wave of SARS-CoV-2 was detected in China, the Chinese government imposed strict lockdown measures to control outbreaks and setup quarantine and hospitalization facilities on priority for patients afflicted with COVID-19 (17, 18). The low prevalence and repositivity rate in patients infected with COVID-19 in China indicate that strict lockdown measures and centralized isolation and treatment could control the COVID-19 epidemic over a short time (19, 20).

Previous studies have not fully considered and rigorously evaluated COVID-19-related comorbidities and complications in different disease states, and most studies with small sample sizes have neglected important clinical risk factors and estimated the results in an overly simplistic to the inconclusive manner (21–23). The fundamental aspects of numerous comorbidities and complications associated with COVID-19 have not been defined. To provide an appropriate public health response, in this systematic review and meta-analysis, we aimed to identify and assess in-hospital data on comorbidities and complications of COVID-19 that are associated with various levels of disease severity, progression, and mortality in China during centralized isolation and hospitalization.

## Methods

This systematic review and meta-analysis were conducted in accordance with the Preferred Reporting Items for Systematic Reviews and Meta-Analyses (PRISMA) guidelines for its analyses and synthesis (24). The detailed plan of our protocol is published and registered with the International Prospective Register of Systematic Reviews (PROSPERO) (registration ID: CRD42022311096). Detailed information on protocol amendments is given in Supplementary File 1.

### Eligibility criteria

Observational studies that detailed all the related comorbidities and complications of COVID-19 in the general Chinese population (individuals aged >15 years), and studies reporting various disease severity, progression, and mortality, in designated hospitals with centralized isolation and treatment, were included. The reference standards for confirmed SARS-CoV-2 infections were determined by following the WHO interim guidance, the COVID-19 diagnosis and treatment guidelines of the National Health Commission in China, or others. Studies having a sample size of more than 100 participants were included. All the comorbidities and complications of COVID-19 were determined based on author-defined criteria reported in the original articles. Studies that included pregnant women, maternal cases, newborns, and children; those conducted in a certain subpopulation without comparison with other subgroups; those that included the general population vaccinated against COVID-19; studies that were missing information with respect to data sources or clinical prognostic factor in comorbidities or complications; and studies that identified disease diagnosis and COVID-19 severity using unclear or inconsistent definitions were excluded.

### Search strategy

In this systematic review and meta-analysis, we conducted a literature search of English language databases (PubMed, Embase, Cochrane Library, and the Web of Science), and Chinese language databases (China National Knowledge Infrastructure, Wanfang Data Knowledge Service Platform, VIP information resource integration service platform databases, and Chinese biomedical literature service system) without language restrictions from inception to January 2022 that reported centralized isolation and hospitalization in China. Key search terms were “COVID-19” and “comorbidities” and “complications.” Detailed strategies are available in Supplementary File 2. Furthermore, reference lists of the included articles and related systematic reviews that met our inclusion criteria on COVID-19 were checked and the gray literature was explored by hand search.

### Data extraction

The titles and abstracts of all the retrieved studies, after the removal of duplicates, were first independently screened by two reviewers. Subsequent steps involved the full-text review of potential studies to assess their eligibility for inclusion. A third reviewer arbitrated discrepancies between the two reviewers and made the final decision that was resolved by consensus and discussion. Extracted data that were included were comorbidities and complications in various disease conditions, including disease severity, progression, and mortality. We also extracted data on authors, year of publication, site and date of investigation, hospital of recruitment, definition and diagnosis of COVID-19, sample size, median age, and the gender proportion of participants.

### Quality assessment

Two reviewers independently assessed the quality of all the included studies using a 9-item tool in the Joanna Briggs Institute (JBI) Prevalence Critical Appraisal Checklist, where each item is scored on a scale of 0–1, with 0 for “No” or “Unclear,” and 1 for “Yes” (25). The score of studies is categorized as high risk of bias (0–5), moderate risk of bias (6, 7), or low risk of bias (8, 9). If disagreements arose, a third independent reviewer resolved them by consensus.

### Data analysis

A generalized linear mixed model (GLMM) with random-effects and fixed-effects models was used to calculate and estimate the overall pool of the single group proportions of comorbidities or complications in mild, moderate, severe, and critical patients, and the total number of COVID-19 inpatients. The proportion was modeled by a logit transformation in GLMM. The between-study variance was estimated using the DerSimonian–Laird estimator with corresponding *I*^2^ and *Q* statistics for quantifying and testing heterogeneity. Because of the clinically heterogeneous nature of the between-study populations, all the results were reported using the random-effects models. *I*^2^ statistic was used to quantify the heterogeneity of between-study findings. Values of <50% represented low heterogeneity, 50%−75% represented medium heterogeneity, and >75% represented high heterogeneity. A two-tailed *p*-value of <0.05 was used as a threshold for the statistical significance in all the analyses.

Subgroup analyses on disease severity for the estimated proportions were based on mild, moderate, severe, and critical cases by clinical symptoms at enrollment for estimated proportions. In this meta-analysis, for all the comparisons and proportions that had more than five included studies, subgroup analysis for gender ratio, especially sex-specific analysis, including female > male (sex ratio > 1) and female < male (sex ratio <1), was performed. The classifications of severe illness for “mild/moderate vs. severe/critical” and “intensive care unit (ICU) vs. non-ICU,” progression in “progressive (aggravation of illness) vs. nonprogressive (nonaggravation of illness),” and mortality in “with comorbidities and complications vs. without comorbidities and complications” were combined and analyzed separately using odds ratio (OR) with 95% CI for binary variables. These parameters were compared using the Mantel–Haenszel pooling method from pairwise meta-analyses. All the statistical analyses were conducted using *R* statistical software (version 4.0.5) with meta-package.

### Patient and public involvement

This study is a systematic review, based on the published data; no patients were involved in conducting the study.

## Results

### Search results and characteristics of included studies

**Figure 1:**
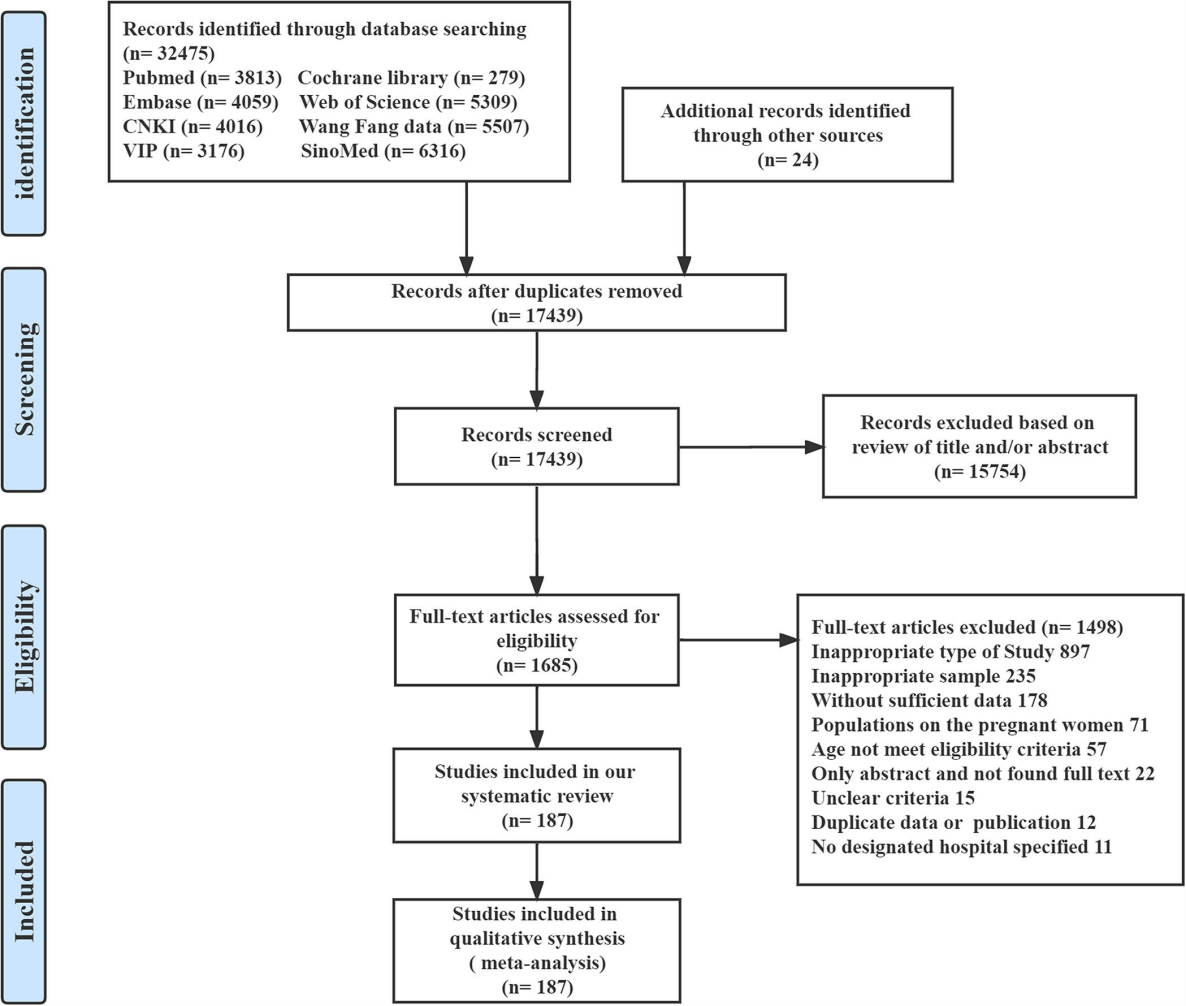
Summary of evidence search and selection. CNKI, China National Knowledge Infrastructure; Wan Fang Data, Wanfang Data Knowledge Service Platform; VIP, VIP information resource integration service platform databases; SinoMed, Chinese biomedical literature service system.

After 17,439 studies were initially screened and after the removal of duplicates, 187 studies reported the association between the comorbidities of COVID-19, complications of COVID-19, or both, and those reporting disease severity, progression, and mortality in the general populations with centralized isolation and hospitalization were included (Figure 1). All the studies were collected from hospital-based data, of which 89.84% (168 of 187) studies had a cohort study design, 8.02% (15 of 187) studies were cross-sectional studies, and the remaining 2.67% (5 of 187) studies were case–control studies. Among the included studies, there were 77,013 patients without COVID-19 vaccination in China between 2019 and 2020. Detailed patient characteristics of all the studies included are shown in Supplementary Table 1. The overall risk of bias, based on the JBI Checklist in 9 domains, was low in most studies (Supplementary Table 2). Among the 187 studies reporting comorbidities and complications in which the total prevalence and subgroup of gender ratio were reported, there were 54 different comorbidities and 46 various complications of COVID-19, which have been listed in Supplementary Tables 3, 4. We identified 85 clinical comorbidities and complications associated with disease severity, 31 with progression, and 58 with mortality in the meta-analysis.

### Disease severity

In disease severity, the most prevalent comorbidities with commonly reported conditions were hypertension [20.37% (95% CI 15.28–26.63), 19.29% (16.17–22.85), 34.72% (31.48–38.10), and 43.94% (38.94–49.06)] and diabetes [7.84% (5.78–10.54), 8.59% (7.25–10.16), 17.99% (16.29–19.84), and 22.68% (19.93–25.69)] in mild, moderate, severe, and critical patients, chronic heart disease [30.56% (17.80–47.21) and 44.44% (24.00–66.96)] in severe and critical patients, and cardiovascular diseases [28.24% (18.32–40.85)] in critical patients; the most prevalent complications were liver injury [10.00% (1.39–46.72), 23.04% (14.20–35.13), and 43.48% (39.88–47.15)] in mild, moderate, and severe patients and acute respiratory distress syndrome [ARDS; 94.17% (20.78–99.90)], respiratory failure [90.69% (28.08–99.59)], shock [61.54% (42.07–77.90)], and coagulopathy [59.09% (53.30–64.64)] in critical patients Supplementary Table 5). The pooled prevalence of comorbidities and complications associated with COVID-19 increased with advancing severity and was the highest in critical patients.

Most comorbidities and complications were positively associated with an increased risk of serious conditions in severe/critical patients (Table 1), with the strongest associations for renal insufficiency [OR 17.43 (95% CI 6.69–45.43)], cardiac insufficiency [12.69 (5.12–31.48)], and kidney failure [9.69 (1.62–57.81)] in comorbidities, with the strongest associations for respiratory diseases with ARDS [53.26 (30.72–92.33)] and respiratory failure [105.12 (49.48–223.33)], and critical illness with septic shock [58.03 (25.42–132.47)], multiple organ dysfunction syndrome [MODS; 29.96 (3.61–248.31)], and shock [26.35 (15.79–44.00)]. Moreover, we found that conditions coexisting with stroke [7.65 (1.58–36.92)], chronic obstructive pulmonary disease (COPD) [4.94 (1.00–24.38)], and coronary heart disease [4.91 (1.35–17.79)], and patients with shock [54.34 (12.47–236.89)], ARDS [34.49 (16.23–73.28)], and acute cardiac injury [33.85 (17.88–64.08)], were associated with a higher risk of ICU admission compared with that in non-ICU (Table 2). Heterogeneity was mostly low in the main analyses with respect to mild/moderate vs. severe/critical cases and ICU admission vs. non-ICU, and without any significant differences in subgroup analyses of gender for eligible strongest factors (Supplementary Tables 6, 7).

**Table 1:** Meta-analysis summary of comorbidities and complications for COVID-19 by mild/moderate vs. severe/critical.

**Comorbidities**	**OR (95% CI)**	***I* ^2^**	***p*-Value**	**Number of studies**	**Number of participants**	**Complications**	**OR (95% CI)**	***I* ^2^**	***p*-Value**	**Number of studies**	**Number of participants**
**Hypertension**	2.67 (2.40–2.97)	71.2%	<0.0001	95	39,742	**Acute cardiac injury**	7.92 (5.29–11.86)	51.0%	<0.0001	12	6,106
**Diabetes**	2.45 (2.20–2.72)	51.2%	<0.0001	97	40,410	**Arrhythmia**	16.12 (5.97–43.53)	62.2%	<0.0001	5	1,009
**Malignancy**	2.20 (1.88–2.57)	0.0%	<0.0001	64	30,964	**Atrial fibrillation**	18.16 (0.91–360.79)	NR	0.0573	1	126
**Cardiovascular diseases**	2.90 (2.43–3.47)	44.3%	<0.0001	37	13,332	**Cardiovascular injury**	11.18 (3.41–36.66)	NR	<0.0001	1	253
**Aorta sclerosis**	1.42 (0.09–23.19)	NR	0.8052	1	140	**Heart failure**	14.37 (6.22–33.20)	33.4%	<0.0001	4	5,302
**Arrhythmia**	3.90 (1.96–7.77)	0.0%	0.0001	4	781	**Myocardial injury**	20.06 (6.89–58.46)	44.0%	<0.0001	3	503
**Atrial fibrillation**	0.66 (0.09–4.88)	NR	0.6852	1	112	**ARDS**	53.26 (30.72–92.33)	48.0%	<0.0001	18	8,871
**Cardiac insufficiency**	12.69 (5.12–31.48)	0.0%	<0.0001	2	1,912	**Respiratory failure**	105.12 (49.48–223.33)	0.0%	<0.0001	5	5,986
**Chronic heart disease**	6.35 (3.75–10.76)	38.6%	<0.0001	9	3,583	**Respiratory injury**	12.32 (6.81–22.28)	0.0%	<0.0001	2	576
**Coronary artery disease**	4.04 (0.98–16.59)	74.6%	0.0528	2	1,073	**Abnormal kidney function**	2.10 (0.98–4.49)	NR	0.0569	1	462
**Coronary atherosclerosis**	2.23 (1.59–3.13)	NR	<0.0001	1	3,044	**Acute kidney injury**	7.45 (4.88–11.37)	60.7%	<0.0001	20	7,492
**Coronary heart disease**	2.86 (2.28–3.58)	47.6%	<0.0001	33	16,525	**Liver injury**	3.13 (2.02–4.86)	74.9%	<0.0001	9	3,892
**Heart failure**	17.54 (0.89–345.88)	NR	0.0597	1	172	**Electrolyte disturbance**	18.26 (2.25–148.07)	0.0%	0.0065	2	393
**Myocardial infarction**	3.99 (2.25–7.08)	NR	<0.0001	1	660	**Hyperglycemia**	3.03 (2.09–4.40)	NR	<0.0001	1	548
**Cerebrovascular disease**	2.91 (2.24–3.79)	36.7%	<0.0001	39	14,582	**Hypokalemia**	4.50 (1.17–17.37)	NR	0.0288	1	253
**Cerebral infarction**	3.85 (2.15–6.88)	18.1%	<0.0001	4	2,647	**Hypoproteinaemia**	14.14 (2.28–87.81)	89.9%	0.0045	2	2,145
**Intracerebral hemorrhage**	3.20 (0.90–11.41)	NR	0.073	1	1,767	**Bacteremia**	11.31 (3.98–32.16)	NR	<0.0001	1	548
**Stroke**	4.32 (2.10–8.90)	0.0%	<0.0001	4	1,616	**Bacterial infection**	4.30 (1.13–16.45)	59.8%	0.033	2	359
**Asthma**	1.57 (0.67–3.68)	0.0%	0.2991	3	5,359	**Fungi infection**	15.17 (1.09–210.76)	36.9%	0.0428	2	359
**Chronic bronchitis**	2.96 (0.24–37.08)	61.9%	0.3998	2	2,525	**Secondary infection**	18.59 (9.95–34.72)	0.0%	<0.0001	6	2,807
**Chronic lung disease**	2.03 (1.16–3.55)	49.5%	0.0127	14	3,702	**Sepsis**	15.14 (11.97–19.16)	0.0%	<0.0001	3	2,740
**COPD**	3.01 (2.52–3.60)	5.3%	<0.0001	49	24,791	**Septic shock**	58.03 (25.42–132.47)	0.0%	<0.0001	5	2,937
**Respiratory disease**	2.59 (1.94–3.45)	14.3%	<0.0001	16	6,010	**Anemia**	16.35 (2.18–122.73)	80.9%	0.0066	2	354
**Tuberculosis**	2.53 (0.71–9.05)	61.3%	0.1538	7	4,125	**Coagulopathy**	4.59 (0.86–24.38)	95.9%	0.0738	3	2,457
**Chronic kidney disease**	3.58 (2.63–4.87)	34.9%	<0.0001	43	20,103	**DIC**	14.25 (4.47–45.49)	15.4%	<0.0001	3	1,007
**Kidney failure**	9.69 (1.62–57.81)	0.0%	0.0127	2	294	**Thrombocytopenia**	2.81 (1.02–7.75)	NR	0.0461	1	3,044
**Renal insufficiency**	17.43 (6.69–45.43)	0.0%	<0.0001	6	2,997	**Thrombus**	5.05 (0.52–49.26)	53.3%	0.1638	2	3,297
**Nephritis**	1.24 (0.28–5.55)	NR	0.7777	1	3,044	**MODS**	29.96 (3.61–248.31)	0.0%	0.0016	2	329
**Gallbladder disease**	1.34 (0.31–5.72)	44.0%	0.6945	3	779	**Shock**	26.35 (15.79–44.00)	0.0%	<0.0001	12	2,242
**Chronic liver disease**	1.40 (1.10–1.78)	30.0%	0.0062	44	17,782	**Anaphylaxis**	3.49 (0.69–17.78)	NR	0.1323	1	253
**Cirrhosis**	2.13 (0.44–10.20)	67.6%	0.3447	3	5,134	**Pneumonthorax**	17.54 (0.89–345.88)	NR	0.0597	1	172
**Fatty liver**	1.73 (1.03–2.92)	0.0%	0.0388	4	992						
**Hepatitis B**	1.55 (0.74–3.24)	21.5%	0.2438	6	3,307						
**Gastrointestinal disease**	1.25 (0.81–1.92)	5.6%	0.3188	6	4,764						
**Peptic ulcer**	4.93 (0.43–55.84)	NR	0.1979	1	145						
**Gout**	6.33 (0.38–105.83)	NR	0.1989	1	134						
**Hyperlipidemia**	1.21 (0.72–2.04)	0.0%	0.4772	7	4,130						
**Hyperuricemia**	5.84 (0.59–57.44)	NR	0.1301	1	172						
**Thyroid disease**	1.98 (0.40–9.74)	54.0%	0.4019	5	1,125						
**Autoimmune disease**	2.33 (1.06–5.15)	0.0%	0.0359	7	2,372						
**Blood system diseases**	3.39 (0.38–30.49)	62.4%	0.2752	3	965						
**Bone disease**	1.33 (0.26–6.82)	NR	0.7298	1	238						
**Genital system diseases**	4.23 (1.32–13.54)	52.3%	0.015	2	546						
**Benign prostatic hyperplasia**	4.08 (1.68–9.95)	NR	0.002	1	3,044						
**Prostatitis**	2.79 (0.29–26.89)	NR	0.3738	1	3,044						
**Gynecological disease**	1.61 (0.30–8.56)	NR	0.5772	1	238						
**HIV infection**	1.87 (0.56–6.25)	0.0%	0.3118	7	1,099						
**Nervous system disease**	2.04 (0.84–4.92)	54.9%	0.1132	5	2,203						
**Rheumatism**	1.06 (0.06–17.49)	42.0%	0.9689	2	273						
**Urinary system disease**	0.89 (0.16–4.89)	77.5%	0.8912	2	1,075						
**Urolithiasis**	0.70 (0.06–7.93)	NR	0.7746	1	140						

I^2^ values were NR, only one study was available.

NR, not reportable; OR, odds ratio; 95% CI: 95% confidence interval; COPD, chronic obstructive pulmonary disease; ARDS, acute respiratory distress syndrome; DIC, disseminated intravascular coagulation; MODS, multiple organ dysfunction syndrome.

**Table 2:** Meta-analysis summary of comorbidities and complications for COVID-19 by ICU admission vs. non-ICU admission.

	**OR (95% CI)**	***I* ^2^**	***p*-Value**	**Number of studies**	**Number of participants**
**Comorbidities**
Hypertension	2.30 (1.77–3.01)	18.9%	<0.0001	10	3,003
Diabetes	2.20 (1.35–3.59)	58.7%	0.0016	10	3,003
Malignancy	4.11 (1.73–9.77)	42.8%	0.0014	5	1,743
Cardiovascular diseases	3.35 (2.30–4.86)	0.0%	<0.0001	6	2,158
Chronic heart disease	0.58 (0.07–4.67)	NR	0.6046	1	200
Coronary artery disease	2.04 (0.43–9.59)	NR	0.3672	1	416
Coronary heart disease	4.91 (1.35–17.79)	NR	0.0155	1	135
Myocardial infarction	0.94 (0.04–20.23)	NR	0.9687	1	135
Intracerebral hemorrhage	10.73 (0.43–270.28)	NR	0.1495	1	135
Stroke	7.65 (1.58–36.92)	NR	0.0113	1	135
Chronic lung disease	2.89 (0.39–21.21)	80.8%	0.2968	2	1,291
COPD	4.94 (1.00–24.38)	0.0%	0.0497	2	554
Tuberculosis	0.62 (0.04–10.95)	NR	0.7434	1	416
Chronic kidney disease	3.24 (0.93–11.30)	0.0%	0.0653	4	870
Chronic liver disease	0.82 (0.18–3.67)	0.0%	0.7982	4	952
Gout	1.14 (0.05–28.75)	NR	0.9357	1	135
Hyperlipidemia	5.05 (0.30–83.75)	NR	0.2588	1	135
Thyroid disease	1.35 (0.23–8.09)	0.0%	0.7399	2	333
HIV infection	0.55 (0.03–11.74)	NR	0.7024	1	138
**Complications**
Acute cardiac injury	33.85 (17.88–64.08)	0.0%	<0.0001	3	754
Arrhythmia	10.86 (3.95–29.83)	NR	<0.0001	1	138
Myocardial infarction	11.18 (0.68–182.68)	NR	0.0904	1	416
ARDS	34.49 (16.23–73.28)	0.0%	<0.0001	2	338
Acute kidney injury	10.17 (4.83–21.40)	0.0%	<0.0001	3	510
Liver injury	4.83 (1.80–13.01)	NR	0.0018	1	172
Bacterial infection	2.92 (1.11–7.68)	NR	0.0297	1	172
Secondary infection	4.88 (1.43–16.62)	NR	0.0112	1	200
MODS	7.71 (3.29–18.07)	NR	<0.0001	1	113
Shock	54.34 (12.47–236.89)	0.0%	<0.0001	3	510

I^2^ values were NR, only one study was available.

NR, not reportable; ICU, Intensive Care Unit; OR, odds ratio; 95% CI, 95% confidence interval; COPD, chronic obstructive pulmonary disease; ARDS, acute respiratory distress syndrome; MODS, multiple organ dysfunction syndrome.

### Disease progression

For disease progression, the most specific indicators of aggravation of illness (Table 3) during hospitalization were underlying comorbidities with low heterogeneity, and included autoimmune disease [5.10 (2.13–12.19)], nervous system disease [3.47 (1.71–7.06)], stroke [3.39 (1.74–6.61)], cerebrovascular disease [3.18 (1.47–6.88)], and cardiovascular diseases [3.16 (2.15–4.65)] and severe complications included disseminated intravascular coagulation (DIC) [85.43 (16.00–456.24)], ARDS [45.05 (6.13–331.08)], shock [43.31 (18.96–98.94)], and acute cardiac injury [42.83 (12.24–149.94)]. In subgroup analyses by sex, aggravation of illness associated with hypertension and diabetes was higher among those higher proportions of women than those higher proportions of men (Supplementary Table 8).

**Table 3:** Meta-analysis summary of comorbidities and complications for COVID-19 by progressive vs. non-progressive.

	**OR (95% CI)**	***I* ^2^**	***p*-Value**	**Number of studies**	**Number of participants**
**Comorbidities**
Hypertension	2.09 (1.54–2.83)	63.0%	<0.0001	14	5,301
Diabetes	1.82 (1.23–2.70)	63.0%	0.0029	13	4,936
Malignancy	1.85 (1.17–2.93)	0.0%	0.008	11	4,384
Cardiovascular diseases	3.16 (2.15–4.65)	16.8%	<0.0001	6	2,721
Chronic heart disease	1.41 (0.83–2.41)	NR	0.2009	1	1,102
Coronary heart disease	1.81 (0.94–3.46)	42.2%	0.0752	4	1,167
Cerebrovascular disease	3.18 (1.47–6.88)	0.0%	0.0032	6	1,587
Stroke	3.39 (1.74–6.61)	NR	0.0004	1	1,102
Chronic lung disease	2.25 (1.10–4.60)	0.0%	0.027	3	722
COPD	2.27 (1.41–3.63)	3.0%	0.0007	9	3,447
Respiratory disease	1.90 (1.16–3.10)	0.0%	0.0109	3	1,998
Tuberculosis	2.45 (0.84–7.13)	NR	0.1	1	1,102
Chronic kidney disease	2.44 (1.13–5.30)	22.0%	0.0238	7	2,694
Chronic liver disease	1.30 (0.71–2.38)	0.0%	0.404	6	2,223
Cirrhosis	0.58 (0.03–11.36)	NR	0.7192	1	323
Fatty liver	2.33 (0.78–7.00)	NR	0.1309	1	104
Gastrointestinal disease	1.11 (0.52–2.36)	NR	0.7802	1	663
Hyperlipidemia	0.59 (0.12–2.79)	NR	0.5013	1	114
Autoimmune disease	5.10 (2.13–12.19)	0.0%	0.0003	2	1,310
Nervous system disease	3.47 (1.71–7.06)	0.0%	0.0006	3	893
**Complications**
Acute cardiac injury	42.83 (12.24–149.94)	NR	<0.0001	1	323
Arrhythmia	12.61 (7.19–22.12)	0.0%	<0.0001	2	437
Myocardial injury	18.69 (5.81–60.19)	NR	<0.0001	1	114
ARDS	45.05 (6.13–331.08)	57.5%	0.0002	3	541
Respiratory injury	8.44 (4.57–15.59)	NR	<0.0001	1	323
Acute kidney injury	13.29 (4.53–38.98)	40.3%	<0.0001	2	437
Liver injury	0.97 (0.36–2.61)	NR	0.9577	1	114
Bacterial infection	5.33 (1.58–18.05)	NR	0.0071	1	104
DIC	85.43 (16.00–456.24)	NR	<0.0001	1	114
Shock	43.31 (18.96–98.94)	0.0%	<0.0001	2	437
Rhabdomyolysis	37.64 (7.19–196.93)	NR	<0.0001	1	114

I^2^ values were NR, only one study was available.

NR, not reportable; OR, odds ratio; 95%CI, 95% confidence interval; COPD, chronic obstructive pulmonary disease; ARDS, acute respiratory distress syndrome; DIC, disseminated intravascular coagulation.

### Mortality

Overall, the odds of death differed significantly between patients with comorbidities and complications vs. those without in most. The increased odds of mortality were strongly correlated with indicators in coexisting nervous system disease [5.28 (2.71–10.29)], respiratory disease [3.92 (2.38–6.45)], autoimmune disease [3.70 (1.74–7.87)], and cardiovascular diseases [3.35 (2.66–4.22)]. Cardiac and cerebrovascular diseases, respiratory system, infectious diseases, and critical illness-related complications were associated with increased mortality, and patients with shock [155.84 (49.43–491.40)], septic shock [93.23 (26.13–332.67)], and ARDS [63.99 (31.99–128.02)] have exhibited significant differences (*p* <0.0001) and had medium and high heterogeneity and greater odds compared with others. Besides, it should also be noted that other significantly higher risks of mortality, such as respiratory failure [34.54 (19.39–61.53)], gastrointestinal bleeding [31.40 (15.54–63.44)], secondary infection [30.00 (2.95–304.87)], sepsis [34.89 (7.27–167.34)], DIC [30.33 (9.54–96.48)], and thrombocytopenia [29.85 (7.35–121.24)], were observed. Data for comorbidities and complications of mortality are given in Table 4, and subgroup analysis showed that there were no significant differences among gender with respect to the rate of mortality (Supplementary Table 9).

**Table 4:** Meta-analysis summary of mortality for COVID-19 with comorbidities and complications vs. without comorbidities and complications.

**Comorbidities**	**OR (95% CI)**	***I* ^2^**	***p*-Value**	**Number of studies**	**Number of participants**	**Complications**	**OR (95% CI)**	***I* ^2^**	***p*-Value**	**Number of studies**	**Number of participants**
Hypertension	2.18 (1.92–2.48)	60.6%	<0.0001	64	24,661	Acute cardiac injury	25.01 (13.80–45.33)	83.3%	<0.0001	15	7,911
Diabetes	1.97 (1.73–2.25)	49.9%	<0.0001	66	25,919	Arrhythmia	24.01 (5.44–105.98)	82.6%	<0.0001	3	478
Malignancy	2.66 (2.16–3.27)	5.2%	<0.0001	49	20,010	Heart failure	10.21 (4.30–24.20)	78.0%	<0.0001	7	1,682
Cardiovascular diseases	3.35 (2.66–4.22)	42.9%	<0.0001	24	11,274	Myocardial infarction	25.88 (2.96–226.38)	0.0%	0.0033	2	362
Chronic heart disease	2.34 (1.37–3.99)	61.20%	0.0017	7	2,877	Myocardial injury	23.31 (15.35–35.39)	0.0%	<0.0001	3	917
Coronary artery disease	3.00 (1.77–5.10)	NR	<0.0001	1	681	Stroke	17.51 (4.28–71.57)	0.0%	<0.0001	3	572
Coronary heart disease	2.58 (2.02–3.29)	54.7%	<0.0001	29	10,159	ARDS	63.99 (31.99–128.02)	87.4%	<0.0001	20	10,834
Cerebrovascular disease	2.86 (2.19–3.74)	33.4%	<0.0001	30	12,236	Respiratory failure	34.54 (19.39–61.53)	74.3%	<0.0001	12	7,754
Stroke	2.75 (1.60–4.71)	0.0%	0.0002	3	2,642	Acute kidney injury	16.56 (8.53–21.18)	84.5%	<0.0001	17	8,067
Chronic bronchitis	0.85 (0.35–2.08)	0.0%	0.7199	2	740	kidney failure	9.78 (0.99–96.58)	59.0%	0.0509	2	328
Chronic lung disease	2.30 (1.81–2.93)	0.0%	<0.0001	15	8,944	Liver injury	3.31 (1.88–5.82)	79.2%	<0.0001	11	5,833
COPD	3.02 (2.37–3.85)	17.1%	<0.0001	38	14,012	Gastrointestinal bleeding	31.40 (15.54–63.44)	0.0%	<0.0001	6	4,675
Respiratory disease	3.92 (2.38–6.45)	31.9%	<0.0001	7	2,532	Acidosis	10.93 (1.09–109.67)	76.3%	0.042	4	468
Tuberculosis	3.37 (1.13–9.98)	NR	0.0287	1	1,190	Alkalosis	2.26 (0.41–12.39)	28.3%	0.347	2	163
Pulmonary emphysema	2.69 (0.24–30.56)	NR	0.4239	1	118	Electrolyte disturbance	13.93 (5.69–34.12)	NR	<0.0001	1	2,079
Chronic kidney disease	2.96 (2.20–3.97)	41.2%	<0.0001	41	16,281	Hyperkalaemia	3.74 (2.07–6.74)	NR	<0.0001	1	274
kidney failure	3.14 (0.44–22.57)	50.3%	0.2549	2	338	Bacteremia	2.59 (0.54–12.47)	NR	0.2354	1	239
Uremia	2.72 (1.09–6.82)	NR	0.0327	1	511	Bacterial infection	6.11 (2.22–16.83)	88.4%	0.0005	6	2,594
Chronic liver disease	1.46 (1.11–1.92)	0.0%	0.0066	26	9,678	Fungi infection	5.66 (1.04–30.78)	68.2%	0.0448	3	844
Cirrhosis	1.56 (0.17–14.13)	NR	0.694	1	511	Secondary infection	30.00 (2.95–304.87)	90.8%	0.004	3	1,096
Hepatitis B	1.20 (0.36–4.02)	NR	0.7723	1	274	Sepsis	34.89 (7.27–167.34)	84.9%	<0.0001	10	4,697
Gastrointestinal disease	0.79 (0.17–3.72)	0.0%	0.7611	2	937	Septic shock	93.23 (26.13–332.67)	81.1%	<0.0001	8	6,135
Thyroid disease	0.98 (0.16–5.94)	NR	0.9791	1	281	Coagulopathy	12.71 (4.72–34.18)	79.2%	<0.0001	5	2,115
Autoimmune disease	3.70 (1.74–7.87)	0.0%	0.0007	4	1,787	DIC	30.33 (9.54–96.48)	4.1%	<0.0001	4	3,089
HIV infection	1.26 (0.06–26.75)	NR	0.8836	1	245	Thrombocytopenia	29.85 (7.35–121.24)	NR	<0.0001	1	432
Nervous system disease	5.28 (2.71–10.29)	0.0%	<0.0001	2	910	Shock	155.84 (49.43–491.40)	59.9%	<0.0001	10	4,930
Urinary system disease	0.56 (0.03–9.56)	NR	0.691	1	663	Pneumonthorax	9.82 (0.39–244.63)	NR	0.1639	1	220
Urinary tract infection	2.55 (0.28–23.13)	NR	0.4067	1	239						

I^2^ values were NR, only one study was available.

NR, not reportable; OR, odds ratio; 95% CI: 95% confidence interval; COPD, chronic obstructive pulmonary disease; ARDS, acute respiratory distress syndrome; DIC, disseminated intravascular coagulation.

## Discussion

In this systematic review and meta-analysis that included 187 studies with 77,013 patients without COVID-19 vaccination from China, a large proportion of inpatients with COVID-19 were found to have various coexisting comorbidities and complications. Moreover, comorbidities and complications were strongly associated with a higher risk of severe and critical outcomes, worsening illness, ICU admissions, and even mortality during centralized isolation and hospitalization.

The most prevalent comorbidities were hypertension in mild, moderate, severe, and critical patients, followed by diabetes and cardiovascular diseases. However, the most prevalent complications differed, and liver and renal complications were more common in mild and moderate patients, whereas severe respiratory complications were more common in severe and critical patients. Our results were in accordance with findings from previous reviews that patients with COVID-19 have a highly estimated prevalence of coexisting hypertension, diabetes, or cardiovascular diseases (26–28). Our results for cardiovascular disease findings were more detailed than previous findings, indicating that the leading risk factors were chronic heart disease and cardiac insufficiency in severe/critical patients. However, the higher odds of kidney failure and renal insufficiency should be paid attention to patients with COVID-19 who are in a serious condition, as these comorbidities have been neglected overall due to their lower prevalence (26). Although stroke and COPD had a significant OR in ICU hospitalization, our results should be interpreted with caution due to the small sample size. In several studies, the cerebrovascular disease has also been identified as a higher risk factor for the patients with COVID-19 (29, 30).

In addition to summarizing various disease severities of COVID-19, we explored disease progression and mortality due to complications that are highly associated with COVID-19. We observed the potential harms for aggravation of illness with exceptional odds during hospitalization, in the presence of comorbidities such as autoimmune disease, nervous system disease, and cerebrovascular and cardiovascular diseases, which were consistent with those reported by several studies (29, 31, 32). Some included studies with small sample sizes reported stroke among present comorbidities, which might be overestimated with high odds during disease progression. This finding suggested that most cardio- and cerebrovascular diseases, respiratory system diseases, kidney diseases, nervous system diseases, and autoimmune diseases can increase the risk of death in patients by presumably 3- to 5-fold than in those without these comorbidities. While a higher risk of death might be overreported in our study with respect to comorbidities due to the sample size limitation, these factors have also been reported by others in association with the increased risk of COVID-19 (33–35). Strong evidence along with larger patient populations in our study shows that hypertension, diabetes, malignancy, and cardiovascular disease contributed significantly with 2–4 OR values to ICU hospitalization, disease progression, and death (Tables 2–4), and is in accordance with the findings confirmed by other studies (29).

Our results are largely consistent with those of published studies and provide more precise estimates of pooled prevalence and comparisons for complications (7, 36, 37). ARDS, respiratory failure, septic shock, MODS, and shock were likely to appear in serious episodes of COVID-19 with extremely higher risks compared with the mild and moderate forms. In addition, complications with shock, ARDS, and acute cardiac injury were found to significantly increase the risks of ICU hospitalization and progression. In particular, ARDS and shock were the strongly correlated indicators of deterioration, and also pose a substantial risk of death. ARDS and respiratory failure, as severe, persistent respiratory complications of COVID-19, have been confirmed to pose higher risks of severe and critical illness, and even death, which may trigger substantial morbidity and halt disease progression with more complex effects in the patient population (38, 39). Our study has fully considered and explained the association between COVID-19 and its complications, and the results could serve as a means to find efficient methods of prevention to reduce long-term morbidity and mortality (40).

### Strengths and limitations

To the best of our knowledge, this is the first study to explore the clinical factors and identify the most comprehensive fatal comorbidities and complications of COVID-19 in China. Consideration of comorbidities and complications is among the factors essential for clinical decision-making in the patient population with COVID-19, and to provide data for targeted prevention and treatment strategies in the healthcare system during centralized isolation and in a hospital setting. Moreover, low heterogeneity was determined using the *I*^2^ statistic in most disease severities in the estimated proportions and between-study comparisons of disease severity, progression, and mortality. To determine the potential heterogeneity in certain clinical factors across studies, we conducted subgroup analyses to further explore and interpret outcomes. Our systematic review and meta-analysis and robust subgroup analyses provide a comprehensive overview. The large sample size allowed us to examine various underlying comorbidities and complications and estimate pooled proportions and comparisons.

Our study has several limitations. There was heterogeneity in the definition of comorbidities and complications across studies, which was identified using retrospective observational data in the primary research. Second, subgroup analyses could not be conducted for age to estimate and interpret clinical heterogeneity, because information on age for each comorbidity and complication for patients with COVID-19 was not available. Moreover, there were no significant differences in the median age. Despite our meta-analysis and subgroup analysis for all the comorbidities and complications, we were unable to quantify the effects of treatment factors on individual variability, especially in disease progression. Additionally, our results were also affected by the potential merge mixing of comorbidities and complications, publication bias due to small sample sizes, and wide CIs on clinical factors, all of which may have exaggerated the pooled proportions and OR value of comparisons for actual outcomes in a single disease. Our results did not reveal any association and outcome analysis in vaccinated and nonvaccinated individuals in China. Because these analyses were focused on the general populations who had not received COVID-19 vaccines; thus, our results demonstrate the real findings with respect to comorbidities and complications in primary infections without vaccine protection. Because the vast majority of included studies not mentioned the impact of treatment with monoclonal antibodies, antiviral, or peripheral immune plasma therapy, which can potentially impact the stability of results in complications.

## Conclusion

In this study, substantially greater harm was found among inpatients with COVID-19 with comorbidities and complications who were severe and critical, had ICU hospitalization, showed aggravation in disease progression, and mortality compared with those without. Comprehensively identifying the comorbidities and complications of the inpatients with COVID-19 could improve the precision of risk predictions and further enhance the possibility of disease prevention, exacerbations, and death. Although the estimated proportions and comparisons are limited by generalizability, our findings summarize all the available data in China to further refine the burden of disease estimates for COVID-19 in terms of centralized isolation and hospitalization. High-quality studies are needed to better estimate the comorbidities and complications of COVID-19 that are relevant to disease severity, progression, and mortality.

## Data availability statement

The original contributions presented in the study are included in the article/Supplementary material, further inquiries can be directed to the corresponding authors.

## Author contributions

ZC, YP, and CL designed and conceived the study. ZC, YP, XW, and BP contribute to the systematic search. ZC and YP contribute to the selection of eligible studies, curated and interpreted the data, contribute to the statistical analyses, and drafted the manuscript. BP and WZ contribute to the risk-of-bias assessment. XW, BP, FY, WZ, CL, and JZ verified the data. BP, FY, WZ, CL, and JZ revised and supervised the manuscript. CL and JZ contribute to full access to all of the data in the study and take responsibility for the integrity of the data and the accuracy of the data analysis. All the authors have reviewed and approved the final manuscript.

## Funding

This study was supported by Research on establishment and evaluation of evidence chain of traditional Chinese medicine for COVID-19 based on core outcome set (82074583).

## Conflict of interest

The authors declare that the research was conducted in the absence of any commercial or financial relationships that could be construed as a potential conflict of interest.

## Publisher's note

All claims expressed in this article are solely those of the authors and do not necessarily represent those of their affiliated organizations, or those of the publisher, the editors and the reviewers. Any product that may be evaluated in this article, or claim that may be made by its manufacturer, is not guaranteed or endorsed by the publisher.
