# Supplementary material for: Comorbidities and complications of COVID-19 associated with disease severity, progression, and mortality in China with centralized isolation and hospitalization: A systematic review and meta-analysis
Source: Front Public Health. 2022 Aug 16;10:923485. doi: 10.3389/fpubh.2022.923485 (PMC9424916; doi:10.3389/fpubh.2022.923485)
Supplement: Supplementary file 1 [file Data_Sheet_1.DOCX]

**Supplementary Tables and file**

[Supplementary Table 1 Characteristics and references of included studies 2](#_Toc101267631)

[Supplementary Table 2 Quality assessment by Joanna Briggs Institute Prevalence Critical Appraisal Checklist 34](#_Toc101267632)

[Supplementary Table 3 Pooled estimate prevalence of comorbidities and complications for COVID-19 in total 40](#_Toc101267633)

[Supplementary Table 4 Subgroup of gender ratio for comorbidities and complications of COVID-19 in total 44](#_Toc101267634)

[Supplementary Table 5 Pooled estimate prevalence of comorbidities and complications for COVID-19 in mild, moderate, severe, and critical group 46](#_Toc101267635)

[Supplementary Table 6 Subgroup of gender ratio for comorbidities and complications of COVID-19 in mild/moderate versus severe/critical 49](#_Toc101267636)

[Supplementary Table 7 Subgroup of gender ratio for comorbidities and complications of COVID-19 in ICU admission versus non- ICU admission 50](#_Toc101267637)

[Supplementary Table 8 Subgroup of gender ratio for comorbidities and complications of COVID-19 in progressive versus non-progressive 51](#_Toc101267638)

[Supplementary Table 9 Subgroup of gender ratio in mortality for COVID-19 with comorbidities and complications vs without comorbidities and complications 52](#_Toc101267639)

[Supplementary File 1 The detailed strategies in English and Chinese language databases 53](#_Toc101267640)

# Supplementary Table 1 Characteristics and references of included studies

| Author Year | Study month date of patient admission | Sample size | Male | Female | Age | Type Study | Multi center (M) or Single center (S) | Disease diagnosis and severity | City |
| --- | --- | --- | --- | --- | --- | --- | --- | --- | --- |
| An YW 2021 | 2020.1.11-2020.2.21 | 253 | 113 | 140 | 45.3 | Retrospective cohort study | S | World Health Organization interim guidance | Shenzhen |
| Cai QX 2020a | 2020.1.11-2020.2.6 | 298 | 145 | 153 | 47.5 | Retrospective cohort study | S | World Health Organization interim guidance | Shenzhen |
| Cai QX 2020b | 2020.1.11-2020.2.16 | 383 | 183 | 200 | 48.42 | Retrospective cohort study | S | World Health Organization interim guidance | Shenzhen |
| Caillon A 2021 | 2020.1.23-2020.2.23 | 157 | 75 | 82 | 59.77 | Retrospective cohort study | S | World Health Organization interim guidance | Wuhan |
| Cao JL 2020 | 2020.1.3-2020.2.1 | 102 | 53 | 49 | 54 | Retrospective cohort study | S | World Health Organization interim guidance | Wuhan |
| Cao M 2020 | 2020.1.20-2020.2.15 | 198 | 101 | 97 | 50.1 | Retrospective cohort study | S | The COVID-19 diagnosis and treatment guide of the National Health Commission in China | Shanghai |
| Cao YK 2020 | 2020.1.5-2020.2.22 | 101 | 67 | 34 | 56.6 | Retrospective cohort study | D | World Health Organization interim guidance | Wuhan |
| Chen FF 2020 | 2020.1.3-2020.4.9 | 681 | 362 | 319 | 65 | Retrospective cohort study | S | The COVID-19 diagnosis and treatment guide of the National Health Commission in China | Wuhan |
| Chen HL 2021 | 2020.1.27-2020.2.12 | 726 | 393 | 333 | 65.72 | Retrospective cohort study | S | World Health Organization interim guidance | Wuhan |
| Chen L 2020a | 2020.1.15-2020.3.26 | 792 | 432 | 360 | 55 | Retrospective cohort study | D | World Health Organization interim guidance | Hubei |
| Chen L 2020b | 2020.1.1-2020.2.29 | 245 | 114 | 131 | 55 | Retrospective cross-sectional study | S | World Health Organization interim guidance | Wuhan |
| Chen QQ 2020 | 2020.1.1-2020.3.11 | 145 | 79 | 66 | 47.52 | Retrospective cross-sectional study | S | World Health Organization interim guidance | Taizhou |
| Chen T 2020 | 2020.1.13-2020.2.12 | 274 | 171 | 103 | 62 | Retrospective cohort study | S | The COVID-19 diagnosis and treatment guide of the National Health Commission in China | Wuhan |
| Chen Y 2021 | 2020.2.4-2020.3.21 | 1578 | 779 | 799 | 60 | Retrospective cohort study | S | The COVID-19 diagnosis and treatment guide of the National Health Commission in China | Wuhan |
| Chen YY 2021 | 2020.1.28-2020.3.13 | 192 | 124 | 68 | 59.54 | Retrospective cohort study | S | World Health Organization interim guidance | Huanggang |
| Cheng B 2020 | 2020.1.1-2020.3.20 | 456 | 211 | 245 | 54.97 | Retrospective cohort study | S | The COVID-19 diagnosis and treatment guide of the National Health Commission in China | Wuhan |
| Cheng KB 2021 | 2020.1.1-2020.2.6 | 471 | 250 | 221 | 51 | Retrospective cohort study | S | The COVID-19 diagnosis and treatment guide of the National Health Commission in China | Wuhan |
| Deng Q 2020 | 2020.1.6-2020.2.20 | 112 | 57 | 55 | 65 | Retrospective cohort study | S | World Health Organization interim guidance and The COVID-19 diagnosis and treatment guide of the National Health Commission in China | Wuhan |
| Deng Y 2020 | 2020.1.1-2020.2.21 | 225 | 124 | 101 | 54.5 | Retrospective cohort study | D | The COVID-19 diagnosis and treatment guide of the National Health Commission in China | Wuhan |
| Ding RR 2021 | 2020.1.20-2020.3.15 | 311 | 162 | 149 | 51 | Retrospective cohort study | S | The COVID-19 diagnosis and treatment guide of the National Health Commission in China | Shanghai |
| Fan HZ 2021 | 2020.1-2020.4 | 288 | 147 | 141 | 53.7 | Retrospective cohort study | S | The COVID-19 diagnosis and treatment guide of the National Health Commission in China | Huangshi |
| Fang X 2020 | 2020.2.9-2020.3.9 | 506 | 264 | 242 | 58 | Retrospective cohort study | S | The COVID-19 diagnosis and treatment guide of the National Health Commission in China | Wuhan |
| Feng XB 2020 | 2020.1.23-2020.2.22 | 114 | 71 | 43 | 63.93 | Retrospective cohort study | S | World Health Organization interim guidance and The COVID-19 diagnosis and treatment guide of the National Health Commission in China | Wuhan |
| Feng Y 2020 | 2020.1.1-2020.2.15 | 476 | 271 | 205 | 53 | Retrospective cohort study | M | The COVID-19 diagnosis and treatment guide of the National Health Commission in China | Wuhan, Shanghai, Anhui |
| Gao S 2020 | 2020.1.23-2020.2.29 | 210 | 101 | 109 | 71 | Retrospective cohort study | S | World Health Organization interim guidance | Wuhan |
| Guo XY 2020 | 2020.1.13-2020.02 | 195 | 94 | 101 | 49.24 | Retrospective cohort study | M | The COVID-19 diagnosis and treatment guide of the National Health Commission in China | Guangzhou, Meizhou,and Foshan |
| Hu L 2020 | 2020.1.8-2020.2.20 | 323 | 166 | 157 | 61 | Retrospective cohort study | S | World Health Organization interim guidance and The COVID-19 diagnosis and treatment guide of the National Health Commission in China | Wuhan |
| Hu Q 2021a | 2020.2.3-2020.4.14 | 727 | 391 | 336 | 65 | Retrospective cohort study | S | The COVID-19 diagnosis and treatment guide of the National Health Commission in China | Wuhan |
| Hu Q 2021b | 2020.1.20-2020.5.6 | 462 | 240 | 222 | 48 | Retrospective cohort study | D | The COVID-19 diagnosis and treatment guide of the National Health Commission in China | Chongqing |
| Hu XS 2020 | 2020.1.24-2020.2.16 | 213 | 102 | 111 | 44 | Retrospective cohort study | D | The COVID-19 diagnosis and treatment guide of the National Health Commission in China | Changsha and Xiangtan |
| Huang R 2020 | 2020.1.22-2020.2.10 | 202 | 116 | 86 | 44 | Retrospective cohort study | M | World Health Organization interim guidance | Xuzhou, Lianyungang, Suqian, Huaian, Yancheng, Yangzhou, Changzhou, and Suzhou |
| Huang YX 2020 | 2020.1.22-2020.2.14 | 238 | 116 | 122 | 45±17 | Retrospective cohort study | S | World Health Organization interim guidance | Changsha |
| Jiang Y 2020 | 2020.1.30-2020.3.8 | 281 | 143 | 138 | 71.76 | Retrospective cohort study | S | World Health Organization interim guidance | Wuhan |
| Jiao L 2020 | 2020.1.10-2020.3.3 | 126 | 60 | 66 | 57.15 | Retrospective cohort study | S | The COVID-19 diagnosis and treatment guide of the National Health Commission in China | Wuhan |
| Li HB 2020 | 2020.1.18-2020.2.3 | 188 | 112 | 76 | 57.5 | Retrospective cohort study | S | RT-PCR | Wuhan |
| Li HY 2020 | 2020.2.1-2020.2.29 | 132 | 74 | 58 | 58.8 | Retrospective cross-sectional study | S | World Health Organization interim guidance and The COVID-19 diagnosis and treatment guide of the National Health Commission in China | Wuhan |
| Li J 2021 | 2020.1.8-2020.3.17 | 326 | 171 | 155 | 62 | Retrospective case control study | S | The COVID-19 diagnosis and treatment guide of the National Health Commission in China | Wuhan |
| Li JW 2021 | 2020.2.4-2020.4.7 | 2719 | 1368 | 1351 | 61 | Retrospective cohort study | M | The COVID-19 diagnosis and treatment guide of the National Health Commission in China | Hubei |
| Li LZ 2021 | 2020.2.1-2020.3.25 | 113 | 68 | 45 | 67.25 | Retrospective cross-sectional study | S | The COVID-19 diagnosis and treatment guide of the National Health Commission in China | Wuhan |
| Li M 2020 | 2019.12.26-2020.2.15 | 245 | 118 | 127 | 54 | Retrospective cohort study | D | The COVID-19 diagnosis and treatment guide of the National Health Commission in China | Wuhan |
| Li N 2020 | 2020.2.8-2020.2.18 | 138 | 71 | 67 | 62 | Retrospective cohort study | S | World Health Organization interim guidance | Wuhan |
| Li PP 2021 | 2020.2.4-2020.4.10 | 2954 | 1461 | 1493 | 60 | Retrospective cohort study | S | The COVID-19 diagnosis and treatment guide of the National Health Commission in China | Wuhan |
| Li QB 2020 | 2020.1.20-2020.4.4 | 1449 | 733 | 716 | 57 | Retrospective cohort study | M | World Health Organization interim guidance and The COVID-19 diagnosis and treatment guide of the National Health Commission in China | Wuhan |
| Li RQ 2020 | 2020.1.21-2020.3.2 | 193 | 112 | 81 | 50.7 | Retrospective cohort study | D | The COVID-19 diagnosis and treatment guide of the National Health Commission in China | Xiaogan and Wuhan |
| Li T 2020 | 2020.2.1-2020.3.31 | 312 | 187 | 125 | 69.2 | Retrospective Cohort Study | S | World Health Organization interim guidance | Wuhan |
| Li WX 2021 | 2020.1.20-2020.11.30 | 1249 | 773 | 476 | 36 | Retrospective cohort study | S | The COVID-19 diagnosis and treatment guide of the National Health Commission in China | Shanghai |
| Li XC 2020 | 2020.1.26-2020.2.5 | 548 | 279 | 269 | 60 | Ambispective Cohort Study | S | The COVID-19 diagnosis and treatment guide of the National Health Commission in China | Wuhan |
| Li XQ 2021 | 2020.1.13-2020.4.8 | 223 | 130 | 93 | 55 | Retrospective cohort study | M | The COVID-19 diagnosis and treatment guide of the National Health Commission in China | Wuhan and Guiyang |
| Li Y 2020 | 2020.2.10-2020.2.26 | 135 | 69 | 66 | 64 | Retrospective cohort study | S | The COVID-19 diagnosis and treatment guide of the National Health Commission in China | Wuhan |
| Li Y 2021a | 2020.1.29-2020.4.8 | 465 | 248 | 217 | 62 | Retrospective cohort study | S | World Health Organization interim guidance | Wuhan |
| Li Y 2021b | 2020.1.29-2020.4.8 | 424 | 220 | 204 | 60.7 | Retrospective cohort study | S | World Health Organization interim guidance | Wuhan |
| Liao DY 2020 | 2020.1.23-2020.2.23 | 380 | 206 | 174 | 64 | Retrospective cohort study | S | World Health Organization interim guidance | Wuhan |
| Lin HY 2021 | 2020.2.19-2020.3.23 | 1767 | 740 | 1027 | 58.83 | Retrospective cohort study | S | The COVID-19 diagnosis and treatment guide of the National Health Commission in China | Wuhan |
| Lin L 2020 | 2020.1.20-2020.2.16 | 243 | 124 | 119 | 49.38 | Retrospective cohort study | S | The COVID-19 diagnosis and treatment guide of the National Health Commission in China | Guangzhou |
| Liu D 2020 | 2020.1.27-2020.3.21 | 2044 | 1000 | 1044 | 62 | Retrospective cohort study | S | The COVID-19 diagnosis and treatment guide of the National Health Commission in China | Wuhan |
| Liu FY 2020 | 2020.1.30-2020.3.14 | 673 | 341 | 332 | 61 | Retrospective cohort study | S | World Health Organization interim guidance | Wuhan |
| Liu J 2020 | 2019.12.29-2020.2.28 | 1190 | 635 | 555 | 57 | Retrospective cohort study | S | World Health Organization interim guidance | Wuhan |
| Liu J 2021 | 2020.1.30-2020.2.20 | 214 | 119 | 95 | 68 | Retrospective cohort study | S | World Health Organization interim guidance | Wuhan |
| Liu JC 2021 | 2020.1.13-2020.2.25 | 122 | 72 | 50 | 62 | Retrospective case control study | S | World Health Organization interim guidance | Wuhan |
| Liu MY 2021 | 2020.1.1-2020.3.4 | 665 | 318 | 347 | 58 | Retrospective cohort study | M | The COVID-19 diagnosis and treatment guide of the National Health Commission in China | Wuhan |
| Liu QQ 2020 | 2020.1.10-2020.2.13 | 308 | 166 | 142 | 62.82 | Retrospective cohort study | S | The COVID-19 diagnosis and treatment guide of the National Health Commission in China | Wuhan |
| Liu SJ 2020 | 2020.1.23-2020.2.12 | 146 | 84 | 62 | 64.95 | Retrospective cohort study | S | The COVID-19 diagnosis and treatment guide of the National Health Commission in China | ezhou |
| Liu XQ 2022 | 2020.2.23-2020.4.4 | 101 | 56 | 45 | 62 | Retrospective cohort study | S | The COVID-19 diagnosis and treatment guide of the National Health Commission in China | Wuhan |
| Liu Z 2021 | 2020.2.6-2020.3.9 | 149 | 93 | 56 | 64.73 | Retrospective cohort study | S | The COVID-19 diagnosis and treatment guide of the National Health Commission in China | Wuhan |
| Liu ZZ 2020 | 2020.2.4-2020.3.21 | 2391 | 1210 | 1181 | 59 | Retrospective cohort study | S | The COVID-19 diagnosis and treatment guide of the National Health Commission in China | Wuhan |
| Long X 2020 | 2019.12.31-2020.3.1 | 108 | 73 | 35 | 66 | Retrospective cohort study | S | World Health Organization interim guidance | Wuhan |
| Lu ZL 2020 | 2020.1.15-2020.2.15 | 101 | 34 | 67 | 47.94 | Retrospective cross-sectional study | S | The COVID-19 diagnosis and treatment guide of the National Health Commission in China | Wuhan |
| Luo M 2020 | 2020.1.9-2020.3.31 | 1018 | 521 | 497 | 61 | Retrospective cohort study | D | World Health Organization interim guidance | Wuhan |
| Lv ZH 2020 | 2020.2.4-2020.2.28 | 354 | 175 | 179 | 62 | Retrospective cohort study | S | World Health Organization interim guidance | Wuhan |
| Mi J 2020 | 2020.2.10-2020.3.28 | 189 | 102 | 87 | 63 | Retrospective cohort study | S | The COVID-19 diagnosis and treatment guide of the National Health Commission in China | Wuhan |
| Mo J 2020 | 2020.1.17-2020.3.14 | 208 | 105 | 103 | 44 | Retrospective cohort study | S | The COVID-19 diagnosis and treatment guide of the National Health Commission in China | Changsha |
| Pan D 2020 | 2020.2.9-2020.3.10 | 120 | 70 | 50 | 65 | Retrospective cohort study | S | RT-PCR | Wuhan |
| Pei GC 2020 | 2020.1.28-2020.2.9 | 333 | 182 | 151 | 56.3 | Retrospective cohort study | S | The COVID-19 diagnosis and treatment guide of the National Health Commission in China | Wuhan |
| Peng F 2021 | 2020.by3.26 | 622 | 318 | 304 | 55 | Retrospective cohort study | D | The COVID-19 diagnosis and treatment guide of the National Health Commission in China | Changsha and Wuhan |
| Peng L 2021 | 2020.1.11-2020.3.8 | 205 | 98 | 107 | 58.4 | Retrospective cohort study | S | RT-PCR | Wuhan |
| Peng S 2020 | 2020.1.16-2020.3.28 | 226 | 111 | 115 | 63.36 | Retrospective cohort study | S | The COVID-19 diagnosis and treatment guide of the National Health Commission in China | Wuhan |
| Qi X 2021 | 2020.2.2-2020.6.25 | 107 | 54 | 53 | 48.86 | Retrospective cohort study | M | The COVID-19 diagnosis and treatment guide of the National Health Commission in China | Wuhan, Suzhou and Xuzhou |
| Qin SJ 2021 | 2020.2.4-2020.4.13 | 3044 | 1546 | 1498 | 60 | Retrospective cohort study | S | The COVID-19 diagnosis and treatment guide of the National Health Commission in China | Wuhan |
| Qin ZJ 2020 | 2020.2.4-2020.3.4 | 118 | 49 | 69 | 63.1 | Retrospective cohort study | S | The COVID-19 diagnosis and treatment guide of the National Health Commission in China | Wuhan |
| Qu JJ 2020 | 2020.1.24-2020.3.14 | 246 | 115 | 131 | 53.63 | Retrospective cohort study | M | The COVID-19 diagnosis and treatment guide of the National Health Commission in China and American Thoracic Society guidelines | Wuhan, Huanggang, Linyi |
| Rao XR 2020 | 2019.12.24-2020.3.25 | 240 | 111 | 129 | 48 | Retrospective cohort study | S | World Health Organization interim guidance | Wuhan |
| Ren LY 2020 | 2020.1.20-2020.2.4 | 129 | 62 | 67 | 50 | Retrospective cohort study | S | The COVID-19 diagnosis and treatment guide of the National Health Commission in China | Wuhan |
| Ren MX 2020 | 2020.1.21-2020.3.31 | 103 | 45 | 58 | 51.06 | Retrospective cohort study | S | The COVID-19 diagnosis and treatment guide of the National Health Commission in China | Beijing |
| Shang YF 2020 | 2020.1.1-2020.3.27 | 113 | 73 | 40 | 66 | Retrospective cohort study | M | World Health Organization interim guidance | Wuhan |
| Shen JX 2021 | 2020.1.17-2020.2.26 | 356 | 193 | 163 | 52.1 | Retrospective cohort study | S | World Health Organization interim guidance | Yichang |
| Shi PY 2020 | 2020.1.23-2020.3.7 | 134 | 65 | 69 | 46 | Retrospective cohort study | M | The COVID-19 diagnosis and treatment guide of the National Health Commission in China | Xian, Ankang, Baoji, Hanzhong, Weinan, Xianyang, Shangluo, Yanan, and Tongchuan |
| Shi Q 2020 | 2020.1.1-2020.3.8 | 306 | 150 | 156 | 64.5 | Retrospective cohort study | D | World Health Organization interim guidance | Wuhan |
| Shu ZX 2020 | 2020.1.15-2020.3.2 | 293 | 135 | 158 | 57.1 | Retrospective cohort study | S | The COVID-19 diagnosis and treatment guide of the National Health Commission in China | Wuhan |
| Song FE 2021 | 2020.1-2020.5 | 295 | 155 | 140 | 58 | Retrospective cross-sectional study | D | The COVID-19 diagnosis and treatment guide of the National Health Commission in China | Wuhan |
| Sun F 2021 | 2019.12.19-2020.2.2 | 165 | 84 | 81 | 55 | Retrospective cohort study | S | World Health Organization interim guidance | Wuhan |
| Sun HY 2020 | 2020.1.29-2020.3.5 | 244 | 133 | 111 | 69.48 | Retrospective case control study | S | The COVID-19 diagnosis and treatment guide of the National Health Commission in China | Wuhan |
| Sun JH 2020 | 2020.1.14-2020.3.29 | 939 | 453 | 486 | 62 | Retrospective cohort study | S | The COVID-19 diagnosis and treatment guide of the National Health Commission in China | Wuhan |
| Sun Y 2020 | 2020.1.21-2020.3.4 | 168 | 95 | 73 | 42.6 | Retrospective cohort study | D | The COVID-19 diagnosis and treatment guide of the National Health Commission in China | Hefei and Fuyang |
| Tang HY 2020 | 2020.1.25-2020.3.20 | 225 | 112 | 113 | 72.82 | Retrospective cohort study | S | The COVID-19 diagnosis and treatment guide of the National Health Commission in China | Wuhan |
| Tao F 2020 | 2020.1.24-2020.3.1 | 382 | 190 | 192 | 52 | Retrospective cross-sectional study | S | The COVID-19 diagnosis and treatment guide of the National Health Commission in China | Wuhan |
| Tong XL 2021 | 2020.2.8-2020.3.15 | 198 | 99 | 99 | 63 | Retrospective cohort study | S | World Health Organization interim guidance | Wuhan |
| Wan SX 2020 | 2020.1.23-2020.2.8 | 135 | 72 | 63 | 47 | Retrospective cohort study | S | World Health Organization interim guidance | Chongqing |
| Wang B 2020 | 2019.12.30-2020.2.24 | 136 | 90 | 46 | 61.95 | Retrospective cohort study | S | The COVID-19 diagnosis and treatment guide of the National Health Commission in China | Wuhan |
| Wang D 2020 | 2020.1.15-2020.2.28 | 143 | 73 | 70 | 58 | Retrospective cross-sectional study | S | The COVID-19 diagnosis and treatment guide of the National Health Commission in China | Wuhan |
| Wang DW 2020a | -2020.2.10 | 107 | 57 | 50 | 51 | Retrospective cohort study | D | World Health Organization interim guidance | Wuhan and Xishui |
| Wang DW 2020b | 2020.1.1-2020.1.28 | 138 | 75 | 63 | 56 | Retrospective cohort study | S | World Health Organization interim guidance | Wuhan |
| Wang GZ 2021 | 2020.2.4-2020.3.29 | 2079 | 1085 | 994 | 58.46 | Retrospective cohort study | S | RT-PCR | Wuhan |
| Wang H 2020 | 2020.2.11-2020.4.30 | 113 | 74 | 39 | 64.94 | Retrospective cohort study | S | The COVID-19 diagnosis and treatment guide of the National Health Commission in China | Wuhan |
| Wang JH 2021 | 2020.2.9-2020.3.30 | 1135 | 545 | 590 | 62 | Retrospective cohort study | S | World Health Organization interim guidance | Wuhan |
| Wang K 2020 | 2020.1.7-2020.2.11 | 296 | 140 | 156 | 47.32 | Retrospective cohort study | S | The COVID-19 diagnosis and treatment guide of the National Health Commission in China | Wuhan |
| Wang LC 2020 | 2020.1.1-2020.2.29 | 235 | 131 | 104 | 70.6 | Retrospective cohort study | S | The COVID-19 diagnosis and treatment guide of the National Health Commission in China | Wuhan |
| Wang LL 2021 | 2020.1.15-2020.3.1 | 446 | 213 | 233 | 55 | Retrospective cohort study | M | The COVID-19 diagnosis and treatment guide of the National Health Commission in China | Wuhan |
| Wang LW 2020 | 2020.1.14-2020.2.13 | 116 | 67 | 49 | 54 | Retrospective cohort study | S | World Health Organization interim guidance | Wuhan |
| Wang M 2021 | 2019.12.21-2020.2.14 | 211 | 101 | 110 | 54 | Retrospective cohort study | S | World Health Organization interim guidance | Wuhan |
| Wang Q 2021 | 2019.12.15-2020.3.15 | 638 | 301 | 337 | 59 | Retrospective cohort study | S | The COVID-19 diagnosis and treatment guide of the National Health Commission in China | Wuhan |
| Wang QQ 2021 | 2020.2.6-2020.2.21 | 126 | 74 | 52 | 66 | Retrospective case control study | S | The COVID-19 diagnosis and treatment guide of the National Health Commission in China | Wuhan |
| Wang RR 2020 | 2020.1.30-2020.2.24 | 450 | 206 | 244 | 58 | Retrospective cohort study | S | RT-PCR | Wuhan |
| Wang T 2021 | 2020.1.27-2020.2.29 | 228 | 108 | 120 | 59 | Retrospective cohort study | S | The COVID-19 diagnosis and treatment guide of the National Health Commission in China | Wuhan |
| Wang W 2020 | 2020.2.9-2020.3.5 | 421 | 214 | 207 | 52 | Retrospective cohort study | S | World Health Organization interim guidance | Wuhan |
| Wang X 2020a | 2020.2.7-2020.2.12 | 1012 | 524 | 488 | 50 | Retrospective cohort study | S | RT-PCR | Wuhan |
| Wang X 2020b | 2020.1.10-2020.2.15 | 113 | 68 | 45 | 58.6 | Retrospective cohort study | S | The COVID-19 diagnosis and treatment guide of the National Health Commission in China | Wuhan |
| Wang Y 2020 | 2020.1.20-2020.2.13 | 125 | 57 | 68 | 47.5 | Retrospective cohort study | S | The COVID-19 diagnosis and treatment guide of the National Health Commission in China | Xiaogan |
| Wang Y 2021 | 2020.2.1-2020.3.16 | 317 | 169 | 148 | 59 | Retrospective cohort study | S | World Health Organization interim guidance | Wuhan |
| Wang YB 2020 | 2020.1.22-2020.2.15 | 272 | 126 | 146 | 48.6 | Retrospective cross-sectional study | S | The COVID-19 diagnosis and treatment guide of the National Health Commission in China | Guangzhou |
| Wang YF 2020 | 2020.1.1-2020.2.10 | 110 | 48 | 62 | ＞60 | Retrospective cohort study | S | World Health Organization interim guidance | Wuhan |
| Wang YP 2020 | 2020.1.20-2020.2.10 | 275 | 128 | 147 | 49 | Retrospective cohort study | S | World Health Organization interim guidance | Guangzhou |
| Wang ZG 2021 | 2020.1.29-2020.3.20 | 156 | 76 | 80 | 66 | Retrospective cohort study | S | The COVID-19 diagnosis and treatment guide of the National Health Commission in China | Wuhan |
| Wei YP 2020 | 2020.1.27-2020.3.11 | 276 | 155 | 121 | 51 | Retrospective cohort study | S | World Health Organization interim guidance | Suizhou |
| Wu GY 2020 | 2019.12.23-2020.2.13 | 299 | 137 | 162 | 47.51 | Retrospective cohort study | S | American Thoracic Society guidelines | Wuhan |
| Wu XD 2020 | 2020.1.1-2020.3.30 | 1091 | 509 | 582 | 59 | Retrospective cohort study | D | World Health Organization interim guidance | Wuhan |
| Xiao F 2021 | 2020.2月-2020.4月 | 316 | 164 | 152 | 64.5 | Retrospective cross-sectional study | S | The COVID-19 diagnosis and treatment guide of the National Health Commission in China | Wuhan |
| Xie D 2021 | 2020.2-2020.3 | 118 | 66 | 52 | 58.6 | Retrospective cohort study | S | The COVID-19 diagnosis and treatment guide of the National Health Commission in China | Wuhan |
| Xie JJ 2020 | 2020.1.10-2020.2.26 | 104 | 63 | 41 | 55 | Retrospective cohort study | S | World Health Organization interim guidance | Zhejiang |
| Xie XY 2020 | 2020.2-2020.4 | 216 | 117 | 99 | 59.42 | Retrospective cohort study | S | The COVID-19 diagnosis and treatment guide of the National Health Commission in China | Wuhan |
| Xiong P 2020 | 2020.1.2-2020.2.15 | 660 | 295 | 365 | 56 | Retrospective cohort study | S | The COVID-19 diagnosis and treatment guide of the National Health Commission in China | Wuhan |
| Xiong SQ 2020 | 2020.1.20-2020.3.8 | 116 | 80 | 36 | 58.5 | Retrospective cohort study | S | World Health Organization interim guidance | Wuhan |
| Xiong W 2021 | 2020.1-2020.2 | 123 | 61 | 62 | 57.7 | Retrospective cohort study | S | The COVID-19 diagnosis and treatment guide of the National Health Commission in China | Wuhan |
| Xiong YB 2021 | 2020.1.24-2020.3.31 | 298 | 166 | 132 | 60 | Retrospective cohort study | S | The COVID-19 diagnosis and treatment guide of the National Health Commission in China | Wuhan |
| Xu B 2020 | 2019.12.26-2020.3.1 | 187 | 103 | 84 | 62 | Retrospective cohort study | S | World Health Organization interim guidance | Wuhan |
| Xu H 2021a | 2020.1.20-2020.2.18 | 221 | 133 | 88 | 45.95 | Retrospective cohort study | S | The COVID-19 diagnosis and treatment guide of the National Health Commission in China | Xianyang |
| Xu H 2021b | 2020.2-2020.4 | 122 | 80 | 42 | 70.1 | Retrospective cohort study | S | The COVID-19 diagnosis and treatment guide of the National Health Commission in China | Wuhan |
| Xu JJ 2021 | 2019.12-2020.3 | 338 | 146 | 192 | 57.5 | Retrospective cohort study | D | World Health Organization interim guidance | Wuhan |
| Xu JQ 2020 | 2020.1.12-2020.2.3 | 239 | 143 | 96 | 62.5 | Retrospective cohort study | M | The COVID-19 diagnosis and treatment guide of the National Health Commission in China | Wuhan |
| Xu W 2021 | 2020.1.20-2020.10.20 | 1003 | 602 | 401 | 36 | Retrospective cohort study | S | The COVID-19 diagnosis and treatment guide of the National Health Commission in China | Shanghai |
| Yan XQ 2020 | 2020.1.21-2020.6.27 | 194 | 106 | 88 | 44.259 | Retrospective cohort study | M | World Health Organization interim guidance | Loudi, Shaoyang, and Xiangtan |
| Yang LH 2020 | 2020.1.30-2020.2.8 | 200 | 98 | 102 | 55 | Retrospective cohort study | S | World Health Organization interim guidance | Yichang |
| Yang M 2020 | 2020.1.17-2020.3.12 | 108 | 42 | 66 | 51 | Retrospective cohort study | S | The COVID-19 diagnosis and treatment guide of the National Health Commission in China | Wuhan |
| Yang Q 2020 | 2020.1.1-2020.2.29 | 226 | 113 | 113 | 53.89 | Retrospective cohort study | S | World Health Organization interim guidance | Wuhan |
| Yang QX 2020 | 2020.1.28-2020.2.12 | 136 | 66 | 70 | 56 | Retrospective cohort study | S | The COVID-19 diagnosis and treatment guide of the National Health Commission in China | Wuhan |
| Yang QX 2021 | 2020.2.10-2020.2.16 | 142 | 69 | 73 | 68 | Retrospective cohort study | S | The COVID-19 diagnosis and treatment guide of the National Health Commission in China | Wuhan |
| Yang XH 2020 | 2020.2.12-2020.3.7 | 412 | 183 | 229 | 60.2 | Retrospective cohort study | S | The COVID-19 diagnosis and treatment guide of the National Health Commission in China | Wuhan |
| Yi CF 2020 | 2020.1.20-2020.3.20 | 120 | 59 | 61 | 62.5 | Retrospective cohort study | S | The COVID-19 diagnosis and treatment guide of the National Health Commission in China | Wuhan |
| Yi P 2020 | 2020.1.19-2020.2.19 | 100 | 63 | 37 | 54 | Retrospective cohort study | S | The COVID-19 diagnosis and treatment guide of the National Health Commission in China | Zhejiang |
| Yin ZW 2021 | 2020.1.20-2020.3.20 | 474 | 247 | 227 | 60±20 | Retrospective cross-sectional study | S | The COVID-19 diagnosis and treatment guide of the National Health Commission in China | Wuhan |
| Yu CZ 2020a | 2020.1.14-2020.2.28 | 1464 | 736 | 728 | 64 | Retrospective cohort study | S | The COVID-19 diagnosis and treatment guide of the National Health Commission in China | Wuhan |
| Yu CZ 2020b | 2020.1.14-2020.2.28 | 1663 | 838 | 825 | 64 | Retrospective cohort study | S | The COVID-19 diagnosis and treatment guide of the National Health Commission in China | Wuhan |
| Yu HZ 2020 | 2020.1.21-2020.3.15 | 135 | 73 | 62 | 48.6 | Retrospective cohort study | S | The COVID-19 diagnosis and treatment guide of the National Health Commission in China | Tianjin |
| Yu XM 2021 | 2020.2.1-2020.2.5 | 151 | 67 | 84 | 58 | Retrospective cohort study | S | The COVID-19 diagnosis and treatment guide of the National Health Commission in China | Wuhan |
| Yuan J 2020 | 2020.1.24-2020.2.23 | 223 | 106 | 117 | 46.5 | Retrospective cohort study | S | The COVID-19 diagnosis and treatment guide of the National Health Commission in China | Chongqing |
| Yuan LY 2021 | 2020.1.30-2020.2.15 | 264 | 162 | 102 | 64.86 | Retrospective cohort study | S | The COVID-19 diagnosis and treatment guide of the National Health Commission in China | Wuhan |
| Yuan XF 2021 | 2020.1.17-2020.3.14 | 169 | 81 | 88 | 45 | Retrospective cohort study | S | The COVID-19 diagnosis and treatment guide of the National Health Commission in China | Changsha |
| Zeng HL 2021a | 2020.2.10-2020.3.15 | 306 | 148 | 158 | 63 | Retrospective cohort study | S | The COVID-19 diagnosis and treatment guide of the National Health Commission in China | Wuhan |
| Zeng HL 2021b | 2020.1.17-2020.2.14 | 642 | 318 | 324 | 63.5 | Retrospective cohort study | S | The COVID-19 diagnosis and treatment guide of the National Health Commission in China | Wuhan |
| Zeng JH 2020 | 2020.1.11-2020.4.1 | 416 | 198 | 218 | 46.59 | Retrospective cohort study | S | World Health Organization interim guidance | Shenzhen |
| Zeng ZH 2021 | 2020.1.22-2020.3.14 | 461 | 239 | 222 | 45 | Retrospective cohort study | M | World Health Organization interim guidance | Changsha, Zhuzhou, Xiangtan, Yueyang, and Shaoyang |
| Zhang GQ 2020 | 2020.1.2-2020.2.10 | 221 | 108 | 113 | 55 | Retrospective cohort study | S | World Health Organization interim guidance | Wuhan |
| Zhang J 2020a | 2020.1.13-2020.2.16 | 111 | 46 | 65 | 38 | Retrospective cohort study | S | World Health Organization interim guidance | Wuhan |
| Zhang J 2020b | 2020.1.11-2020.2.6 | 663 | 321 | 342 | 55.6 | Retrospective cohort study | S | World Health Organization interim guidance and The COVID-19 diagnosis and treatment guide of the National Health Commission in China | Wuhan |
| Zhang J 2020c | 2020.2.5-2020. | 901 | 435 | 466 | 60 | Retrospective cohort study | S | The COVID-19 diagnosis and treatment guide of the National Health Commission in China | Wuhan |
| Zhang J 2021 | 2020.1.3-2020.3.30 | 208 | 129 | 79 | 64 | Retrospective case control study | S | The COVID-19 diagnosis and treatment guide of the National Health Commission in China | Wuhan |
| Zhang JH 2021 | 2020.2.1-2020.3.15 | 135 | 67 | 68 | 56 | Retrospective cohort study | S | The COVID-19 diagnosis and treatment guide of the National Health Commission in China | Wuhan |
| Zhang JJ 2020 | 2020.1.16-2020.2.3 | 140 | 71 | 69 | 57 | Retrospective cohort study | S | The COVID-19 diagnosis and treatment guide of the National Health Commission in China | Wuhan |
| Zhang L 2020 | 2020.1.27-2020.3.5 | 386 | 192 | 194 | 59.58 | Retrospective cohort study | S | The COVID-19 diagnosis and treatment guide of the National Health Commission in China | Wuhan |
| Zhang LH 2020 | 2020.1.16-2020.2.16 | 101 | 52 | 49 | 49.81 | Retrospective cohort study | S | The COVID-19 diagnosis and treatment guide of the National Health Commission in China | Chengdu |
| Zhang LY 2021 | 2020.1.28-2020.3.10 | 478 | 267 | 211 | 46.8 | Retrospective cross-sectional study | S | The COVID-19 diagnosis and treatment guide of the National Health Commission in China | Wuhan |
| Zhang QQ 2021 | 2020.1-2020.2 | 172 | 92 | 80 | 47.9 | Retrospective cohort study | M | World Health Organization interim guidance | Henan, Zhoukou, Nanyang, Nanyang, and Dengzhou |
| Zhang QX 2021 | 2020.2.7-2020.3.28 | 319 | 173 | 146 | 51.6 | Retrospective cohort study | S | The COVID-19 diagnosis and treatment guide of the National Health Commission in China | Yichang |
| Zhang R 2020 | 2020.1.1-2020.2.10 | 120 | 43 | 77 | 45.4 | Retrospective cohort study | S | World Health Organization interim guidance | Wuhan |
| Zhang SQ 2020 | 2020.2.15-2020.3.31 | 134 | 69 | 65 | 33 | Retrospective cohort study | S | The COVID-19 diagnosis and treatment guide of the National Health Commission in China | Guizhou |
| Zhang W 2021 | 2020.2.20-2020.4.4 | 547 | 285 | 262 | 58.34 | Retrospective cohort study | S | The COVID-19 diagnosis and treatment guide of the National Health Commission in China | Wuhan |
| Zhang XB 2021 | 2020.1.20-2020.3.30 | 432 | 230 | 202 | 54 | Retrospective cohort study | D | The COVID-19 diagnosis and treatment guide of the National Health Commission in China | Wuhan |
| Zhang YP 2020 | 2020.1.1-2020.3.18 | 365 | 176 | 189 | 52.6 | Retrospective cohort study | D | The COVID-19 diagnosis and treatment guide of the National Health Commission in China | Honghu and Nanchang |
| Zhao C 2021 | 2020.2.13-2020.2.25 | 172 | 82 | 90 | 65 | Retrospective cohort study | S | World Health Organization interim guidance | Wuhan |
| Zhao CC 2020 | 2020.1.24-2020.2.17 | 189 | 91 | 98 | 46.5 | Retrospective cross-sectional study | S | The COVID-19 diagnosis and treatment guide of the National Health Commission in China | Bengbu and Fuyang |
| Zheng YQ 2020 | 2020.01-2020.03 | 224 | 134 | 90 | 52.5 | Retrospective cross-sectional study | S | The COVID-19 diagnosis and treatment guide of the National Health Commission in China | Wuhan |
| Zhong JN 2020 | 2020.1.1-2020.2.20 | 764 | 365 | 399 | 59.33 | Retrospective cohort study | S | The COVID-19 diagnosis and treatment guide of the National Health Commission in China | Wuhan |
| Zhou C 2021 | 2020.1.27-2020.3.18 | 511 | 247 | 264 | 60.8 | Retrospective cohort study | S | The COVID-19 diagnosis and treatment guide of the National Health Commission in China | Wuhan |
| Zhou F 2020 | 2019.12.29-2020.1.31 | 191 | 119 | 72 | 56 | Retrospective cohort study | D | World Health Organization interim guidance | Wuhan |
| Zhou J 2020 | 2020.1.12-2020.2.26 | 118 | 53 | 65 | 71.68 | Retrospective cohort study | S | World Health Organization interim guidance | Wuhan |
| Zhou QL 2021 | 2020.1.21-2020.3.11 | 248 | 134 | 114 | 47.69 | Retrospective cohort study | S | The COVID-19 diagnosis and treatment guide of the National Health Commission in China | Chongqing |
| Zhou SL 2021 | 2020.1.11-2020.2.18 | 220 | 104 | 116 | 59.5 | Retrospective cohort study | S | The COVID-19 diagnosis and treatment guide of the National Health Commission in China | Wuhan |
| Zou L 2020 | 2020.1.16-2020.3.3 | 121 | 66 | 55 | 65 | Retrospective cross-sectional study | S | The COVID-19 diagnosis and treatment guide of the National Health Commission in China | Wuhan |

M: Multi center; Single center: S.

**References of included studies**

1. An, Y. W., Song, S., Li, W. X., Chen, Y. X., Hu, X. P., Zhao, J., et al. Liver function recovery of COVID-19 patients after discharge, a follow-up study. International journal of medical sciences. (2021) 18, 176-186. doi:10.7150/ijms.50691.

2. Cai, Q., Chen, F., Wang, T., Luo, F., Liu, X., Wu, Q., et al. Obesity and COVID-19 Severity in a Designated Hospital in Shenzhen, China. Diabetes care. (2020) 43, 1392-1398. doi:10.2337/dc20-0576.

3. Cai, Q., Huang, D., Ou, P., Yu, H., Zhu, Z., Xia, Z., et al. COVID-19 in a designated infectious diseases hospital outside Hubei Province, China. Allergy. (2020) 75, 1742-1752. doi:10.1111/all.14309.

4. Caillon, A., Zhao, K., Klein, K. O., Greenwood, C. M. T., Lu, Z., Paradis, P., et al. High Systolic Blood Pressure at Hospital Admission Is an Important Risk Factor in Models Predicting Outcome of COVID-19 Patients. American journal of hypertension. (2021) 34, 282-290. doi:10.1093/ajh/hpaa225.

5. Cao, J., Tu, W. J., Cheng, W., Yu, L., Liu, Y. K., Hu, X., et al. Clinical Features and Short-term Outcomes of 102 Patients with Coronavirus Disease 2019 in Wuhan, China. Clinical infectious diseases : an official publication of the Infectious Diseases Society of America. (2020) 71, 748-755. doi:10.1093/cid/ciaa243.

6. Cao, M., Zhang, D., Wang, Y., Lu, Y., Zhu, X., Li, Y., et al. Clinical Features of Patients Infected with the 2019 Novel Coronavirus (COVID-19) in Shanghai, China. medRxiv : the preprint server for health sciences. (2020). doi:10.1101/2020.03.04.20030395.

7. Cao, Y., Han, X., Gu, J., Li, Y., Liu, J., Alwalid, O., et al. Prognostic value of baseline clinical and HRCT findings in 101 patients with severe COVID-19 in Wuhan, China. Scientific reports. (2020) 10, 17543. doi:10.1038/s41598-020-74497-9.

8. Chen, F. F., Zhong, M., Liu, Y., Zhang, Y., Zhang, K., Su, D. Z., et al. The characteristics and outcomes of 681 severe cases with COVID-19 in China. Journal of critical care. (2020) 60, 32-37. doi:10.1016/j.jcrc.2020.07.003.

9. Chen, H., Li, X., Marmar, T., Xu, Q., Tu, J., Li, T., et al. Cardiac Troponin I association with critical illness and death risk in 726 seriously ill COVID-19 patients: A retrospective cohort study. International journal of medical sciences. (2021) 18, 1474-1483. doi:10.7150/ijms.53641.

10. Chen, L., Chen, J., Liu, Y. W., Du, X. B., Peng, L., Jin, Y. L., et al. Risk Factors and Predictive Scoring System Development for COVID-19 Death. Chinese General Practice. (2020) 23, 4419-4424.

11. Chen, L., Liu, S., Tian, J., Pan, H., Liu, Y., Hu, J., et al. Disease progression patterns and risk factors associated with mortality in deceased patients with COVID-19 in Hubei Province, China. Immunity, inflammation and disease. (2020) 8, 584-594. doi:10.1002/iid3.343.

12. Chen, Q., Zheng, Z., Zhang, C., Zhang, X., Wu, H., Wang, J., et al. Clinical characteristics of 145 patients with corona virus disease 2019 (COVID-19) in Taizhou, Zhejiang, China. Infection. (2020) 48, 543-551. doi:10.1007/s15010-020-01432-5.

13. Chen, T., Wu, D., Chen, H., Yan, W., Yang, D., Chen, G., et al. Clinical characteristics of 113 deceased patients with coronavirus disease 2019: retrospective study. BMJ (Clinical research ed). (2020) 368, m1091. doi:10.1136/bmj.m1091.

14. Chen, Y., Ke, Y., Liu, X., Wang, Z., Jia, R., Liu, W., et al. Clinical features and antibody response of patients from a COVID-19 treatment hospital in Wuhan, China. Journal of medical virology. (2021) 93, 2782-2789. doi:10.1002/jmv.26617.

15. Chen, Y., Linli, Z., Lei, Y., Yang, Y., Liu, Z., Xia, Y., et al. Risk factors for mortality in critically ill patients with COVID-19 in Huanggang, China: A single-center multivariate pattern analysis. Journal of medical virology. (2021) 93, 2046-2055. doi:10.1002/jmv.26572.

16. Cheng, B., Hu, J., Zuo, X., Chen, J., Li, X., Chen, Y., et al. Predictors of progression from moderate to severe coronavirus disease 2019: a retrospective cohort. Clinical microbiology and infection : the official publication of the European Society of Clinical Microbiology and Infectious Diseases. (2020) 26, 1400-1405. doi:10.1016/j.cmi.2020.06.033.

17. Cheng, K., Xiong, W., Zhou, X., Li, H., Zheng, J., Xu, J. Diagnosis and treatment of 471 patients with 2019 novel coronavirus disease (COVID-19). Annals of translational medicine. (2021) 9, 163. doi:10.21037/atm-21-236.

18. Deng, Q., Hu, B., Zhang, Y., Wang, H., Zhou, X., Hu, W., et al. Suspected myocardial injury in patients with COVID-19: Evidence from front-line clinical observation in Wuhan, China. International journal of cardiology. (2020) 311, 116-121. doi:10.1016/j.ijcard.2020.03.087.

19. Deng, Y., Liu, W., Liu, K., Fang, Y. Y., Shang, J., Zhou, L., et al. Clinical characteristics of fatal and recovered cases of coronavirus disease 2019 in Wuhan, China: a retrospective study. Chinese medical journal. (2020) 133, 1261-1267. doi:10.1097/cm9.0000000000000824.

20. Ding, R., Yang, Z., Huang, D., Wang, Y., Li, X., Zhou, X., et al. Identification of parameters in routine blood and coagulation tests related to the severity of COVID-19. International journal of medical sciences. (2021) 18, 1207-1215. doi:10.7150/ijms.47494.

21. Fan, H., Cai, J., Tian, A., Li, Y., Yuan, H., Jiang, Z., et al. Comparison of Liver Biomarkers in 288 COVID-19 Patients: A Mono-Centric Study in the Early Phase of Pandemic. Frontiers in medicine. (2021) 7, 584888. doi:10.3389/fmed.2020.584888.

22. Fang, X., Cai, J., Liang, Q., Li, X., Bian, Y. Clinical characteristics and chest CT findings in moderate and severe COVID-19 patients: an analysis of 506 cases from Wuhan Huoshenshan Hospital. Chinese Journal of Clinical Infectious Diseases. (2020) 13, 161-166.

23. Feng, X., Li, P., Ma, L., Liang, H., Lei, J., Li, W., et al. Clinical Characteristics and Short-Term Outcomes of Severe Patients With COVID-19 in Wuhan, China. Frontiers in medicine. (2020) 7, 491. doi:10.3389/fmed.2020.00491.

24. Feng, Y., Ling, Y., Bai, T., Xie, Y., Huang, J., Li, J., et al. COVID-19 with Different Severities: A Multicenter Study of Clinical Features. American journal of respiratory and critical care medicine. (2020) 201, 1380-1388. doi:10.1164/rccm.202002-0445OC.

25. Gao, S., Jiang, F., Jin, W., Shi, Y., Yang, L., Xia, Y., et al. Risk factors influencing the prognosis of elderly patients infected with COVID-19: a clinical retrospective study in Wuhan, China. Aging. (2020) 12, 12504-12516. doi:10.18632/aging.103631.

26. Guo, X., Jie, Y., Ye, Y., Chen, P., Li, X., Gao, Z., et al. Upper Respiratory Tract Viral Ribonucleic Acid Load at Hospital Admission Is Associated With Coronavirus Disease 2019 Disease Severity. Open forum infectious diseases. (2020) 7, ofaa282. doi:10.1093/ofid/ofaa282.

27. Hu, L., Chen, S., Fu, Y., Gao, Z., Long, H., Ren, H. W., et al. Risk Factors Associated With Clinical Outcomes in 323 Coronavirus Disease 2019 (COVID-19) Hospitalized Patients in Wuhan, China. Clinical infectious diseases : an official publication of the Infectious Diseases Society of America. (2020) 71, 2089-2098. doi:10.1093/cid/ciaa539.

28. Hu, Q., Tang, Y. W., Song, Y. Y., Wang, J., Liu, X. S., Yang, H. Y., et al. Analysis on clinical characteristics of patients with COVID-19 complicating renal function injury in Chongqing City. Chongqing Medicine. (2021) 50, 2222-2226+2232.

29. Hu, Q., Xu, Y., Xiang, Y., Wang, B., Yuan, Z., Shan, Y., et al. Inflammation characteristics and anti-inflammation treatment with tocilizumab of severe/critical COVID-19 patients: A retrospective cohort study. International journal of biological sciences. (2021) 17, 2124-2134. doi:10.7150/ijbs.56952.

30. Hu, X., Hu, C., Yang, Y., Chen, J., Zhong, P., Wen, Y., et al. Clinical characteristics and risk factors for severity of COVID-19 outside Wuhan: a double-center retrospective cohort study of 213 cases in Hunan, China. Therapeutic advances in respiratory disease. (2020) 14, 1753466620963035. doi:10.1177/1753466620963035.

31. Huang, R., Zhu, L., Xue, L., Liu, L., Yan, X., Wang, J., et al. Clinical findings of patients with coronavirus disease 2019 in Jiangsu province, China: A retrospective, multi-center study. PLoS neglected tropical diseases. (2020) 14, e0008280. doi:10.1371/journal.pntd.0008280.

32. Huang, Y., Cai, C., Zang, J., Xie, J., Xu, D., Zheng, F., et al. Treatment strategies of hospitalized patients with coronavirus disease-19. Aging. (2020) 12, 11224-11237. doi:10.18632/aging.103370.

33. Jiang, Y., Abudurexiti, S., An, M. M., Cao, D., Wei, J., Gong, P. Risk factors associated with 28-day all-cause mortality in older severe COVID-19 patients in Wuhan, China: a retrospective observational study. Scientific reports. (2020) 10, 22369. doi:10.1038/s41598-020-79508-3.

34. Jiao, L., Huang, X. R., Zhang, W., Fan, Q., Cheng, W. L., Zhao, F. Analysis of myocardial injury markers and electrocardiogram in 126 COVID-19 cases. Chinese Journal of Cardiovascular Research. (2020) 18, 484-487+537.

35. Li, H., Ren, L., Zhang, L., Wang, Y., Guo, L., Wang, C., et al. High anal swab viral load predisposes adverse clinical outcomes in severe COVID-19 patients. Emerging microbes & infections. (2020) 9, 2707-2714. doi:10.1080/22221751.2020.1858700.

36. Li, H. Y., Wang, J. W., Xu, L. W., Zhao, X. L., Feng, J. X., Xu, Y. Z. Clinical analysis of 132 cases COVID-19 from Wuhan. Medicine. (2020) 99, e22847. doi:10.1097/md.0000000000022847.

37. Li, J., Luo, H., Deng, G., Chang, J., Qiu, X., Liu, C., et al. Multidimensional Evaluation of All-Cause Mortality Risk and Survival Analysis for Hospitalized Patients with COVID-19. International journal of medical sciences. (2021) 18, 3140-3149. doi:10.7150/ijms.58889.

38. Li, J., Yang, L., Zeng, Q., Li, Q., Yang, Z., Han, L., et al. Determinants of mortality of patients with COVID-19 in Wuhan, China: a case-control study. Annals of palliative medicine. (2021) 10, 3937-3950. doi:10.21037/apm-20-2107.

39. Li, L. Z., He, B., Zhang, S. D., Wang, S. H., Zhang, M. X., Zhao, Q. Y. Association of myocardial damage with death in critical coronavirus disease 2019. The Journal of Practical Medicine. (2021) 37, 6-10.

40. Li, M., Cheng, B., Zeng, W., Chen, S., Tu, M., Wu, M., et al. Analysis of the Risk Factors for Mortality in Adult COVID-19 Patients in Wuhan: A Multicenter Study. Frontiers in medicine. (2020) 7, 545. doi:10.3389/fmed.2020.00545.

41. Li, N., Kong, H., Zheng, X. Z., Li, X. Y., Ma, J., Zhang, H., et al. Early predictive factors of progression from severe type to critical ill type in patients with Coronavirus Disease 2019: A retrospective cohort study. PloS one. (2020) 15, e0243195. doi:10.1371/journal.pone.0243195.

42. Li, P., Wu, W., Zhang, T., Wang, Z., Li, J., Zhu, M., et al. Implications of cardiac markers in risk-stratification and management for COVID-19 patients. Critical care (London, England). (2021) 25, 158. doi:10.1186/s13054-021-03555-z.

43. Li, Q., Cao, Y., Chen, L., Wu, D., Yu, J., Wang, H., et al. Hematological features of persons with COVID-19. Leukemia. (2020) 34, 2163-2172. doi:10.1038/s41375-020-0910-1.

44. Li, R. Q., Tao, J., Yao, X. H., Yang, F., Peng, P., Tang, J., et al. Multi-Center Clinical Research of Risk Factors Associated with Severe and Critical Patients with Coronavirus Disease 2019. China Pharmaceuticals. (2020) 29, 15-18.

45. Li, T., Lu, L., Zhang, W., Tao, Y., Wang, L., Bao, J., et al. Clinical characteristics of 312 hospitalized older patients with COVID-19 in Wuhan, China. Archives of gerontology and geriatrics. (2020) 91, 104185. doi:10.1016/j.archger.2020.104185.

46. Li, W. X., Xu, W., Huang, C. L., Fei, L., Xie, X. D., Li, Q., et al. Acute cardiac injury and acute kidney injury associated with severity and mortality in patients with COVID-19. European review for medical and pharmacological sciences. (2021) 25, 2114-2122. doi:10.26355/eurrev_202102_25117.

47. Li, X., Xu, S., Yu, M., Wang, K., Tao, Y., Zhou, Y., et al. Risk factors for severity and mortality in adult COVID-19 inpatients in Wuhan. The Journal of allergy and clinical immunology. (2020) 146, 110-118. doi:10.1016/j.jaci.2020.04.006.

48. Li, X. Q., Liu, H., Meng, Y., Yin, H. Y., Gao, W. Y., Yang, X., et al. Critical roles of cytokine storm and secondary bacterial infection in acute kidney injury development in COVID-19: A multi-center retrospective cohort study. Journal of medical virology. (2021) 93, 6641-6652. doi:10.1002/jmv.27234.

49. Li, Y., Li, H., Song, C., Lu, R., Zhao, Y., Lin, F., et al. Early Prediction of Disease Progression in Patients with Severe COVID-19 Using C-Reactive Protein to Albumin Ratio. Disease markers. (2021) 2021, 6304189. doi:10.1155/2021/6304189.

50. Li, Y., Liu, T., Tse, G., Wu, M., Jiang, J., Liu, M., et al. Electrocardiograhic characteristics in patients with coronavirus infection: A single-center observational study. Annals of noninvasive electrocardiology : the official journal of the International Society for Holter and Noninvasive Electrocardiology, Inc. (2020) 25, e12805. doi:10.1111/anec.12805.

51. Li, Y., Zhang, Y., Lu, R., Dai, M., Shen, M., Zhang, J., et al. Lipid metabolism changes in patients with severe COVID-19. Clinica chimica acta; international journal of clinical chemistry. (2021) 517, 66-73. doi:10.1016/j.cca.2021.02.011.

52. Liao, D., Zhou, F., Luo, L., Xu, M., Wang, H., Xia, J., et al. Haematological characteristics and risk factors in the classification and prognosis evaluation of COVID-19: a retrospective cohort study. The Lancet Haematology. (2020) 7, e671-e678. doi:10.1016/s2352-3026(20)30217-9.

53. Lin, H. Y., Qin, X. J. Analysis of clinical characteristics of 1767 cases of novel coronavirus pneumonia. Practical Journal of Medicine & Pharmacy. (2021) 38, 537-539. doi:10.14172/j.issn1671-4008.2021.06.017.

54. Liu, D., Cui, P., Zeng, S., Wang, S., Feng, X., Xu, S., et al. Risk factors for developing into critical COVID-19 patients in Wuhan, China: A multicenter, retrospective, cohort study. EClinicalMedicine. (2020) 25, 100471. doi:10.1016/j.eclinm.2020.100471.

55. Liu, F. Y., Sun, X. L., Zhang, Y., Ge, L., Wang, J., Liang, X., et al. Evaluation of the Risk Prediction Tools for Patients With Coronavirus Disease 2019 in Wuhan, China: A Single-Centered, Retrospective, Observational Study. Critical care medicine. (2020) 48, e1004-e1011. doi:10.1097/ccm.0000000000004549.

56. Liu, J., Liu, Z., Jiang, W., Wang, J., Zhu, M., Song, J., et al. Clinical predictors of COVID-19 disease progression and death: Analysis of 214 hospitalised patients from Wuhan, China. The clinical respiratory journal. (2021) 15, 293-309. doi:10.1111/crj.13296.

57. Liu, J., Tu, C., Zhu, M., Wang, J., Yang, C., Liu, W., et al. The clinical course and prognostic factors of severe COVID-19 in Wuhan, China: A retrospective case-control study. Medicine. (2021) 100, e23996. doi:10.1097/md.0000000000023996.

58. Liu, J., Zhang, S., Wu, Z., Shang, Y., Dong, X., Li, G., et al. Clinical outcomes of COVID-19 in Wuhan, China: a large cohort study. Annals of intensive care. (2020) 10, 99. doi:10.1186/s13613-020-00706-3.

59. Liu, L., Hu, K. Y., Shao, X. N., Liang, Y., Lei, M. Clinical analysis of novel coronavirus pneumonia complicated with acute kidney injury. The Journal of Practical Medicine. (2020) 36, 835-838.

60. Liu, M., Han, S., Liao, Q., Chang, L., Tan, Y., Jia, P., et al. Outcomes and prognostic factors in 70 non-survivors and 595 survivors with COVID-19 in Wuhan, China. Transboundary and emerging diseases. (2021) 68, 3611-3623. doi:10.1111/tbed.13969.

61. Liu, Q. Q., Cheng, A., Wang, Y., Li, H., Hu, L., Zhao, X., et al. Cytokines and their relationship with the severity and prognosis of coronavirus disease 2019 (COVID-19): a retrospective cohort study. BMJ open. (2020) 10, e041471. doi:10.1136/bmjopen-2020-041471.

62. Liu, S. J., Cheng, F., Wu, Y. Y., Li, Z. N., Gong, G. F., Duan, X. Q., et al. Analysis of laboratory indexes and risk factors of patients with severe Corona Virus Disease 2019. Laboratory Medicine. (2020) 35, 1229-1233.

63. Liu, X. Q., Xue, S., Xu, J. B., Ge, H., Mao, Q., Xu, X. H., et al. Clinical characteristics and related risk factors of disease severity in 101 COVID-19 patients hospitalized in Wuhan, China. Acta pharmacologica Sinica. (2022) 43, 64-75. doi:10.1038/s41401-021-00627-2.

64. Liu, Z., Zhang, L., Zhan, L. Y., Xia, W. F., Chen, W., Wang, C. Y., et al. Clinical Characteristics and Predictors Risk Factors in Hospitalized Severe/Critical COVID-19 Patients. Chinese Journal of Microcirculation. (2021) 31, 46-51+56.

65. Liu, Z. Z., Cheng, D. R., Xu, X. L., Wu, Y. J., Pan, Y. H., Liu, J., et al. Kidney injury in patients with COVID-19 the association with outcome. Chinese Journal of Nephrology,Dialysis & Transplantation. (2020) 29, 401-407.

66. Long, X., Zhang, Z., Zou, W., Ling, J., Li, D., Jing, L., et al. Coagulopathy of Patients with COVID-19 is Associated with Infectious and Inflammatory Markers. Risk management and healthcare policy. (2020) 13, 1965-1975. doi:10.2147/rmhp.s268238.

67. Lu, Z. L., He, R. Y., Jiang, W. X., Fan, T., Geng, Q. Clinical characteristics and immune function analysis of COVID-19. Medical Journal of Wuhan University. (2020) 41, 529-532+546. doi:10.14188/j.1671-8852.2020.0126.

68. Luo, M., Liu, J., Jiang, W., Yue, S., Liu, H., Wei, S. IL-6 and CD8+ T cell counts combined are an early predictor of in-hospital mortality of patients with COVID-19. JCI insight. (2020) 5. doi:10.1172/jci.insight.139024.

69. Lv, Z., Cheng, S., Le, J., Huang, J., Feng, L., Zhang, B., et al. Clinical characteristics and co-infections of 354 hospitalized patients with COVID-19 in Wuhan, China: a retrospective cohort study. Microbes and infection. (2020) 22, 195-199. doi:10.1016/j.micinf.2020.05.007.

70. Mi, J., Zhong, W., Huang, C., Zhang, W., Tan, L., Ding, L. Gender, age and comorbidities as the main prognostic factors in patients with COVID-19 pneumonia. American journal of translational research. (2020) 12, 6537-6548.

71. Mo, J., Liu, J., Wu, S., LÜ, A., Xiao, L., Chen, D., et al. Predictive role of clinical features in patients with coronavirus disease 2019 for severe disease. Zhong nan da xue xue bao Yi xue ban = Journal of Central South University Medical sciences. (2020) 45, 536-541. doi:10.11817/j.issn.1672-7347.2020.200384.

72. Pan, D., Cheng, D., Cao, Y., Hu, C., Zou, F., Yu, W., et al. A Predicting Nomogram for Mortality in Patients With COVID-19. Frontiers in public health. (2020) 8, 461. doi:10.3389/fpubh.2020.00461.

73. Pei, G., Zhang, Z., Peng, J., Liu, L., Zhang, C., Yu, C., et al. Renal Involvement and Early Prognosis in Patients with COVID-19 Pneumonia. Journal of the American Society of Nephrology : JASN. (2020) 31, 1157-1165. doi:10.1681/asn.2020030276.

74. Peng, F., Lei, S., Zhang, Q., Zhong, Y., Wu, S. Smoking Is Correlated With the Prognosis of Coronavirus Disease 2019 (COVID-19) Patients: An Observational Study. Frontiers in physiology. (2021) 12, 634842. doi:10.3389/fphys.2021.634842.

75. Peng, L., Lv, Q. Q., Yang, F., Wu, X. M., Zhang, C. C., Wang, Y. Q., et al. The interval between onset and admission predicts disease progression in COVID-19 patients. Annals of translational medicine. (2021) 9, 213. doi:10.21037/atm-20-5320.

76. Peng, S., He, L., Wu, X. Y., Li, L. Predictive value of D-dimer in assessment of severity of patients with COVID-19 and its prognosis. The Journal of Practical Medicine. (2020) 36, 2321-2324.

77. Qi, X., Kong, H., Ding, W., Wu, C., Ji, N., Huang, M., et al. Abnormal Coagulation Function of Patients With COVID-19 Is Significantly Related to Hypocalcemia and Severe Inflammation. Frontiers in medicine. (2021) 8, 638194. doi:10.3389/fmed.2021.638194.

78. Qin, S., Li, W., Shi, X., Wu, Y., Wang, C., Shen, J., et al. 3044 Cases reveal important prognosis signatures of COVID-19 patients. Computational and structural biotechnology journal. (2021) 19, 1163-1175. doi:10.1016/j.csbj.2021.01.042.

79. Qin, Z. J., Liu, L., Sun, Q., Li, X., Luo, J. F., Liu, J. S., et al. Impaired immune and coagulation systems may be early risk factors for COVID-19 patients: A retrospective study of 118 inpatients from Wuhan, China. Medicine. (2020) 99, e21700. doi:10.1097/md.0000000000021700.

80. Qu, J., Chang, L. K., Tang, X., Du, Y., Yang, X., Liu, X., et al. Clinical characteristics of COVID-19 and its comparison with influenza pneumonia. Acta clinica Belgica. (2020) 75, 348-356. doi:10.1080/17843286.2020.1798668.

81. Rao, X., Wu, C., Wang, S., Tong, S., Wang, G., Wu, G., et al. The importance of overweight in COVID-19: A retrospective analysis in a single center of Wuhan, China. Medicine. (2020) 99, e22766. doi:10.1097/md.0000000000022766.

82. Ren, L., Yao, D., Cui, Z., Chen, S., Yan, H. Corona Virus Disease 2019 patients with different disease severity or age range: A single-center study of clinical features and prognosis. Medicine. (2020) 99, e22899. doi:10.1097/md.0000000000022899.

83. Ren, M. X., Li, T. Z., Zhang, J. Y., Duan, Z. H., Yao, Q. W., Ma, C. H., et al. Analysis of clinical characteristics and prognostic factors of severe COVID-19. Beijing Medical Journal. (2020) 42, 920-924. doi:10.15932/j.0253-9713.2020.10.003.

84. Shang, Y., Liu, T., Wei, Y., Li, J., Shao, L., Liu, M., et al. Scoring systems for predicting mortality for severe patients with COVID-19. EClinicalMedicine. (2020) 24, 100426. doi:10.1016/j.eclinm.2020.100426.

85. Shen, J. X., Zhuang, Z. H., Zhang, Q. X., Huang, J. F., Chen, G. P., Fang, Y. Y., et al. Risk Factors and Prognosis in Patients with COVID-19 and Liver Injury: A Retrospective Analysis. Journal of multidisciplinary healthcare. (2021) 14, 629-637. doi:10.2147/jmdh.s293378.

86. Shi, P., Ren, G., Yang, J., Li, Z., Deng, S., Li, M., et al. Clinical characteristics of imported and second-generation coronavirus disease 2019 (COVID-19) cases in Shaanxi outside Wuhan, China: a multicentre retrospective study. Epidemiology and infection. (2020) 148, e238. doi:10.1017/s0950268820002332.

87. Shi, Q., Zhang, X., Jiang, F., Zhang, X., Hu, N., Bimu, C., et al. Clinical Characteristics and Risk Factors for Mortality of COVID-19 Patients With Diabetes in Wuhan, China: A Two-Center, Retrospective Study. Diabetes care. (2020) 43, 1382-1391. doi:10.2337/dc20-0598.

88. Shu, Z., Zhou, Y., Chang, K., Liu, J., Min, X., Zhang, Q., et al. Clinical features and the traditional Chinese medicine therapeutic characteristics of 293 COVID-19 inpatient cases. Frontiers of medicine. (2020) 14, 760-775. doi:10.1007/s11684-020-0803-8.

89. Song, F., Ma, H., Wang, S., Qin, T., Xu, Q., Yuan, H., et al. Nutritional screening based on objective indices at admission predicts in-hospital mortality in patients with COVID-19. Nutrition journal. (2021) 20, 46. doi:10.1186/s12937-021-00702-8.

90. Sun, F., Kou, H., Wang, S., Lu, Y., Zhao, H., Li, W., et al. An analytical study of drug utilization, disease progression, and adverse events among 165 COVID-19 patients. Annals of translational medicine. (2021) 9, 306. doi:10.21037/atm-20-4960.

91. Sun, H., Ning, R., Tao, Y., Yu, C., Deng, X., Zhao, C., et al. Risk Factors for Mortality in 244 Older Adults With COVID-19 in Wuhan, China: A Retrospective Study. Journal of the American Geriatrics Society. (2020) 68, E19-e23. doi:10.1111/jgs.16533.

92. Sun, J. H., He, X. W., Chen, C. Y., Wang, Y., Zeng, H. S. Risk factors of deaths in patients with COVID-19: a multivariate logistic regression analysis. Journal of Critical Care in Internal Medicine. (2020) 26, 364-368.

93. Sun, Y., Sun, W., Ye, J., Yu, W. L., Chen, H., Shan, N. B., et al. Analysis of the clinical characteristics of novel coronavirus pneumonia and the influencing factors of severe disease progress. Chinese Journal of Emergency Medicine. (2020) 29, 901-907.

94. Tang, H. Y., Zhang, J., Peng, S. R., Hong, Y. J. Analysis of clinical characteristics of 225 elderly patients with COVID-19. The Journal of Practical Medicine. (2020) 36, 2616-2620.

95. Tao, F., Yang, F., Ruan, X. X., Xia, D. Z., Li, J. S., Wu, X. Clinical characteristics of 382 hospitalized patients with COVID-19. The Journal of Practical Medicine. (2020) 36, 1705-1709.

96. Tong, X., Cheng, A., Yuan, X., Zhong, X., Wang, H., Zhou, W., et al. Characteristics of peripheral white blood cells in COVID-19 patients revealed by a retrospective cohort study. BMC infectious diseases. (2021) 21, 1236. doi:10.1186/s12879-021-06899-7.

97. Wan, S., Xiang, Y., Fang, W., Zheng, Y., Li, B., Hu, Y., et al. Clinical features and treatment of COVID-19 patients in northeast Chongqing. Journal of medical virology. (2020) 92, 797-806. doi:10.1002/jmv.25783.

98. Wang, B., Sheng, J., Liu, X. Y., Jiang, Y. H., Yao, L., Xu, F., et al. Analysis of clinical characteristics of 136 patients with novel coronavirus pneumonia. Chinese Journal of Infectious Diseases. (2020) 38, 662-665.

99. Wang, D., Hu, B., Hu, C., Zhu, F., Liu, X., Zhang, J., et al. Clinical Characteristics of 138 Hospitalized Patients With 2019 Novel Coronavirus-Infected Pneumonia in Wuhan, China. Jama. (2020) 323, 1061-1069. doi:10.1001/jama.2020.1585.

100. Wang, D., Li, R., Wang, J., Jiang, Q., Gao, C., Yang, J., et al. Correlation analysis between disease severity and clinical and biochemical characteristics of 143 cases of COVID-19 in Wuhan, China: a descriptive study. BMC infectious diseases. (2020) 20, 519. doi:10.1186/s12879-020-05242-w.

101. Wang, D., Yin, Y., Hu, C., Liu, X., Zhang, X., Zhou, S., et al. Clinical course and outcome of 107 patients infected with the novel coronavirus, SARS-CoV-2, discharged from two hospitals in Wuhan, China. Critical care (London, England). (2020) 24, 188. doi:10.1186/s13054-020-02895-6.

102. Wang, G. Z., Yao, Y. P., Shi, L., Chen, H., Zhang, C., Zhen, C., et al. Association between major complications and underlying diseases in COVID-19 patients: An analysis of 2 079 cases. Academic Journal of Chinese PLA Medical School. (2021) 42, 477-482.

103. Wang, H., Li, Z., Xu, D. Y., Qian, X. D., Xu, H. F., Lu, B. Clinical characteristics of critically ill patients with novel coronavirus pneumonia. Journal of Southeast University(Medical Science Edition). (2020) 39, 475-479.

104. Wang, J. H., Chen, R. D., Yang, H. K., Zeng, L. C., Chen, H., Hou, Y. Y., et al. Inflammation-associated factors for predicting in-hospital mortality in patients with COVID-19. Journal of medical virology. (2021) 93, 2908-2917. doi:10.1002/jmv.26771.

105. Wang, K., Zuo, P., Liu, Y., Zhang, M., Zhao, X., Xie, S., et al. Clinical and Laboratory Predictors of In-hospital Mortality in Patients With Coronavirus Disease-2019: A Cohort Study in Wuhan, China. Clinical infectious diseases : an official publication of the Infectious Diseases Society of America. (2020) 71, 2079-2088. doi:10.1093/cid/ciaa538.

106. Wang, L., Cheng, X., Dong, Q., Zhou, C., Wang, Y., Song, B., et al. The characteristics of laboratory tests at admission and the risk factors for adverse clinical outcomes of severe and critical COVID-19 patients. BMC infectious diseases. (2021) 21, 371. doi:10.1186/s12879-021-06057-z.

107. Wang, L., Li, X., Chen, H., Yan, S., Li, D., Li, Y., et al. Coronavirus Disease 19 Infection Does Not Result in Acute Kidney Injury: An Analysis of 116 Hospitalized Patients from Wuhan, China. American journal of nephrology. (2020) 51, 343-348. doi:10.1159/000507471.

108. Wang, L., Lv, Q., Zhang, X., Jiang, B., Liu, E., Xiao, C., et al. The utility of MEWS for predicting the mortality in the elderly adults with COVID-19: a retrospective cohort study with comparison to other predictive clinical scores. PeerJ. (2020) 8, e10018. doi:10.7717/peerj.10018.

109. Wang, M., Fan, Y., Chai, Y., Cheng, W., Wang, K., Cao, J., et al. Association of Clinical and Immunological Characteristics With Disease Severity and Outcomes in 211 Patients With COVID-19 in Wuhan, China. Frontiers in cellular and infection microbiology. (2021) 11, 667487. doi:10.3389/fcimb.2021.667487.

110. Wang, Q., Cheng, J., Shang, J., Wang, Y., Wan, J., Yan, Y. Q., et al. Clinical value of laboratory indicators for predicting disease progression and death in patients with COVID-19: a retrospective cohort study. BMJ open. (2021) 11, e043790. doi:10.1136/bmjopen-2020-043790.

111. Wang, Q., Yao, Y., Huang, Z., Cao, J., Zhu, C., Yu, K., et al. Low CD4 T cell count predicts radiological progression in severe and critically ill COVID-19 patients: a case control study. Journal of thoracic disease. (2021) 13, 4723-4730. doi:10.21037/jtd-20-1848.

112. Wang, R., He, M., Yin, W., Liao, X., Wang, B., Jin, X., et al. The Prognostic Nutritional Index is associated with mortality of COVID-19 patients in Wuhan, China. Journal of clinical laboratory analysis. (2020) 34, e23566. doi:10.1002/jcla.23566.

113. Wang, T., Gao, S. Y., Bao, X. X., Bian, J. M. Clinical characteristics of 228 hospitalized patients with COVID-19. Medical Journal of Wuhan University. (2021) 42, 709-713. doi:10.14188/j.1671-8852.2020.0474.

114. Wang, W., Xin, C., Xiong, Z., Yan, X., Cai, Y., Zhou, K., et al. Clinical Characteristics and Outcomes of 421 Patients With Coronavirus Disease 2019 Treated in a Mobile Cabin Hospital. Chest. (2020) 158, 939-946. doi:10.1016/j.chest.2020.05.515.

115. Wang, X., Fang, J., Zhu, Y., Chen, L., Ding, F., Zhou, R., et al. Clinical characteristics of non-critically ill patients with novel coronavirus infection (COVID-19) in a Fangcang Hospital. Clinical microbiology and infection : the official publication of the European Society of Clinical Microbiology and Infectious Diseases. (2020) 26, 1063-1068. doi:10.1016/j.cmi.2020.03.032.

116. Wang, X., Yang, J. J., Tao, Y., Liu, C. Y., Zhang, N., Fu, X. N., et al. Risk factors and their influence on the COVID-19 patients with different outcome. Chinese Journal of Clinical Medicine. (2020) 27, 183-188.

117. Wang, Y., Liao, B., Guo, Y., Li, F., Lei, C., Zhang, F., et al. Clinical Characteristics of Patients Infected With the Novel 2019 Coronavirus (SARS-Cov-2) in Guangzhou, China. Open forum infectious diseases. (2020) 7, ofaa187. doi:10.1093/ofid/ofaa187.

118. Wang, Y., Yan, X., Huang, C., Sun, Y., Yao, C., Lin, Y., et al. Risk factors of mortality and contribution of treatment in patients infected with COVID-19: a retrospective propensity score matched study. Current medical research and opinion. (2021) 37, 13-19. doi:10.1080/03007995.2020.1853508.

119. Wang, Y., Zhang, Z. B., Zhang, D. P., Zhou, Z., Huang, S. Y., Xiao, X. Analysis of clinical characteristics of 125 cases of novel coronavirus pneumonia in Xiaogan City, Hubei Province. Chinese Journal of Infectious Diseases. (2020) 38, 806-809.

120. Wang, Y., Zhou, Y., Yang, Z., Xia, D., Hu, Y., Geng, S. Clinical Characteristics of Patients with Severe Pneumonia Caused by the SARS-CoV-2 in Wuhan, China. Respiration; international review of thoracic diseases. (2020) 99, 649-657. doi:10.1159/000507940.

121. Wang, Y. B., Luo, J., Wang, J. W., Zhang, F. C., Pan, Y. J., Chen, M. H., et al. Analysis of clinical characteristics of patients with different types of coronavirus disease 2019. Chinese Journal of Infectious Diseases. (2020) 38, 777-781.

122. Wang, Z., Wang, Z. Identification of risk factors for in-hospital death of COVID - 19 pneumonia -- lessions from the early outbreak. BMC infectious diseases. (2021) 21, 113. doi:10.1186/s12879-021-05814-4.

123. Wei, Y., Zeng, W., Huang, X., Li, J., Qiu, X., Li, H., et al. Clinical characteristics of 276 hospitalized patients with coronavirus disease 2019 in Zengdu District, Hubei Province: a single-center descriptive study. BMC infectious diseases. (2020) 20, 549. doi:10.1186/s12879-020-05252-8.

124. Wu, G., Yang, P., Xie, Y., Woodruff, H. C., Rao, X., Guiot, J., et al. Development of a clinical decision support system for severity risk prediction and triage of COVID-19 patients at hospital admission: an international multicentre study. The European respiratory journal. (2020) 56. doi:10.1183/13993003.01104-2020.

125. Wu, X., Li, C., Chen, S., Zhang, X., Wang, F., Shi, T., et al. Association of body mass index with severity and mortality of COVID-19 pneumonia: a two-center, retrospective cohort study from Wuhan, China. Aging. (2021) 13, 7767-7780. doi:10.18632/aging.202813.

126. Xiao, F., Ma, L. X., Lu, E. F., Huang, L. P., Yin, G. D., Pan, J. H., et al. Analysis of clinical characteristics and factors influencing death in patients with COVID-19. Journal of Guangxi Medical University. (2021) 38, 1755-1759. doi:10.16190/j.cnki.45-1211/r.2021.09.019.

127. Xie, D., Chen, J. C., Qiu, T., Zhao, C. Analysis of clinical characteristics of 118 patients with severe COVID-19. Journal of Jiangsu University(Medicine Edition). (2021) 31, 139-142. doi:10.13312/j.issn.1671-7783.y200217.

128. Xie, J., Shi, D., Bao, M., Hu, X., Wu, W., Sheng, J., et al. A Predictive Nomogram for Predicting Improved Clinical Outcome Probability in Patients with COVID-19 in Zhejiang Province, China. Engineering (Beijing, China). (2020). doi:10.1016/j.eng.2020.05.014.

129. Xie, X. Y., Guo, W., Lei, Y., Li, S. S., Wang, X. K., Wang, J., et al. COVID-19 Clinical Characteristics and Risk Factors of Severe COVID-19 Patients. Medical & Pharmaceutical Journal of Chinese People’s Liberation Army. (2020) 32, 1-5.

130. Xiong, P., Sun, W. W., Zhu, M., Zhu, M. C., Li, J., Yu, L. Clinical characteristics of 660 patients with COVID-19. The Journal of Practical Medicine. (2020) 36, 1710-1715.

131. Xiong, S., Liu, L., Lin, F., Shi, J., Han, L., Liu, H., et al. Clinical characteristics of 116 hospitalized patients with COVID-19 in Wuhan, China: a single-centered, retrospective, observational study. BMC infectious diseases. (2020) 20, 787. doi:10.1186/s12879-020-05452-2.

132. Xiong, W., Wang, H. R., Wu, X. Y., Fan, Y. Z., He, T., Luo, H. M., et al. Clinical characteristics on admission and risk factors for fatal outcome in patients with COVID-19. Medical Journal of Wuhan University. (2021) 42, 6-9. doi:10.14188/j.1671-8852.2020.0537.

133. Xiong, Y., Ma, Y., Tian, Y., Zhang, C., Yang, W., Liu, B., et al. A Longitudinal Cohort Study Using a Modified Child-Pugh Score to Escalate Respiratory Support in COVID-19 Patients - Hubei Province, China, 2020. China CDC weekly. (2021) 3, 423-429. doi:10.46234/ccdcw2021.113.

134. Xu, B., Fan, C. Y., Wang, A. L., Zou, Y. L., Yu, Y. H., He, C., et al. Suppressed T cell-mediated immunity in patients with COVID-19: A clinical retrospective study in Wuhan, China. The Journal of infection. (2020) 81, e51-e60. doi:10.1016/j.jinf.2020.04.012.

135. Xu, H., Chen, H., Huang, W. J. Clinical characteristics and risk factors affecting outcomes of the patients with severe coronavirus disease 2019. Medical Journal of Chinese People’s Liberation Army. (2021) 46, 267-273.

136. Xu, H., Wan, X. W. Analysis of clinical characteristics of 221 patients with novel coronavirus pneumonia. Journal of Hubei University of Science and Technology(Medical Sciences). (2021) 35, 214-216. doi:10.16751/j.cnki.2095-4646.2021.03.0214.

137. Xu, J., Gao, Y., Hu, S., Li, S., Wang, W., Wu, Y., et al. A composite risk model predicts disease progression in early stages of COVID-19: A propensity score-matched cohort study. Annals of clinical biochemistry. (2021) 58, 434-444. doi:10.1177/00045632211011194.

138. Xu, J., Yang, X., Yang, L., Zou, X., Wang, Y., Wu, Y., et al. Clinical course and predictors of 60-day mortality in 239 critically ill patients with COVID-19: a multicenter retrospective study from Wuhan, China. Critical care (London, England). (2020) 24, 394. doi:10.1186/s13054-020-03098-9.

139. Xu, W., Huang, C., Fei, L., Li, Q., Chen, L. Dynamic Changes in Liver Function Tests and Their Correlation with Illness Severity and Mortality in Patients with COVID-19: A Retrospective Cohort Study. Clinical interventions in aging. (2021) 16, 675-685. doi:10.2147/cia.s303629.

140. Yan, X., Han, X., Peng, D., Fan, Y., Fang, Z., Long, D., et al. Clinical Characteristics and Prognosis of 218 Patients With COVID-19: A Retrospective Study Based on Clinical Classification. Frontiers in medicine. (2020) 7, 485. doi:10.3389/fmed.2020.00485.

141. Yang, C. H., Xiong, R., Hu, X. L., Fan, X. P., Xu, F. Clinical Characteristics,Antibody and Nucleic Acid Test Results in COVID-19 Patients with Different Clinical Types. Practical Journal of Cardiac Cerebral Pneumal and Vascular Disease. (2020) 28, 10-15.

142. Yang, L., Liu, J., Zhang, R., Li, M., Li, Z., Zhou, X., et al. Epidemiological and clinical features of 200 hospitalized patients with corona virus disease 2019 outside Wuhan, China: A descriptive study. Journal of clinical virology : the official publication of the Pan American Society for Clinical Virology. (2020) 129, 104475. doi:10.1016/j.jcv.2020.104475.

143. Yang, M., Chen, X., Xu, Y. A Retrospective Study of the C-Reactive Protein to Lymphocyte Ratio and Disease Severity in 108 Patients with Early COVID-19 Pneumonia from January to March 2020 in Wuhan, China. Medical science monitor : international medical journal of experimental and clinical research. (2020) 26, e926393. doi:10.12659/msm.926393.

144. Yang, Q., Xie, L., Zhang, W., Zhao, L., Wu, H., Jiang, J., et al. Analysis of the clinical characteristics, drug treatments and prognoses of 136 patients with coronavirus disease 2019. Journal of clinical pharmacy and therapeutics. (2020) 45, 609-616. doi:10.1111/jcpt.13170.

145. Yang, Q., Zhou, Y., Wang, X., Gao, S., Xiao, Y., Zhang, W., et al. Effect of hypertension on outcomes of adult inpatients with COVID-19 in Wuhan, China: a propensity score-matching analysis. Respiratory research. (2020) 21, 172. doi:10.1186/s12931-020-01435-8.

146. Yang, Q. X., Xie, L., Wu, H. J., Jiang, J., Zhao, L., Dong, H., et al. [The Clinical Characteristics and Influencing Factors of Patients with Severe COVID-19]. Zhongguo shi yan xue ye xue za zhi. (2021) 29, 1295-1300. doi:10.19746/j.cnki.issn.1009-2137.2021.04.044.

147. Yi, C. F., Zhang, F., Yang, L., Fan, H. R., Chen, C. G., Zhu, W., et al. Correlation analysis of myocardial injury and inflammation in patients with COVID-19. The Journal of Practical Medicine. (2020) 36, 3174-3178.

148. Yi, P., Yang, X., Ding, C., Chen, Y., Xu, K., Ni, Q., et al. Risk factors and clinical features of deterioration in COVID-19 patients in Zhejiang, China: a single-centre, retrospective study. BMC infectious diseases. (2020) 20, 943. doi:10.1186/s12879-020-05682-4.

149. Yin, Z. W., Zhu, H., Tu, Q. Clinical Characteristics and Severe Risk Factors of COVID-19 Patients:A 474-Case Study. Journal of Jianghan University(Natural Science Edition). (2021) 49, 5-11. doi:10.16389/j.cnki.cn42-1737/n.2021.02.001.

150. Yu, C., Lei, Q., Li, W., Wang, X., Li, W., Liu, W. Epidemiological and clinical characteristics of 1663 hospitalized patients infected with COVID-19 in Wuhan, China: a single-center experience. Journal of infection and public health. (2020) 13, 1202-1209. doi:10.1016/j.jiph.2020.07.002.

151. Yu, C., Lei, Q., Li, W., Wang, X., Liu, W., Fan, X., et al. Clinical Characteristics, Associated Factors, and Predicting COVID-19 Mortality Risk: A Retrospective Study in Wuhan, China. American journal of preventive medicine. (2020) 59, 168-175. doi:10.1016/j.amepre.2020.05.002.

152. Yu, H. Z., Shao, H. X., Xing, Z. H., Qin, Z. H., Fu, S. S., Hu, S., et al. Analysis of clinical characteristics of patients with COVID-19 in Tianjin. Tianjin Medical Journal. (2020) 48, 577-582.

153. Yu, X. M., He, W. B., Zhou, J. L., Wang, Y. T., Wang, L. Changes of lymphocyte and subsets counts and the correlation to the prognosis in COVID-19 patients. Medical Journal of Wuhan University. (2021) 42, 355-358+363. doi:10.14188/j.1671-8852.2020.0198.

154. Yuan, L. Y., Xie, M. S., Li, L., Deng, T., Wang, M., Sun, Y., et al. The Ratio of Neutrophil/Lymphocyte and D-dimer/Platelet Ratio in Predicting the Risk of Death in Patients with New Coronavirus Pneumonia. Chinese Journal of Microcirculation. (2021) 31, 47-52.

155. Yuan, X. F., Fan, X. Y., Ren, N. Risk factors for severe and critical COVID-19. Practical Preventive Medicine. (2021) 28, 132-135.

156. YUna, J., Sun, Y. Y., Zuo, Y. J., Chen, T. Y., Cao, Q., Yuan, G. D., et al. A Retrospective Analysis of the Clinical Characteristics of 223 NCP Patients in Chongqing. Journal of Southwest University(Natural Science Edition). (2020) 42, 17-24. doi:10.13718/j.cnki.xdzk.2020.03.003.

157. Zeng, H. L., Lu, Q. B., Yang, Q., Wang, X., Yue, D. Y., Zhang, L. K., et al. Longitudinal Profile of Laboratory Parameters and Their Application in the Prediction for Fatal Outcome Among Patients Infected With SARS-CoV-2: A Retrospective Cohort Study. Clinical infectious diseases : an official publication of the Infectious Diseases Society of America. (2021) 72, 626-633. doi:10.1093/cid/ciaa574.

158. Zeng, H. L., Yang, Q., Yuan, P., Wang, X., Cheng, L. Associations of essential and toxic metals/metalloids in whole blood with both disease severity and mortality in patients with COVID-19. FASEB journal : official publication of the Federation of American Societies for Experimental Biology. (2021) 35, e21392. doi:10.1096/fj.202002346RR.

159. Zeng, J. H., Wu, W. B., Qu, J. X., Wang, Y., Dong, C. F., Luo, Y. F., et al. Cardiac manifestations of COVID-19 in Shenzhen, China. Infection. (2020) 48, 861-870. doi:10.1007/s15010-020-01473-w.

160. Zeng, Z., Ma, Y., Zeng, H., Huang, P., Liu, W., Jiang, M., et al. Simple nomogram based on initial laboratory data for predicting the probability of ICU transfer of COVID-19 patients: Multicenter retrospective study. Journal of medical virology. (2021) 93, 434-440. doi:10.1002/jmv.26244.

161. Zhang, G., Hu, C., Luo, L., Fang, F., Chen, Y., Li, J., et al. Clinical features and short-term outcomes of 221 patients with COVID-19 in Wuhan, China. Journal of clinical virology : the official publication of the Pan American Society for Clinical Virology. (2020) 127, 104364. doi:10.1016/j.jcv.2020.104364.

162. Zhang, J., Hao, Y., Ou, W., Ming, F., Liang, G., Qian, Y., et al. Serum interleukin-6 is an indicator for severity in 901 patients with SARS-CoV-2 infection: a cohort study. Journal of translational medicine. (2020) 18, 406. doi:10.1186/s12967-020-02571-x.

163. Zhang, J., Wang, X., Jia, X., Li, J., Hu, K., Chen, G., et al. Risk factors for disease severity, unimprovement, and mortality in COVID-19 patients in Wuhan, China. Clinical microbiology and infection : the official publication of the European Society of Clinical Microbiology and Infectious Diseases. (2020) 26, 767-772. doi:10.1016/j.cmi.2020.04.012.

164. Zhang, J., Wang, Z., Wang, X., Hu, Z., Yang, C., Lei, P. Risk Factors for Mortality of COVID-19 Patient Based on Clinical Course: A Single Center Retrospective Case-Control Study. Frontiers in immunology. (2021) 12, 581469. doi:10.3389/fimmu.2021.581469.

165. Zhang, J., Yu, M., Tong, S., Liu, L. Y., Tang, L. V. Predictive factors for disease progression in hospitalized patients with coronavirus disease 2019 in Wuhan, China. Journal of clinical virology : the official publication of the Pan American Society for Clinical Virology. (2020) 127, 104392. doi:10.1016/j.jcv.2020.104392.

166. Zhang, J. H., Xu, L. S., Guo, Y. M., Zhang, J. G., Tao, Z. M. Clinical characteristics and changes of myocardial injury markers in COVID-19 patients. New Medicine. (2021) 31, 342-349.

167. Zhang, J. J., Dong, X., Cao, Y. Y., Yuan, Y. D., Yang, Y. B., Yan, Y. Q., et al. Clinical characteristics of 140 patients infected with SARS-CoV-2 in Wuhan, China. Allergy. (2020) 75, 1730-1741. doi:10.1111/all.14238.

168. Zhang, L., Jiang, W., Zheng, Y. W., Yan, Z., Wu, B. B., Li, D. S., et al. Risk factors for disease severity of COVID-19. The Journal of Practical Medicine. (2020) 36, 1866-1871.

169. Zhang, L. H., Chen, H., Zhang, L., Chen, X. R. Analysis of the clinical characteristics of patients infected with novel coronavirus pneumonia in Chengdu. Modern Preventive Medicine. (2020) 47, 2276-2281.

170. Zhang, L. Y., Chen, P., Zheng, Q., Jiang, L. B., Tang, S. J. Analysis of clinical characteristics and test results of 478 COVID-19 patients. Chongqing Medicine. (2021) 50, 4232-4236.

171. Zhang, Q., Wang, Z., Lv, Y., Zhao, J., Dang, Q., Xu, D., et al. Clinical features and prognostic factors of patients with COVID-19 in Henan Province, China. Human cell. (2021) 34, 419-435. doi:10.1007/s13577-021-00499-y.

172. Zhang, Q. X., Yang, Q. X., Lian, N. F., Wang, C. Y., Jin, Y. X., Chen, G. P. Risk Factors of Acute Kidney Injury in Patients with Coronavirus Disease 2019. Journal of Fujian Medical University. (2021) 55, 237-241.

173. Zhang, R., Ouyang, H., Fu, L., Wang, S., Han, J., Huang, K., et al. CT features of SARS-CoV-2 pneumonia according to clinical presentation: a retrospective analysis of 120 consecutive patients from Wuhan city. European radiology. (2020) 30, 4417-4426. doi:10.1007/s00330-020-06854-1.

174. Zhang, S., Liu, L., Yang, B., Li, R., Luo, J., Huang, J., et al. Clinical characteristics of 134 convalescent patients with COVID-19 in Guizhou, China. Respiratory research. (2020) 21, 314. doi:10.1186/s12931-020-01580-0.

175. Zhang, W., Shen, J., Xu, Y. X., Su, Q., Deng, H. B. Value of cystatin C level to predicting in-hospital mortality of patients with COVID-19. Journal of Clinical Pulmonary Medicine. (2021) 26, 1472-1475.

176. Zhang, X. B., Hu, L., Ming, Q., Wei, X. J., Zhang, Z. Y., Chen, L. D., et al. Risk factors for mortality of coronavirus disease-2019 (COVID-19) patients in two centers of Hubei province, China: A retrospective analysis. PloS one. (2021) 16, e0246030. doi:10.1371/journal.pone.0246030.

177. Zhang, Y., Xiao, L. S., Li, P., Zhu, H., Hu, C., Zhang, W. F., et al. Clinical Characteristics of Patients With Progressive and Non-progressive Coronavirus Disease 2019: Evidence From 365 Hospitalised Patients in Honghu and Nanchang, China. Frontiers in medicine. (2020) 7, 556818. doi:10.3389/fmed.2020.556818.

178. Zhao, C., Bai, Y., Wang, C., Zhong, Y., Lu, N., Tian, L., et al. Risk factors related to the severity of COVID-19 in Wuhan. International journal of medical sciences. (2021) 18, 120-127. doi:10.7150/ijms.47193.

179. Zhao, C. C., Xu, H., Li, S. H., Gao, Y. Q., Zhu, Y., Wang, X. L., et al. Comparison of CT and clinical features between common and severe/critical type of COVID-19 patients. International Journal of Medical Radiology. (2020) 43, 257-261. doi:10.19300/j.2020.l18073.

180. Zheng, Y. Q., Liu, L., Cai, L. P., Wu, W. L., Tang, S., Zhang, Y. H., et al. Analysis of Clinical Characteristics of 224 Cases with COVID-19. Military Medical Journal of South China. (2020) 34, 412-415. doi:10.13730/j.issn.1009-2595.2020.06.010.

181. Zhong, J. N., Gao, X. Y., Chen, S., Li, C. H. Clinical characteristics of patients with COVID-19:A 764-case study. The Journal of Practical Medicine. (2020) 36, 2325-2328.

182. Zhou, C., Guo, H. H., Peng, B., Xi, J. L. Clinical features of 511 COVID-19 cases in Wuhan. International Journal of Virology. (2021) 28, 313-317.

183. Zhou, F., Yu, T., Du, R., Fan, G., Liu, Y., Liu, Z., et al. Clinical course and risk factors for mortality of adult inpatients with COVID-19 in Wuhan, China: a retrospective cohort study. Lancet (London, England). (2020) 395, 1054-1062. doi:10.1016/s0140-6736(20)30566-3.

184. Zhou, J., Huang, L., Chen, J., Yuan, X., Shen, Q., Dong, S., et al. Clinical features predicting mortality risk in older patients with COVID-19. Current medical research and opinion. (2020) 36, 1753-1759. doi:10.1080/03007995.2020.1825365.

185. Zhou, Q. L., Lu, H., Xiong, F., Liu, C., Pan, P. F. Analysis on clinical characteristics and prognosis in 248 cases of COVID-19 in Chongqing city. Journal of Modern Medicine & Health. (2021) 37, 2559-2564.

186. Zhou, S., Mi, S., Luo, S., Wang, Y., Ren, B., Cai, L., et al. Risk Factors for Mortality in 220 Patients With COVID-19 in Wuhan, China: A Single-Center, Retrospective Study. Ear, nose, & throat journal. (2021) 100, 140s-147s. doi:10.1177/0145561320972608.

187. Zou, L., Dai, L., Zhang, Y., Fu, W., Gao, Y., Zhang, Z., et al. Clinical Characteristics and Risk Factors for Disease Severity and Death in Patients With Coronavirus Disease 2019 in Wuhan, China. Frontiers in medicine. (2020) 7, 532. doi:10.3389/fmed.2020.00532.

# Supplementary Table 2 Quality assessment by Joanna Briggs Institute Prevalence Critical Appraisal Checklist

| **Author Year** | **Was the sample representative of the target population?** | **Were study participants recruited in an appropriate way?** | **Was the sample size adequate?** | **Were the study subjects and the setting described in detail?** | **Was the data analysis conducted with sufficient coverage of the identified sample?** | **Were objective, standard criteria used for the measurement of the condition?** | **Was the condition measured reliably?** | **Was there appropriate statistical analysis?** | **Are all important confounding factors/subgroups/differences identified and accounted for?** | **Quality Assessment** |
| --- | --- | --- | --- | --- | --- | --- | --- | --- | --- | --- |
| An YW 2021 | Yes | Yes | Yes | Yes | Yes | Yes | Yes | Yes | Yes | Low risk of bias |
| Cai QX 2020a | Yes | Yes | Yes | Yes | Yes | Yes | Yes | Yes | Yes | Low risk of bias |
| Cai QX 2020b | Yes | Yes | Yes | Yes | Yes | Yes | Yes | Yes | Yes | Low risk of bias |
| Caillon A 2021 | Yes | Yes | Yes | Yes | Unclear | Yes | Yes | Yes | Yes | Low risk of bias |
| Cao JL 2020 | Yes | Yes | Yes | Yes | Yes | Yes | Yes | Yes | Yes | Low risk of bias |
| Cao M 2020 | Yes | Yes | Yes | Yes | Yes | Yes | Yes | Yes | Yes | Low risk of bias |
| Cao YK 2020 | Yes | Yes | Yes | Yes | Yes | Yes | Yes | Yes | Yes | Low risk of bias |
| Chen FF 2020 | Yes | Yes | Yes | Yes | Yes | Yes | Yes | Yes | Yes | Low risk of bias |
| Chen HL 2021 | Yes | Yes | Yes | Yes | Yes | Yes | Yes | Yes | Yes | Low risk of bias |
| Chen L 2020a | Yes | Yes | Yes | Yes | Yes | Yes | Yes | Yes | Yes | Low risk of bias |
| Chen L 2020b | Yes | Yes | Yes | Yes | Yes | Yes | Yes | Yes | Yes | Low risk of bias |
| Chen QQ 2020 | Yes | Yes | Yes | Yes | Yes | Yes | Yes | Yes | Yes | Low risk of bias |
| Chen T 2020 | Yes | Yes | Yes | Yes | Yes | Yes | Yes | Yes | Yes | Low risk of bias |
| Chen Y 2021 | Yes | Yes | Yes | Yes | Yes | Yes | Yes | Yes | Yes | Low risk of bias |
| Chen YY 2021 | Yes | Yes | Yes | Yes | Unclear | Yes | Yes | Yes | Yes | Low risk of bias |
| Cheng B 2020 | Yes | Yes | Yes | Yes | Yes | Yes | Yes | Yes | Yes | Low risk of bias |
| Cheng KB 2021 | Yes | Yes | Yes | Yes | Unclear | Yes | Yes | Yes | Yes | Low risk of bias |
| Deng Q 2020 | Yes | Yes | Yes | Yes | Yes | Yes | Yes | Yes | Yes | Low risk of bias |
| Deng Y 2020 | Yes | Yes | Yes | Yes | Yes | Yes | Yes | Yes | Yes | Low risk of bias |
| Ding RR 2021 | Yes | Yes | Yes | Yes | Yes | Yes | Yes | Yes | Yes | Low risk of bias |
| Fan HZ 2021 | Yes | Yes | Yes | Yes | Yes | Yes | Yes | Yes | Yes | Low risk of bias |
| Fang X 2020 | Yes | Yes | Yes | Yes | Yes | Yes | Yes | Yes | Yes | Low risk of bias |
| Feng XB 2020 | Yes | Yes | Yes | Yes | Yes | Yes | Yes | Yes | Yes | Low risk of bias |
| Feng Y 2020 | Yes | Yes | Yes | Yes | Yes | Yes | Yes | Yes | Yes | Low risk of bias |
| Gao S 2020 | Yes | Yes | Yes | Yes | Yes | Yes | Yes | Yes | Yes | Low risk of bias |
| Guo XY 2020 | Yes | Yes | Yes | Yes | Yes | Yes | Yes | Yes | Yes | Low risk of bias |
| Hu L 2020 | Yes | Yes | Yes | Yes | Yes | Yes | Yes | Yes | Yes | Low risk of bias |
| Hu Q 2021a | Yes | Yes | Yes | Yes | Yes | Yes | Yes | Yes | Yes | Low risk of bias |
| Hu Q 2021b | Yes | Yes | Yes | Yes | Yes | Yes | Yes | Yes | Yes | Low risk of bias |
| Hu XS 2020 | Yes | Yes | Yes | Yes | Yes | Yes | Yes | Yes | Yes | Low risk of bias |
| Huang R 2020 | Yes | Yes | Yes | Yes | Yes | Yes | Yes | Yes | Yes | Low risk of bias |
| Huang YX 2020 | Yes | Yes | Yes | Yes | Yes | Yes | Yes | Yes | Yes | Low risk of bias |
| Jiang Y 2020 | Yes | Yes | Yes | Yes | Yes | Yes | Yes | Yes | Yes | Low risk of bias |
| Jiao L 2020 | Yes | Yes | Yes | Yes | Yes | Yes | Yes | Yes | Yes | Low risk of bias |
| Li HB 2020 | Yes | Yes | Yes | Yes | Yes | Yes | Yes | Yes | Yes | Low risk of bias |
| Li HY 2020 | Yes | Yes | Yes | Yes | Yes | Yes | Yes | Yes | Yes | Low risk of bias |
| Li J 2021 | Yes | Yes | Yes | Yes | Yes | Yes | Yes | Yes | Yes | Low risk of bias |
| Li JW 2021 | Yes | Yes | Yes | Yes | Yes | Yes | Yes | Yes | Yes | Low risk of bias |
| Li LZ 2021 | Yes | Yes | Yes | Yes | Yes | Yes | Yes | Yes | Yes | Low risk of bias |
| Li M 2020 | Yes | Yes | Yes | Yes | Yes | Yes | Yes | Yes | Yes | Low risk of bias |
| Li N 2020 | Yes | Yes | Yes | Yes | Yes | Yes | Yes | Yes | Yes | Low risk of bias |
| Li PP 2021 | Yes | Yes | Yes | Yes | Yes | Yes | Yes | Yes | Yes | Low risk of bias |
| Li QB 2020 | Yes | Yes | Yes | Yes | Unclear | Yes | Yes | Yes | Yes | Low risk of bias |
| Li RQ 2020 | Yes | Yes | Yes | Yes | Yes | Yes | Yes | Yes | Yes | Low risk of bias |
| Li T 2020 | Yes | Yes | Yes | Yes | Yes | Yes | Yes | Yes | Yes | Low risk of bias |
| Li WX 2021 | Yes | Yes | Yes | Yes | Yes | Yes | Yes | Yes | Yes | Low risk of bias |
| Li XC 2020 | Yes | Yes | Yes | Yes | Yes | Yes | Yes | Yes | Yes | Low risk of bias |
| Li XQ 2021 | Yes | Yes | Yes | Yes | Yes | Yes | Yes | Yes | Yes | Low risk of bias |
| Li Y 2020 | Yes | Yes | Yes | Yes | Yes | Yes | Yes | Yes | Yes | Low risk of bias |
| Li Y 2021a | Yes | Yes | Yes | Yes | Unclear | Yes | Yes | Yes | Yes | Low risk of bias |
| Li Y 2021b | Yes | Yes | Yes | Yes | Unclear | Yes | Yes | Yes | Yes | Low risk of bias |
| Liao DY 2020 | Yes | Yes | Yes | Yes | Unclear | Yes | Yes | Yes | Yes | Low risk of bias |
| Lin HY 2021 | Yes | Yes | Yes | Yes | Yes | Yes | Yes | Yes | Yes | Low risk of bias |
| Lin L 2020 | Yes | Yes | Yes | Yes | Yes | Yes | Yes | Yes | Yes | Low risk of bias |
| Liu D 2020 | Yes | Yes | Yes | Yes | Unclear | Yes | Yes | Yes | Yes | Low risk of bias |
| Liu FY 2020 | Yes | Yes | Yes | Yes | Yes | Yes | Yes | Yes | Yes | Low risk of bias |
| Liu J 2020 | Yes | Yes | Yes | Yes | Yes | Yes | Yes | Yes | Yes | Low risk of bias |
| Liu J 2021 | Yes | Yes | Yes | Yes | Yes | Yes | Yes | Yes | Yes | Low risk of bias |
| Liu JC 2021 | Yes | Yes | Yes | Yes | Yes | Yes | Yes | Yes | Yes | Low risk of bias |
| Liu MY 2021 | Yes | Yes | Yes | Yes | Unclear | Yes | Yes | Yes | Yes | Low risk of bias |
| Liu QQ 2020 | Yes | Yes | Yes | Yes | Yes | Yes | Yes | Yes | Yes | Low risk of bias |
| Liu SJ 2020 | Yes | Yes | Yes | Yes | Yes | Yes | Yes | Yes | Yes | Low risk of bias |
| Liu XQ 2022 | Yes | Yes | Yes | Yes | Yes | Yes | Yes | Yes | Yes | Low risk of bias |
| Liu Z 2021 | Yes | Yes | Yes | Yes | Yes | Yes | Yes | Yes | Yes | Low risk of bias |
| Liu ZZ 2020 | Yes | Yes | Yes | Yes | Yes | Yes | Yes | Yes | Yes | Low risk of bias |
| Long X 2020 | Yes | Yes | Yes | Yes | Yes | Yes | Yes | Yes | Yes | Low risk of bias |
| Lu ZL 2020 | Yes | Yes | Yes | Yes | Yes | Yes | Yes | Yes | Yes | Low risk of bias |
| Luo M 2020 | Yes | Yes | Yes | Yes | Yes | Yes | Yes | Yes | Yes | Low risk of bias |
| Lv ZH 2020 | Yes | Yes | Yes | Yes | Yes | Yes | Yes | Yes | Yes | Low risk of bias |
| Mi J 2020 | Yes | Yes | Yes | Yes | Yes | Yes | Yes | Yes | Yes | Low risk of bias |
| Mo J 2020 | Yes | Yes | Yes | Yes | Yes | Yes | Yes | Yes | Yes | Low risk of bias |
| Pan D 2020 | Yes | Yes | Yes | Yes | Yes | Yes | Yes | Yes | Yes | Low risk of bias |
| Pei GC 2020 | Yes | Yes | Yes | Yes | Yes | Yes | Yes | Yes | Yes | Low risk of bias |
| Peng F 2021 | Yes | Yes | Yes | Yes | Yes | Yes | Yes | Yes | Yes | Low risk of bias |
| Peng L 2021 | Yes | Yes | Yes | Yes | Yes | Yes | Yes | Yes | Yes | Low risk of bias |
| Peng S 2020 | Yes | Yes | Yes | Yes | Yes | Yes | Yes | Yes | Yes | Low risk of bias |
| Qi X 2021 | Yes | Yes | Yes | Yes | Yes | Yes | Yes | Yes | Yes | Low risk of bias |
| Qin SJ 2021 | Yes | Yes | Yes | Yes | Yes | Yes | Yes | Yes | Yes | Low risk of bias |
| Qin ZJ 2020 | Yes | Yes | Yes | Yes | Yes | Yes | Yes | Yes | Yes | Low risk of bias |
| Qu JJ 2020 | Yes | Yes | Yes | Yes | Yes | Yes | Yes | Yes | Yes | Low risk of bias |
| Rao XR 2020 | Yes | Yes | Yes | Yes | Unclear | Yes | Yes | Yes | Yes | Low risk of bias |
| Ren LY 2020 | Yes | Yes | Yes | Yes | Yes | Yes | Yes | Yes | Yes | Low risk of bias |
| Ren MX 2020 | Yes | Yes | Yes | Yes | Yes | Yes | Unclear | Yes | Yes | Low risk of bias |
| Shang YF 2020 | Yes | Yes | Yes | Yes | Yes | Yes | Yes | Yes | Yes | Low risk of bias |
| Shen JX 2021 | Yes | Yes | Yes | Yes | Yes | Yes | Yes | Yes | Yes | Low risk of bias |
| Shi PY 2020 | Yes | Yes | Yes | Yes | Yes | Yes | Yes | Yes | Yes | Low risk of bias |
| Shi Q 2020 | Yes | Yes | Yes | Yes | Yes | Yes | Yes | Yes | Yes | Low risk of bias |
| Shu ZX 2020 | Yes | Yes | Yes | Yes | Unclear | Yes | Yes | Yes | Yes | Low risk of bias |
| Song FE 2021 | Yes | Yes | Yes | Yes | Yes | Yes | Yes | Yes | Yes | Low risk of bias |
| Sun F 2021 | Yes | Yes | Yes | Yes | Yes | Yes | Yes | Yes | Yes | Low risk of bias |
| Sun HY 2020 | Yes | Yes | Yes | Yes | Yes | Yes | Yes | Yes | Yes | Low risk of bias |
| Sun JH 2020 | Yes | Yes | Yes | Yes | Yes | Yes | Yes | Yes | Yes | Low risk of bias |
| Sun Y 2020 | Yes | Yes | Yes | Yes | Yes | Yes | Yes | Yes | Yes | Low risk of bias |
| Tang HY 2020 | Yes | Yes | Yes | Yes | Yes | Yes | Yes | Yes | Yes | Low risk of bias |
| Tao F 2020 | Yes | Yes | Yes | Yes | Yes | Yes | Yes | Yes | Yes | Low risk of bias |
| Tong XL 2021 | Yes | Yes | Yes | Yes | Yes | Yes | Yes | Yes | Yes | Low risk of bias |
| Wan SX 2020 | Yes | Yes | Yes | Yes | Yes | Yes | Yes | Yes | Yes | Low risk of bias |
| Wang B 2020 | Yes | Yes | Yes | Yes | Yes | Yes | Yes | Yes | Yes | Low risk of bias |
| Wang D 2020 | Yes | Yes | Yes | Yes | Yes | Yes | Yes | Yes | Yes | Low risk of bias |
| Wang DW 2020a | Yes | Yes | Yes | Yes | Yes | Yes | Yes | Yes | Yes | Low risk of bias |
| Wang DW 2020b | Yes | Yes | Yes | Yes | Yes | Yes | Yes | Yes | Yes | Low risk of bias |
| Wang GZ 2021 | Yes | Yes | Yes | Yes | Unclear | Yes | Yes | Yes | Yes | Low risk of bias |
| Wang H 2020 | Yes | Yes | Yes | Yes | Yes | Yes | Yes | Yes | Yes | Low risk of bias |
| Wang JH 2021 | Yes | Yes | Yes | Yes | Yes | Yes | Yes | Yes | Yes | Low risk of bias |
| Wang K 2020 | Yes | Yes | Yes | Yes | Yes | Yes | Yes | Yes | Yes | Low risk of bias |
| Wang LC 2020 | Yes | Yes | Yes | Yes | Yes | Yes | Yes | Yes | Yes | Low risk of bias |
| Wang LL 2021 | Yes | Yes | Yes | Yes | Yes | Yes | Yes | Yes | Yes | Low risk of bias |
| Wang LW 2020 | Yes | Yes | Yes | Yes | Yes | Yes | Yes | Yes | Yes | Low risk of bias |
| Wang M 2021 | Yes | Yes | Yes | Yes | Yes | Yes | Yes | Yes | Yes | Low risk of bias |
| Wang Q 2021 | Yes | Yes | Yes | Yes | Yes | Yes | Yes | Yes | Yes | Low risk of bias |
| Wang QQ 2021 | Yes | Yes | Yes | Yes | Unclear | Yes | Yes | Yes | Yes | Low risk of bias |
| Wang RR 2020 | Yes | Yes | Yes | Yes | Yes | Yes | Yes | Yes | Yes | Low risk of bias |
| Wang T 2021 | Yes | Yes | Yes | Yes | Yes | Yes | Yes | Yes | Yes | Low risk of bias |
| Wang W 2020 | Yes | Yes | Yes | Yes | Yes | Yes | Yes | Yes | Yes | Low risk of bias |
| Wang X 2020a | Yes | Yes | Yes | Yes | Yes | Yes | Yes | Yes | Yes | Low risk of bias |
| Wang X 2020b | Yes | Yes | Yes | Yes | Yes | Yes | Unclear | Yes | Yes | Low risk of bias |
| Wang Y 2020 | Yes | Yes | Yes | Yes | Yes | Yes | Yes | Yes | Yes | Low risk of bias |
| Wang Y 2021 | Yes | Yes | Yes | Yes | Yes | Yes | Yes | Yes | Yes | Low risk of bias |
| Wang YB 2020 | Yes | Yes | Yes | Yes | Yes | Yes | Yes | Yes | Yes | Low risk of bias |
| Wang YF 2020 | Yes | Yes | Yes | Yes | Yes | Yes | Yes | Yes | Yes | Low risk of bias |
| Wang YP 2020 | Yes | Yes | Yes | Yes | Yes | Yes | Yes | Yes | Yes | Low risk of bias |
| Wang ZG 2021 | Yes | Yes | Yes | Yes | Yes | Yes | Yes | Yes | Yes | Low risk of bias |
| Wei YP 2020 | Yes | Yes | Yes | Yes | Yes | Yes | Yes | Yes | Yes | Low risk of bias |
| Wu GY 2020 | Yes | Yes | Yes | Yes | Yes | Yes | Yes | Yes | Yes | Low risk of bias |
| Wu XD 2020 | Yes | Yes | Yes | Yes | Yes | Yes | Yes | Yes | Yes | Low risk of bias |
| Xiao F 2021 | Yes | Yes | Yes | Yes | Yes | Yes | Yes | Yes | Yes | Low risk of bias |
| Xie D 2021 | Yes | Yes | Yes | Yes | Yes | Yes | Yes | Yes | Yes | Low risk of bias |
| Xie JJ 2020 | Yes | Yes | Yes | Yes | Yes | Yes | Yes | Yes | Yes | Low risk of bias |
| Xie XY 2020 | Yes | Yes | Yes | Yes | Yes | Yes | Yes | Yes | Yes | Low risk of bias |
| Xiong P 2020 | Yes | Yes | Yes | Yes | Yes | Yes | Yes | Yes | Yes | Low risk of bias |
| Xiong SQ 2020 | Yes | Yes | Yes | Yes | Yes | Yes | Yes | Yes | Yes | Low risk of bias |
| Xiong W 2021 | Yes | Yes | Yes | Yes | Yes | Yes | Yes | Yes | Yes | Low risk of bias |
| Xiong YB 2021 | Yes | Yes | Yes | Yes | Yes | Yes | Yes | Yes | Yes | Low risk of bias |
| Xu B 2020 | Yes | Yes | Yes | Yes | Yes | Yes | Yes | Yes | Yes | Low risk of bias |
| Xu H 2021a | Yes | Yes | Yes | Yes | Yes | Yes | Unclear | Yes | Yes | Low risk of bias |
| Xu H 2021b | Yes | Yes | Yes | Yes | Yes | Yes | Yes | Yes | Yes | Low risk of bias |
| Xu JJ 2021 | Yes | Yes | Yes | Yes | Yes | Yes | Yes | Yes | Yes | Low risk of bias |
| Xu JQ 2020 | Yes | Yes | Yes | Yes | Yes | Yes | Yes | Yes | Yes | Low risk of bias |
| Xu W 2021 | Yes | Yes | Yes | Yes | Yes | Yes | Yes | Yes | Yes | Low risk of bias |
| Yan XQ 2020 | Yes | Yes | Yes | Yes | Yes | Yes | Yes | Yes | Yes | Low risk of bias |
| Yang LH 2020 | Yes | Yes | Yes | Yes | Yes | Yes | Yes | Yes | Yes | Low risk of bias |
| Yang M 2020 | Yes | Yes | Yes | Yes | Yes | Yes | Yes | Yes | Yes | Low risk of bias |
| Yang Q 2020 | Yes | Yes | Yes | Yes | Yes | Yes | Yes | Yes | Yes | Low risk of bias |
| Yang QX 2020 | Yes | Yes | Yes | Yes | Yes | Yes | Yes | Yes | Yes | Low risk of bias |
| Yang QX 2021 | Yes | Yes | Yes | Yes | Yes | Yes | Yes | Yes | Yes | Low risk of bias |
| Yang XH 2020 | Yes | Yes | Yes | Yes | Yes | Yes | Yes | Yes | Yes | Low risk of bias |
| Yi CF 2020 | Yes | Yes | Yes | Yes | Yes | Yes | Yes | Yes | Yes | Low risk of bias |
| Yi P 2020 | Yes | Yes | Yes | Yes | Yes | Yes | Yes | Yes | Yes | Low risk of bias |
| Yin ZW 2021 | Yes | Yes | Yes | Yes | Unclear | Yes | Unclear | Yes | Yes | Moderate risk of bias |
| Yu CZ 2020a | Yes | Yes | Yes | Yes | Unclear | Yes | Yes | Yes | Yes | Low risk of bias |
| Yu CZ 2020b | Yes | Yes | Yes | Yes | Yes | Yes | Yes | Yes | Yes | Low risk of bias |
| Yu HZ 2020 | Yes | Yes | Yes | Yes | Yes | Yes | Yes | Yes | Yes | Low risk of bias |
| Yu XM 2021 | Yes | Yes | Yes | Yes | Yes | Yes | Yes | Yes | Yes | Low risk of bias |
| Yuan J 2020 | Yes | Yes | Yes | Yes | Yes | Yes | Yes | Yes | Yes | Low risk of bias |
| Yuan LY 2021 | Yes | Yes | Yes | Yes | Yes | Yes | Yes | Yes | Yes | Low risk of bias |
| Yuan XF 2021 | Yes | Yes | Yes | Yes | Yes | Yes | Yes | Yes | Yes | Low risk of bias |
| Zeng HL 2021a | Yes | Yes | Yes | Yes | Yes | Yes | Yes | Yes | Yes | Low risk of bias |
| Zeng HL 2021b | Yes | Yes | Yes | Yes | Yes | Yes | Yes | Yes | Yes | Low risk of bias |
| Zeng JH 2020 | Yes | Yes | Yes | Yes | Yes | Yes | Yes | Yes | Yes | Low risk of bias |
| Zeng ZH 2021 | Yes | Yes | Yes | Yes | Yes | Yes | Yes | Yes | Yes | Low risk of bias |
| Zhang GQ 2020 | Yes | Yes | Yes | Yes | Yes | Yes | Yes | Yes | Yes | Low risk of bias |
| Zhang J 2020a | Yes | Yes | Yes | Yes | Yes | Yes | Yes | Yes | Yes | Low risk of bias |
| Zhang J 2020b | Yes | Yes | Yes | Yes | Yes | Yes | Yes | Yes | Yes | Low risk of bias |
| Zhang J 2020c | Yes | Yes | Yes | Yes | Yes | Yes | Yes | Yes | Yes | Low risk of bias |
| Zhang J 2021 | Yes | Yes | Yes | Yes | Unclear | Yes | Yes | Yes | Yes | Low risk of bias |
| Zhang JH 2021 | Yes | Yes | Yes | Yes | Yes | Yes | Yes | Yes | Yes | Low risk of bias |
| Zhang JJ 2020 | Yes | Yes | Yes | Yes | Yes | Yes | Yes | Yes | Yes | Low risk of bias |
| Zhang L 2020 | Yes | Yes | Yes | Yes | Yes | Yes | Yes | Yes | Yes | Low risk of bias |
| Zhang LH 2020 | Yes | Yes | Yes | Yes | Yes | Yes | Yes | Yes | Yes | Low risk of bias |
| Zhang LY 2021 | Yes | Yes | Yes | Yes | Yes | Yes | Yes | Yes | Yes | Low risk of bias |
| Zhang QQ 2021 | Yes | Yes | Yes | Yes | Yes | Yes | Yes | Yes | Yes | Low risk of bias |
| Zhang QX 2021 | Yes | Yes | Yes | Yes | Yes | Yes | Yes | Yes | Yes | Low risk of bias |
| Zhang R 2020 | Yes | Yes | Yes | Yes | Yes | Yes | Yes | Yes | Yes | Low risk of bias |
| Zhang SQ 2020 | Yes | Yes | Yes | Yes | Yes | Yes | Yes | Yes | Yes | Low risk of bias |
| Zhang W 2021 | Yes | Yes | Yes | Yes | Yes | Yes | Yes | Yes | Yes | Low risk of bias |
| Zhang XB 2021 | Yes | Yes | Yes | Yes | Yes | Yes | Yes | Yes | Yes | Low risk of bias |
| Zhang YP 2020 | Yes | Yes | Yes | Yes | Unclear | Yes | Yes | Yes | Yes | Low risk of bias |
| Zhao C 2021 | Yes | Yes | Yes | Yes | Unclear | Yes | Yes | Yes | Yes | Low risk of bias |
| Zhao CC 2020 | Yes | Yes | Yes | Yes | Yes | Yes | Yes | Yes | Yes | Low risk of bias |
| Zheng YQ 2020 | Yes | Yes | Yes | Yes | Yes | Yes | Yes | Yes | Yes | Low risk of bias |
| Zhong JN 2020 | Yes | Yes | Yes | Yes | Yes | Yes | Yes | Yes | Yes | Low risk of bias |
| Zhou C 2021 | Yes | Yes | Yes | Yes | Yes | Yes | Unclear | Yes | Yes | Low risk of bias |
| Zhou F 2020 | Yes | Yes | Yes | Yes | Yes | Yes | Yes | Yes | Yes | Low risk of bias |
| Zhou J 2020 | Yes | Yes | Yes | Yes | Yes | Yes | Yes | Yes | Yes | Low risk of bias |
| Zhou QL 2021 | Yes | Yes | Yes | Yes | Yes | Yes | Yes | Yes | Yes | Low risk of bias |
| Zhou SL 2021 | Yes | Yes | Yes | Yes | Yes | Yes | Yes | Yes | Yes | Low risk of bias |
| Zou L 2020 | Yes | Yes | Yes | Yes | Yes | Yes | Yes | Yes | Yes | Low risk of bias |

Joanna Briggs Institute Prevalence Critical Appraisal Checklist, which each item is scored on a scale of 0 for“No” or “Unclear”, and 1 for “Yes”. Scores of studies are categorized as high risk of bias (0-5), moderate risk of bias (6-7), or low risk of bias (8-9).

# Supplementary Table 3 Pooled estimate prevalence of comorbidities and complications for COVID-19 in total

|  | **Pooled prevalence (95% CI)** | **I²** | **p value (Heterogeneity)** | **Number of studies** | **Number of participants** |
| --- | --- | --- | --- | --- | --- |
| **Comorbidities** |  |  |  |  |  |
| Hypertension | 27.16% (25.33-29.08) | 94.6% | 0 | 173 | 68465 |
| Diabetes | 12.78% (11.93-13.69) | 88.8% | < 0.0001 | 178 | 70939 |
| Malignancy | 2.89% (2.58-3.25) | 73.6% | < 0.0001 | 121 | 52994 |
| Cardiovascular diseases | 9.58% (7.82-11.68) | 95.4% | < 0.0001 | 68 | 26169 |
| Aorta sclerosis | 1.43% (0.36-5.53) | NR | NR | 1 | 140 |
| Arrhythmia | 4.38% (1.89-9.82) | 88.8% | < 0.0001 | 5 | 1197 |
| Atrial fibrillation | 4.83% (2.83-8.14) | 0.0% | 0.4194 | 2 | 269 |
| Cardiac insufficiency | 1.23% (0.83-1.82) | 0.0% | 0.7612 | 3 | 2030 |
| Chronic heart disease | 6.37% (4.53-8.88) | 90.8% | < 0.0001 | 16 | 6449 |
| Coronary artery disease | 8.59% (5.13-14.04) | 89.3% | < 0.0001 | 5 | 2308 |
| Coronary atherosclerosis | 5.42% (4.67-6.28) | NR | NR | 1 | 3044 |
| Coronary heart disease | 7.68% (6.62-8.89) | 87.4% | < 0.0001 | 66 | 28382 |
| Heart failure | 0.88% (0.37-2.10) | 0.0% | 0.377 | 3 | 566 |
| Myocardial infarction | 4.53% (1.07-17.16) | 87.3% | 0.005 | 2 | 795 |
| Cerebrovascular disease | 4.21% (3.63-4.87) | 79.4% | < 0.0001 | 72 | 27549 |
| Cerebral infarction | 3.69% (2.94-4.62) | 39.70% | 0.1566 | 5 | 3328 |
| Intracerebral hemorrhage | 0.58% (0.32-1.04) | 0.0% | 0.7969 | 2 | 1902 |
| Stroke | 2.77% (1.87-4.09) | 57.6% | 0.0208 | 8 | 4393 |
| Asthma | 0.47% (0.27-0.81) | 54.6% | 0.0857 | 4 | 5724 |
| Chronic bronchitis | 2.59% (1.21-5.47) | 90.7% | < 0.0001 | 5 | 3400 |
| Chronic lung disease | 5.02% (3.95-6.37) | 83.5% | < 0.0001 | 30 | 12954 |
| COPD | 3.05% (2.59-3.60) | 87.1% | < 0.0001 | 94 | 41507 |
| Respiratory disease | 5.58% (4.46-6.96) | 83.3% | < 0.0001 | 22 | 8296 |
| Tuberculosis | 1.22% (0.97-1.54) | 0.00% | 0.6515 | 9 | 5731 |
| Pulmonary emphysema | 2.54% (0.82-7.59) | NR | NR | 1 | 118 |
| Chronic kidney disease | 2.63% (2.22-3.11) | 85.2% | < 0.0001 | 89 | 37744 |
| kidney failure | 2.87% (1.67-4.87) | 42.6% | 0.1558 | 4 | 632 |
| renal insufficiency | 1.47% (0.83-2.61) | 82.5% | < 0.0001 | 9 | 5774 |
| Nephritis | 0.23% (0.11-0.48) | NR | NR | 1 | 3044 |
| Uremia | 4.70% (3.17-6.91) | NR | NR | 1 | 511 |
| Gallbladder disease | 2.31% (1.46-3.64) | 28.7% | 0.246 | 3 | 779 |
| Chronic liver disease | 3.61% (3.04-4.27) | 87.5% | < 0.0001 | 75 | 28303 |
| Cirrhosis | 0.62% (0.45-0.86) | 0.0% | 0.5815 | 4 | 5645 |
| Fatty liver | 8.78% (5.54-13.65) | 75.1% | 0.0029 | 5 | 1096 |
| Hepatitis B | 2.09% (1.30-3.35) | 79.5% | < 0.0001 | 8 | 3716 |
| Gastrointestinal disease | 2.61% (1.61-4.22) | 88.0% | < 0.0001 | 7 | 5038 |
| Peptic ulcer | 2.07% (0.67-6.22) | NR | NR | 1 | 145 |
| Gout | 1.12% (0.36-3.40) | 0.0% | 0.5651 | 2 | 269 |
| Hyperlipidemia | 2.79% (1.42-5.40) | 92.1% | < 0.0001 | 9 | 4379 |
| hyperuricemia | 2.33% (0.88-6.03) | NR | NR | 1 | 172 |
| Thyroid disease | 2.13% (1.55-2.92) | 0.0% | 0.7496 | 8 | 1739 |
| Autoimmune disease | 1.38% (1.02-1.87) | 0.0% | 0.7783 | 11 | 4195 |
| Blood system diseases | 2.22% (1.43-3.45) | 70.7% | 0.0168 | 4 | 3114 |
| Bone disease | 3.36% (1.69-6.58) | NR | NR | 1 | 238 |
| Genital system diseases | 5.72% (3.66-8.82) | 66.8% | 0.0827 | 2 | 546 |
| Benign prostatic hyperplasia | 1.05% (0.74-1.48) | NR | NR | 1 | 3044 |
| Prostatitis | 0.13% (0.05-0.35) | NR | NR | 1 | 3044 |
| Gynecological disease | 2.94% (1.41-6.04) | NR | NR | 1 | 238 |
| HIV infection | 0.64% (0.36-1.13) | 0.0% | 0.999 | 11 | 1872 |
| Nervous system disease | 4.05% (3.08-5.29) | 57.0% | 0.0228 | 8 | 3088 |
| Rheumatism | 1.83% (0.76-4.32) | 0.0% | 0.8884 | 2 | 273 |
| Urinary system disease | 2.88% (2.04-4.07) | 0.0% | 0.482 | 2 | 1075 |
| Urinary tract infection | 2.09% (0.87-4.93) | NR | NR | 1 | 239 |
| Urolithiasis | 2.14% (0.69-6.43) | NR | NR | 1 | 140 |
| **Complications** |  |  |  |  |  |
| Acute cardiac injury | 10.81% (7.64-15.07) | 97.3% | < 0.0001 | 29 | 14176 |
| Acute coronary syndrome | 2.15% (1.20-3.84) | NR | NR | 1 | 511 |
| Arrhythmia | 14.96% (9.97-21.83) | 89.6% | < 0.0001 | 10 | 1739 |
| Atrial fibrillation | 2.38% (0.77-7.12) | NR | NR | 1 | 126 |
| Cardiac insufficiency | 0.91% (0.58-1.43) | NR | NR | 1 | 2079 |
| Cardiovascular injury | 5.93% (3.61-9.60) | NR | NR | 1 | 253 |
| Heart failure | 8.26% (3.82-16.94) | 97.2% | < 0.0001 | 12 | 7400 |
| Hypoxic encephalopathy | 8.76% (5.94-12.74) | NR | NR | 1 | 274 |
| Myocardial infarction | 0.77% (0.35-1.71) | 0.0% | 0.5549 | 3 | 778 |
| Myocardial injury | 18.32% (12.05-26.86) | 90.1% | < 0.0001 | 7 | 1534 |
| Stroke | 1.74% (0.92-3.29) | 38.2% | 0.198 | 3 | 572 |
| Acquired pneumonia | 10.31% (6.75-15.44) | NR | NR | 1 | 194 |
| ARDS | 13.25% (9.08-18.94) | 98.2% | 0 | 41 | 19666 |
| Lung injury | 3.16% (1.59-6.19) | NR | NR | 1 | 253 |
| Respiratory failure | 19.98% (10.40-34.95) | 99.1% | 0 | 16 | 13145 |
| Respiratory injury | 14.10% (3.78-40.65) | 97.8% | < 0.0001 | 2 | 576 |
| Abnormal kidney function | 9.74% (7.35-12.80) | NR | NR | 1 | 462 |
| Acute kidney injury | 8.06% (6.20-10.41) | 95.2% | < 0.0001 | 40 | 16009 |
| kidney failure | 6.67% (1.12-31.01) | 95.4% | < 0.0001 | 2 | 328 |
| Liver injury | 20.68% (12.94-31.40) | 98.7% | < 0.0001 | 21 | 9839 |
| Gastrointestinal bleeding | 1.15% (0.68-1.94) | 71.1% | 0.004 | 6 | 4675 |
| Acidosis | 10.87% (7.14-16.19) | 67.5% | 0.0264 | 4 | 468 |
| Alkalosis | 6.33% (0.45-50.33) | 91.8% | 0.0005 | 2 | 163 |
| Electrolyte disturbance | 1.54% (1.12-2.11) | 0.0% | 0.4109 | 3 | 2472 |
| Hyperglycemia | 33.21% (29.39-37.27) | NR | NR | 1 | 548 |
| Hyperkalaemia | 23.36% (18.72-28.73) | NR | NR | 1 | 274 |
| Hypokalemia | 3.56% (1.86-6.69) | NR | NR | 1 | 253 |
| Hypoproteinaemia | 25.68% (11.63-47.56) | 96.2% | < 0.0001 | 3 | 2336 |
| Bacteremia | 6.27% (4.11-9.45) | 68.4% | 0.075 | 2 | 787 |
| Bacterial infection | 13.27% (9.71-17.88) | 92.4% | < 0.0001 | 8 | 2870 |
| Fungi infection | 3.28% (1.64-6.44) | 77.3% | 0.0041 | 4 | 1016 |
| Secondary infection | 8.83% (4.36-17.06) | 97.2% | < 0.0001 | 10 | 4103 |
| Sepsis | 17.65% (6.74-38.88) | 98.3% | < 0.0001 | 12 | 6842 |
| Septic shock | 5.45% (2.99-9.71) | 96.8% | < 0.0001 | 13 | 9072 |
| Anemia | 16.93% (3.07-56.75) | 98.3% | < 0.0001 | 2 | 354 |
| Coagulopathy | 21.29% (10.39-38.70) | 98.6% | < 0.0001 | 8 | 4572 |
| DIC | 2.22% (0.79-6.09) | 91.5% | < 0.0001 | 8 | 4190 |
| Thrombocytopenia | 1.06% (0.48-2.36) | 88.0% | 0.0039 | 2 | 3476 |
| Thrombus | 0.39% (0.23-0.68) | 73.8% | 0.0508 | 2 | 3297 |
| Multiple organ dysfunction syndrome | 8.04% (1.97-27.54) | 97.3% | < 0.0001 | 5 | 1071 |
| Multiple organ failure | 2.44% (1.10-5.34) | 95.1% | < 0.0001 | 3 | 3670 |
| Shock | 4.73% (2.88-7.69) | 93.3% | < 0.0001 | 24 | 7429 |
| Anaphylaxis | 2.37% (1.07-5.18) | NR | NR | 1 | 253 |
| Pneumonthorax | 1.02% (0.38-2.69) | 27.1% | 0.2415 | 2 | 392 |
| Rhabdomyolysis | 9.65% (5.42-16.59) | NR | NR | 1 | 114 |

NR: not reporTable. I² values and p value were NR, only one study was available. 95%CI: 95% confidence interval. ARDS: acute respiratory distress syndrome, COPD: chronic obstructive pulmonary disease, DIC: Disseminated intravascular coagulation, MODS: Multiple organ dysfunction syndrome

# Supplementary Table 4 Subgroup of gender ratio for comorbidities and complications of COVID-19 in total

|  | **OR (95% CI)** | **I²** | **p value** | **Number of studies** | **Number of participants** | **OR (95% CI)** | **I²** | **p value** | **Number of studies** | **Number of participants** | **p value between subgroup** |
| --- | --- | --- | --- | --- | --- | --- | --- | --- | --- | --- | --- |
| **Comorbidities** | **Female>Male** |  |  |  |  | **Male>Female** |  |  |  |  |  |
| Hypertension | 25.51% (23.15-28.02) | 92.8% | < 0.01 | 71 | 28172 | 28.21% (25.57-31.01) | 95.4% | 0 | 100 | 39869 | 0.1457 |
| Diabetes | 11.79% (10.60-13.09) | 88.4% | < 0.01 | 72 | 29112 | 13.43% (12.25-14.70) | 89.2% | < 0.01 | 104 | 41403 | 0.067 |
| Malignancy | 2.91% (2.46-3.45) | 68.8% | < 0.01 | 51 | 23940 | 2.87% (2.44-3.37) | 76.9% | < 0.01 | 69 | 28856 | 0.8952 |
| Cardiovascular diseases | 9.05% (6.95-11.70) | 94.8% | < 0.01 | 35 | 14349 | 10.44% (7.64-14.12) | 96.1% | < 0.01 | 32 | 11622 | 0.486 |
| Arrhythmia | 3.25% (1.04-9.72) | 90.9% | < 0.01 | 3 | 922 | 7.11% (2.86-16.57) | 85.1% | < 0.01 | 2 | 275 | 0.2836 |
| Chronic heart disease | 5.37% (3.43-8.32) | 83.3% | < 0.01 | 6 | 1345 | 7.07% (4.45-11.06) | 93.2% | < 0.01 | 10 | 5104 | 0.3971 |
| Coronary artery disease | 5.28% (2.67-10.14) | 90.7% | < 0.01 | 2 | 1317 | 12.10% (8.26-17.40) | 77.9% | 0.01 | 3 | 991 | 0.0314 |
| Coronary heart disease | 7.04% (5.79-8.54) | 79.8% | < 0.01 | 23 | 11595 | 8.12% (6.62-9.92) | 89.4% | < 0.01 | 42 | 16561 | 0.3189 |
| Cerebrovascular disease | 4.13% (3.25-5.23) | 82.1% | < 0.01 | 32 | 12907 | 4.26% (3.54-5.11) | 77.0% | < 0.01 | 40 | 14642 | 0.8405 |
| Cerebral infarction | 3.20% (2.52-4.06) | 0.0% | 0.56 | 2 | 2060 | 4.25% (3.21-5.61) | 39.4% | 0.19 | 3 | 1268 | 0.1324 |
| Stroke | 3.79% (2.56-5.57) | 58.7% | 0.09 | 3 | 2418 | 2.22% (1.21-4.04) | 55.8% | 0.06 | 5 | 1975 | 0.1437 |
| Chronic bronchitis | 4.69% (1.82-11.54) | 77.2% | 0.04 | 2 | 253 | 1.89% (0.77-4.54) | 92.9% | < 0.01 | 3 | 3147 | 0.1671 |
| Chronic lung disease | 4.71% (3.58-6.18) | 57.8% | 0.01 | 10 | 3696 | 5.27% (3.79-7.29) | 87.6% | < 0.01 | 20 | 9258 | 0.6056 |
| COPD | 2.64% (2.05-3.39) | 85.6% | < 0.01 | 41 | 19784 | 3.44% (2.77-4.26) | 88.2% | < 0.01 | 52 | 21497 | 0.1162 |
| Respiratory disease | 6.36% (4.72-8.51) | 85.1% | < 0.01 | 10 | 4393 | 4.91% (3.48-6.90) | 82.8% | < 0.01 | 11 | 3705 | 0.2639 |
| Tuberculosis | 1.14% (0.80-1.62) | 35.3% | 0.2 | 4 | 3470 | 1.37% (0.97-1.94) | 0.0% | 0.93 | 5 | 2261 | 0.4598 |
| Chronic kidney disease | 2.66% (2.09-3.39) | 81.2% | < 0.01 | 34 | 15318 | 2.60% (2.06-3.28) | 87.2% | < 0.01 | 54 | 22228 | 0.8888 |
| renal insufficiency | 1.18% (0.48-2.89) | 80.6% | < 0.01 | 5 | 2530 | 1.88% (1.00-3.50) | 86.2% | < 0.01 | 4 | 3244 | 0.4066 |
| Chronic liver disease | 3.42% (2.61-4.46) | 88.6% | < 0.01 | 38 | 15804 | 3.78% (3.06-4.67) | 86.6% | < 0.01 | 37 | 12499 | 0.5631 |
| Fatty liver | 7.35% (3.40-15.18) | 81.7% | < 0.01 | 3 | 681 | 10.39% (6.32-16.62) | 80.3% | 0.02 | 2 | 415 | 0.4458 |
| Hepatitis B | 2.12% (1.16-3.87) | 84.7% | < 0.01 | 5 | 2729 | 2.04% (0.96-4.29) | 74.1% | 0.02 | 3 | 987 | 0.938 |
| Gastrointestinal disease | 3.71% (2.50-5.47) | 53.4% | 0.09 | 4 | 1580 | 1.75% (0.81-3.73) | 84.1% | < 0.01 | 3 | 3458 | 0.0854 |
| Hyperlipidemia | 4.12% (2.94-5.74) | 7.1% | 0.36 | 4 | 801 | 2.32% (0.73-7.11) | 95.7% | < 0.01 | 5 | 3578 | 0.3405 |
| Thyroid disease | 2.14% (1.34-3.42) | 0.0% | 0.68 | 3 | 793 | 2.11% (1.37-3.25) | 0.0% | 0.48 | 5 | 946 | 0.966 |
| Autoimmune disease | 1.12% (0.70-1.80) | 0.0% | 0.95 | 4 | 1513 | 1.57% (1.16-2.11) | 0.0% | 0.64 | 7 | 2682 | 0.2448 |
| HIV infection | 0.67% (0.30-1.48) | 0.0% | 0.99 | 5 | 900 | 0.62% (0.28-1.37) | 0.0% | 0.99 | 6 | 972 | 0.8936 |
| Nervous system disease | 4.99% (3.82-6.50) | 61.3% | 0.05 | 4 | 2130 | 2.82% (1.94-4.08) | 0.0% | 0.74 | 4 | 958 | 0.0139 |
| **Complications** | **Female>Male** |  |  |  |  | **Male>Female** |  |  |  |  |  |
| Acute cardiac injury | 9.84% (5.68-16.51) | 95.4% | < 0.01 | 9 | 4382 | 11.30% (7.31-17.09) | 97.8% | < 0.01 | 20 | 9794 | 0.6902 |
| Arrhythmia | 11.03% (6.22-18.82) | 89.1% | < 0.01 | 6 | 1062 | 23.05% (17.44-29.82) | 76.8% | < 0.01 | 4 | 677 | 0.0158 |
| Heart failure | 5.19% (2.15-11.97) | 91.6% | < 0.01 | 4 | 3124 | 10.47% (3.79-25.77) | 97.5% | < 0.01 | 8 | 4276 | 0.2936 |
| Myocardial injury | 14.92% (8.31-25.34) | 90.0% | < 0.01 | 3 | 454 | 21.17% (12.29-33.98) | 91.5% | < 0.01 | 4 | 1080 | 0.3677 |
| ARDS | 11.29% (6.98-17.77) | 95.8% | < 0.01 | 15 | 5427 | 14.56% (8.62-23.54) | 98.7% | 0 | 26 | 14239 | 0.4716 |
| Respiratory failure | 18.06% (7.46-37.58) | 98.3% | < 0.01 | 5 | 3621 | 20.91% (8.74-42.19) | 99.3% | < 0.01 | 11 | 9524 | 0.8029 |
| Acute kidney injury | 7.31% (4.84-10.91) | 88.2% | < 0.01 | 12 | 4431 | 8.42% (6.07-11.58) | 96.2% | < 0.01 | 28 | 11578 | 0.5942 |
| Liver injury | 22.06% (13.04-34.83) | 96.7% | < 0.01 | 7 | 3593 | 19.97% (10.21-35.38) | 99% | < 0.01 | 14 | 6246 | 0.8066 |
| Gastrointestinal bleeding | 1.85% (0.96-3.52) | 58.8% | 0.09 | 3 | 873 | 0.85% (0.49-1.47) | 66.7% | 0.05 | 3 | 3802 | 0.0733 |
| Bacterial infection | 15.07% (12.22-18.44) | NR | NR | 1 | 511 | 12.88% (8.94-18.21) | 92.4% | < 0.01 | 7 | 2359 | 0.4522 |
| Secondary infection | 4.92% (1.49-15.01) | 97.0% | < 0.01 | 3 | 2347 | 11.26% (5.09-23.10) | 96.7% | < 0.01 | 7 | 1756 | 0.2406 |
| Sepsis | 31.11% (10.09-64.49) | 98.8% | < 0.01 | 4 | 3077 | 12.75% (3.43-37.54) | 98.2% | < 0.01 | 8 | 3765 | 0.2651 |
| Septic shock | 6.22% (1.52-22.13) | 96.0% | < 0.01 | 5 | 3206 | 4.84% (2.92-7.93) | 94.1% | < 0.01 | 8 | 5866 | 0.7377 |
| Coagulopathy | 20.47% (9.21-39.49) | 97.7% | < 0.01 | 2 | 2167 | 21.46% (8.43-44.79) | 98.7% | < 0.01 | 6 | 2405 | 0.9342 |
| DIC | 1.26% (0.72-2.21) | 0.0% | 0.94 | 3 | 950 | 3.13% (0.68-13.24) | 92.0% | < 0.01 | 5 | 3240 | 0.2708 |
| MODS | 2.74% (1.83-4.09) | 0.0% | 0.59 | 3 | 840 | 38.10% (32.06-44.52) | 0.0% | 0.42 | 2 | 231 | < 0.0001 |
| Shock | 2.75% (1.33-5.58) | 83.7% | < 0.01 | 9 | 2011 | 6.53% (3.59-11.59) | 94.1% | < 0.01 | 15 | 5418 | 0.0670 |

ND: No data available. NR:not reporTable. I² values and p value were NR, only one study was available. OR: odds ratio. 95%CI: 95% confidence interval. ARDS: acute respiratory distress syndrome, COPD: chronic obstructive pulmonary disease, DIC: Disseminated intravascular coagulation, MODS: Multiple organ dysfunction syndrome

# Supplementary Table 5 Pooled estimate prevalence of comorbidities and complications for COVID-19 in mild, moderate, severe, and critical group

|  | **Pooled estimate prevalence (95% CI)** | **P/S** | **Pooled estimate prevalence (95% CI)** | **P/S** | **Pooled estimate prevalence (95% CI)** | **P/S** | **Pooled estimate prevalence (95% CI)** | **P/S** |
| --- | --- | --- | --- | --- | --- | --- | --- | --- |
| **Comorbidities** | **Mild** |  | **Moderate** |  | **Severe** |  | **Critical** |  |
| Hypertension | 20.37% (15.28-26.63) | 3118/15 | 19.29% (16.17-22.85) | 7811/27 | 34.72% (31.48-38.10) | 7914/41 | 43.94% (38.94-49.06) | 2031/31 |
| Diabetes | 7.84% (5.78-10.54) | 3048/15 | 8.59% (7.25-10.16) | 8258/30 | 17.99% (16.29-19.84) | 8524/44 | 22.68% (19.93-25.69) | 2162/33 |
| Malignancy | 1.70% (1.05-2.74) | 3219/14 | 1.80% (1.31-2.45) | 6296/18 | 3.47% (2.74-4.38) | 7295/30 | 4.38% (2.72-6.97) | 1697/22 |
| Cardiovascular diseases | 2.57% (0.61-10.15) | 783/8 | 6.17% (3.40-10.93) | 1723/11 | 17.48% (12.32-24.22) | 1940/18 | 28.24% (18.32-40.85) | 535/11 |
| Cardiac insufficiency | 0.00% (0.00-100.00) | 25/1 | 0.42% (0.19-0.93) | 1435/1 | 3.32% (1.67-6.50) | 241/1 | 12.12% (6.18-22.41) | 66/1 |
| Chronic heart disease | ND | ND/ND | 6.38% (2.07-18.00) | 47/1 | 30.56% (17.80-47.21) | 36/1 | 44.44% (24.00-66.96) | 18/1 |
| Coronary artery disease | ND | ND/ND | 4.92% (3.12-7.67) | 366/1 | 13.18% (7.28-22.69) | 514/2 | 14.47% (9.81-20.83) | 159/2 |
| Coronary atherosclerosis | 3.41% (2.59-4.47) | 1467/1 | ND | ND/ND | 6.91% (5.70-8.35) | 1418/1 | 10.69% (6.75-16.53) | 159/1 |
| Coronary heart disease | 3.34% (2.33-4.76) | 869/5 | 3.97% (3.02-5.21) | 5465/14 | 8.90% (6.65-11.82) | 4167/16 | 13.26% (10.68-16.35) | 1027/12 |
| Myocardial infarction | ND | ND/ND | ND | ND/ND | 12.65% (8.40-18.62) | 166/1 | 21.58% (15.52-29.19) | 139/1 |
| Cerebrovascular disease | 2.01% (1.26-3.22) | 844/6 | 2.24% (1.62-3.09) | 1608/11 | 5.27% (3.48-7.90) | 1844/16 | 12.61% (9.05-17.30) | 607/13 |
| Cerebral infarction | 3.57% (1.16-10.49) | 84/2 | 1.95% (1.38-2.74) | 1642/2 | 8.31% (5.91-11.58) | 373/3 | 9.09% (4.40-17.86) | 77/2 |
| Intracerebral hemorrhage | 0.00% (0.00-100.00) | 25/1 | 0.42% (0.19-0.93) | 1435/1 | 1.66% (0.62-4.34) | 241/1 | 0.00% (0.00-100.00) | 66/1 |
| Stroke | ND | ND/ND | 1.23% (0.62-2.44) | 651/2 | 3.80% (2.41-5.95) | 474/2 | 12.03% (7.80-18.08) | 158/2 |
| Asthma | 0.20% (0.06-0.62) | 1492/2 | 0.63% (0.33-1.20) | 1435/1 | 0.48% (0.16-1.40) | 1659/2 | 0.44% (0.06-3.08) | 225/2 |
| Chronic bronchitis | ND | ND/ND | 1.11% (0.66-1.86) | 1263/1 | 1.05% (0.58-1.89) | 1044/1 | 4.76% (1.80-12.01) | 84/1 |
| Chronic lung disease | 0.82% (0.09-6.86) | 210/5 | 3.86% (2.27-6.49) | 694/5 | 4.96% (2.03-11.61) | 515/7 | 12.68% (4.05-33.28) | 214/5 |
| COPD | 0.45% (0.18-1.09) | 2454/7 | 1.30% (0.75-2.27) | 4578/14 | 3.57% (2.47-5.13) | 5162/22 | 5.79% (3.90-8.51) | 1373/17 |
| Respiratory disease | 2.46% (1.34-4.48) | 609/3 | 3.08% (2.03-4.64) | 882/4 | 6.81% (5.13-9.00) | 1680/9 | 12.21% (9.42-15.67) | 426/7 |
| Tuberculosis | 0.00% (0.00-100.00) | 25/1 | 0.09% (0.00-9.31) | 1801/2 | 2.40% (0.94-5.96) | 652/2 | 4.21% (2.12-8.19) | 190/2 |
| Chronic kidney disease | 0.38% (0.12-1.18) | 785/11 | 0.72% (0.43-1.21) | 3485/15 | 2.98% (1.94-4.54) | 3289/23 | 7.14% (4.17-11.95) | 1137/18 |
| Nephritis | 0.20% (0.07-0.63) | 1467/1 | ND | ND/ND | 0.21% (0.07-0.65) | 1418/1 | 0.63% (0.09-4.33) | 159/1 |
| Chronic liver disease | 1.57% (0.85-2.90) | 635/8 | 2.83% (1.78-4.49) | 2322/11 | 3.28% (1.89-5.63) | 2081/15 | 5.63% (3.00-10.30) | 540/9 |
| Cirrhosis | 0.27% (0.10-0.71) | 1492/2 | 0.35% (0.15-0.83) | 1435/1 | 0.98% (0.43-2.22) | 1805/3 | 0.40% (0.06-2.77) | 251/3 |
| Fatty liver | ND | ND/ND | ND | ND/ND | ND | ND/ND | ND | ND/ND |
| Hepatitis B | 0.00% (0.00-100.00) | 25/1 | 1.05% (0.63-1.73) | 1435/1 | 1.24% (0.40-3.79) | 241/1 | 0.00% (0.00-100.00) | 66/1 |
| Gastrointestinal disease | 1.16% (0.72-1.86) | 1467/1 | 0.48% (0.07-3.35) | 207/1 | 2.79% (1.23-6.17) | 1819/3 | 3.00% (0.95-9.02) | 253/2 |
| Hyperlipidemia | 0.75% (0.42-1.35) | 1467/1 | 3.57% (0.89-13.19) | 56/1 | 0.83% (0.47-1.45) | 1454/2 | 0.68% (0.01-33.06) | 187/2 |
| hyperuricemia | 0.89% (0.13-6.06) | 112/1 | ND | ND/ND | 5.00% (1.62-14.39) | 60/1 | ND | ND/ND |
| Thyroid disease | 3.70% (0.52-22.08) | 27/1 | 1.91% (0.72-4.99) | 209/1 | 0.00% (0.00-100.00) | 31/1 | 0.00% (0.00-100.00) | 5/1 |
| Autoimmune disease | 0.00% (0.00-100.00) | 10/1 | 0.71% (0.34-1.48) | 984/4 | 0.99% (0.44-2.18) | 609/4 | 3.90% (1.59-9.24) | 208/3 |
| Blood system diseases | 1.17% (0.17-7.80) | 423/2 | ND | ND/ND | 4.96% (2.90-8.36) | 262/1 | ND | ND/ND |
| Genital system diseases | ND | ND/ND | 5.31% (2.97-9.34) | 207/1 | 12.79% (7.23-21.64) | 86/1 | ND | ND/ND |
| Benign prostatic hyperplasia | 0.41% (0.18-0.91) | 1467/1 | ND | ND/ND | 1.55% (1.02-2.34) | 1418/1 | 2.52% (0.95-6.51) | 159/1 |
| Prostatitis | 0.07% (0.01-0.48) | 1467/1 | ND | ND/ND | 0.21% (0.07-0.65) | 1418/1 | 0.00% (0.00-100.00) | 159/1 |
| HIV infection | 0.69% (0.10-4.73) | 145/2 | 0.00% (0.00-100.00) | 56/1 | 0.00% (0.00-100.00) | 85/2 | 8.33% (1.16-41.32) | 12/1 |
| Nervous system disease | 1.35% (0.64-2.80) | 519/2 | 2.30% (1.10-4.73) | 305/2 | 4.29% (2.85-6.42) | 1064/5 | 3.66% (1.65-7.90) | 164/2 |
| Rheumatism | 2.68% (0.87-7.98) | 112/1 | 0.00% (0.00-100.00) | 47/1 | 1.04% (0.15-7.02) | 96/2 | 5.56% (0.78-30.65) | 18/1 |
| Urinary system disease | ND | ND/ND | 4.50% (1.89-10.37) | 111/1 | 2.46% (1.46-4.11) | 569/2 | 4.96% (2.39-10.05) | 141/2 |
| **Complications** | **Mild** |  | **Moderate** |  | **Severe** |  | **Critical** |  |
| Acute cardiac injury | 0.43% (0.00-57.19) | 428/2 | 3.97% (2.98-5.27) | 1134/2 | 7.22% (4.93-10.45) | 1173/5 | 43.43% (15.87-75.76) | 312/3 |
| Atrial fibrillation | 0.00% (0.00-100.00) | 33/1 | 0.00% (0.00-100.00) | 56/1 | 4.00% (0.56-23.55) | 25/1 | 16.67% (4.20-47.72) | 12/1 |
| Heart failure | 0.14% (0.03-0.54) | 1467/1 | 0.53% (0.24-1.17) | 1133/2 | 1.85% (0.29-10.77) | 2142/3 | 17.18% (4.37-48.47) | 444/3 |
| ARDS | 0.03% (0.00-2.92) | 1905/4 | 0.03% (0.00-2.92) | 1905/4 | 4.86% (4.61-5.12) | 2615/7 | 94.17% (20.78-99.90) | 485/5 |
| Respiratory failure | 0.00% (0.00-100.00) | 1800/2 | 0.44% (0.18-1.05) | 1134/2 | 8.70% (1.15-43.87) | 2405/4 | 90.69% (28.08-99.59) | 445/3 |
| Abnormal kidney function | ND | ND/ND | 8.71% (6.32-11.89) | 402/1 | 10.26% (3.90-24.33) | 39/1 | 28.57% (13.43-50.76) | 21/1 |
| Acute kidney injury | 3.81% (1.44-9.71) | 105/2 | 2.14% (0.88-5.12) | 1950/6 | 6.23% (3.10-12.15) | 1206/8 | 39.11% (29.42-49.74) | 403/6 |
| Liver injury | 10.00% (1.39-46.72) | 10/1 | 23.04% (14.20-35.13) | 1233/2 | 43.48% (39.88-47.15) | 713/2 | 52.84% (47.00-58.60) | 282/2 |
| Bacterial infection | 2.50% (0.63-9.45) | 80/1 | ND | ND/ND | 8.89% (3.38-21.41) | 45/1 | 27.42% (17.78-39.76) | 62/1 |
| Fungi infection | 0.00% (0.00-100.00) | 80/1 | ND | ND/ND | 0.00% (0.00-100.00) | 45/1 | 3.23% (0.81-12.00) | 62/1 |
| Secondary infection | 0.00% (0.00-100.00) | 95/1 | 1.34% (0.02-45.10) | 1134/2 | 10.02% (1.12-52.27) | 911/4 | 31.32% (4.26-82.39) | 312/3 |
| Septic shock | 0.00% (0.00-100.00) | 10/1 | 0.31% (0.12-0.83) | 1280/3 | 1.12% (0.60-2.06) | 895/4 | 53.51% (24.36-80.44) | 326/4 |
| Coagulopathy | ND | ND/ND | 3.97% (2.98-5.27) | 1134/2 | 21.23% (3.76-65.00) | 725/2 | 59.09% (53.30-64.64) | 286/2 |
| Thrombocytopenia | 0.34% (0.14-0.82) | 1467/1 | ND | ND/ND | 0.78% (0.43-1.40) | 1418/1 | 2.52% (0.95-6.51) | 159/1 |
| Thrombus | 0.20% (0.07-0.63) | 1467/1 | ND | ND/ND | 0.35% (0.15-0.84) | 1418/1 | 1.26% (0.31-4.89) | 159/1 |
| Shock | 0.00% (0.00-100.00) | 428/2 | ND | ND/ND | 1.31% (0.05-25.51) | 448/3 | 61.54% (42.07-77.90) | 26/1 |

ND: No data available. P/S: the number of participants/ the number of included studies. 95%CI: 95% confidence interval.COPD: chronic obstructive pulmonary disease, ARDS: acute respiratory distress syndrome.

# Supplementary Table 6 Subgroup of gender ratio for comorbidities and complications of COVID-19 in mild/moderate versus severe/critical

|  | **OR (95% CI)** | **I²** | **p value of Heterogeneity** | **Number of studies** | **Number of participants** | **OR (95% CI)** | **I²** | **p value of Heterogeneity** | **Number of studies** | **Number of participants** | **p value between subgroup** |
| --- | --- | --- | --- | --- | --- | --- | --- | --- | --- | --- | --- |
| **Comorbidities** | **Female>Male** |  |  |  |  | **Male>Female** |  |  |  |  |  |
|  |  |  |  |  |  |  |  |  |  |  |  |
| Hypertension | 2.81 (2.41-3.26) | 72.3% | < 0.01 | 45 | 19304 | 2.55 (2.19-2.97) | 70.7% | < 0.01 | 50 | 20438 | 0.3844 |
| Diabetes | 2.36 (2.04-2.74) | 45.9% | < 0.01 | 46 | 19670 | 2.54 (2.17-2.97) | 55.9% | < 0.01 | 51 | 20740 | 0.5045 |
| Malignancy | 2.14 (1.74-2.63) | 0.0% | 0.47 | 30 | 16244 | 2.28 (1.80-2.90) | 0.0% | 0.74 | 34 | 14720 | 0.6761 |
| Cardiovascular diseases | 2.85 (2.30-3.53) | 41.5% | 0.02 | 22 | 9257 | 3.04 (2.19-4.22) | 50.8% | 0.01 | 15 | 4075 | 0.7382 |
| Chronic heart disease | 4.79 (2.30-9.96) | 41.1% | 0.15 | 5 | 1130 | 9.68 (5.34-17.55) | 0.0% | 0.72 | 4 | 2453 | 0.1433 |
| Coronary heart disease | 2.30 (1.79-2.94) | 18.8% | 0.26 | 12 | 7668 | 3.29 (2.34-4.62) | 55.6% | < 0.01 | 21 | 8857 | 0.0934 |
| Cerebrovascular disease | 3.35 (2.42-4.64) | 33.8% | 0.08 | 19 | 8552 | 2.42 (1.59-3.69) | 34.1% | 0.07 | 20 | 6030 | 0.2307 |
| Chronic lung disease | 1.25 (0.56-2.75) | 16.1% | 0.31 | 5 | 1001 | 2.68 (1.32-5.46) | 55.9% | 0.02 | 9 | 2701 | 0.1582 |
| COPD | 3.18 (2.47-4.08) | 11.9% | 0.3 | 24 | 14033 | 2.79 (2.13-3.67) | 1.1% | 0.45 | 25 | 10758 | 0.4966 |
| Respiratory disease | 2.41 (1.73-3.36) | 0.0% | 0.62 | 8 | 3663 | 2.75 (1.57-4.82) | 41.5% | 0.1 | 8 | 2347 | 0.6922 |
| Tuberculosis | 7.50 (0.46-122.37) | 81.2% | < 0.01 | 3 | 3054 | 1.06 (0.38-2.93) | 0.0% | 0.56 | 4 | 1071 | 0.1968 |
| Chronic kidney disease | 5.14 (3.19-8.26) | 38.6% | 0.04 | 19 | 10266 | 2.57 (1.76-3.76) | 22.8% | 0.16 | 24 | 9837 | 0.0258 |
| renal insufficiency | 19.07 (6.52-55.79) | 0.0% | 0.99 | 4 | 2756 | 12.27 (1.47-102.53) | 0.0% | 0.64 | 2 | 241 | 0.7163 |
| Chronic liver disease | 1.72 (1.23-2.40) | 40.6% | 0.02 | 23 | 10821 | 1.05 (0.76-1.47) | 7.5% | 0.36 | 21 | 6961 | 0.0404 |
| Hepatitis B | 1.97 (0.79-4.93) | 39.4% | 0.18 | 4 | 2594 | 0.65 (0.14-3.04) | 0.0% | 0.91 | 2 | 713 | 0.2257 |
| Gastrointestinal disease | 1.57 (0.83-2.97) | 15.0% | 0.32 | 4 | 1580 | 0.96 (0.52-1.76) | 0.0% | 0.49 | 2 | 3184 | 0.274 |
| Hyperlipidemia | 1.49 (0.70-3.19) | 0.0% | 0.56 | 4 | 801 | 1.00 (0.49-2.05) | 0.0% | 0.38 | 3 | 3329 | 0.4528 |
| Thyroid disease | 0.42 (0.11-1.67) | 0.0% | 0.81 | 2 | 658 | 6.28 (1.38-28.47) | 0.0% | 0.38 | 3 | 467 | 0.0096 |
| Autoimmune disease | 1.34 (0.47-3.85) | 0.0% | 0.63 | 3 | 1362 | 4.76 (1.44-15.77) | 0.0% | 0.67 | 4 | 1010 | 0.1196 |
| HIV infection | 3.14 (0.64-15.40) | 0.0% | 0.77 | 4 | 570 | 0.92 (0.14-5.90) | 0.0% | 0.84 | 3 | 529 | 0.3262 |
| Nervous system disease | 3.29 (0.60-18.00) | 78.6% | 0.03 | 2 | 1359 | 1.34 (0.51-3.50) | 18.4% | 0.29 | 3 | 844 | 0.3679 |
| **Complications** | **Female>Male** |  |  |  |  | **Male>Female** |  |  |  |  |  |
| Acute cardiac injury | 15.79 (7.34-33.97) | 39.4% | 0.16 | 5 | 3322 | 5.74 (3.59-9.17) | 42.0% | 0.11 | 7 | 2784 | 0.0273 |
| Arrhythmia | 24.86 (9.99-61.88) | 16.4% | 0.31 | 4 | 686 | 6.43 (3.61-11.43) | NA | NA | 1 | 323 | 0.0139 |
| ARDS | 102.57 (28.08-374.66) | 61.2% | 0.01 | 8 | 3795 | 43.56 (22.04-86.06) | 38.2% | 0.1 | 10 | 5076 | 0.2514 |
| Respiratory failure | 86.09 (36.87-200.99) | 0.0% | 0.87 | 2 | 2639 | 222.65 (43.04-1151.96) | 0.0% | 0.81 | 3 | 3347 | 0.3139 |
| Acute kidney injury | 9.14 (4.11-20.33) | 56.0% | 0.03 | 7 | 3223 | 7.03 (3.90-12.66) | 65.4% | < 0.01 | 13 | 4269 | 0.6049 |
| Liver injury | 3.63 (1.71-7.72) | 83.5% | < 0.01 | 5 | 2862 | 2.75(1.42-5.31) | 60.9% | 0.05 | 4 | 1030 | 0.5860 |
| Secondary infection | 28.12 (8.18-96.64) | 0.0% | 0.92 | 2 | 2147 | 16.12 (7.81-33.26) | 0.0% | 0.78 | 4 | 660 | 0.4462 |
| Septic shock | 72.52 (23.07-227.95) | 8.2% | 0.3 | 2 | 2319 | 24.06 (4.51-128.47) | 0.0% | 0.7 | 3 | 618 | 0.2866 |
| Shock | 54.08 (14.44-202.48) | 0.0% | 0.64 | 5 | 881 | 23.20 (13.30-40.45) | 0.0% | 0.7 | 7 | 1361 | 0.2469 |

NR: not reporTable. I² values were NR, only one study was available. OR: odds ratio. 95%CI: 95% confidence interval. COPD: Chronic obstructive pulmonary disease, ARDS: Acute respiratory distress syndrome, DIC: Disseminated intravascular coagulation, MODS:Multiple organ dysfunction syndrome.

# Supplementary Table 7 Subgroup of gender ratio for comorbidities and complications of COVID-19 in ICU admission versus non- ICU admission

|  | **OR (95% CI)** | **I²** | **p value of Heterogeneity** | **Number of studies** | **Number of participants** | **OR (95% CI)** | **I²** | **p value of Heterogeneity** | **Number of studies** | **Number of participants** | **p value between subgroup** |
| --- | --- | --- | --- | --- | --- | --- | --- | --- | --- | --- | --- |
| **Comorbidities** | **Female>Male** |  |  |  |  | **Male>Female** |  |  |  |  |  |
| Hypertension | 2.35 (1.56-3.55) | 31.6% | 4 | 0.22 | 1842 | 2.28 (1.52-3.41) | 25.2% | 6 | 0.25 | 1161 | 0.9128 |
| Diabetes | 2.67 (1.05-6.79) | 77.0% | 4 | < 0.01 | 1842 | 1.82 (1.06-3.12) | 28.4% | 6 | 0.22 | 1161 | 0.4882 |
| Malignancy | 4.07 (2.10-7.89) | 0.0% | 2 | 0.53 | 1291 | 4.14 (0.65-26.24) | 69.7% | 3 | 0.04 | 452 | 0.9868 |
| Cardiovascular diseases | 2.85 (1.68-4.85) | 0.0% | 2 | 0.44 | 1226 | 3.95 (2.26-6.92) | 11.7% | 4 | 0.33 | 932 | 0.4082 |

OR: odds ratio. 95%CI: 95% confidence interval.

# Supplementary Table 8 Subgroup of gender ratio for comorbidities and complications of COVID-19 in progressive versus non-progressive

|  | **OR (95% CI)** | **I²** | **p value of Heterogeneity** | **Number of studies** | **Number of participants** | **OR (95% CI)** | **I²** | **p value of Heterogeneity** | **Number of studies** | **Number of participants** | **p value between subgroup** |
| --- | --- | --- | --- | --- | --- | --- | --- | --- | --- | --- | --- |
| **Comorbidities** | **Female>Male** |  |  |  |  | **Male>Female** |  |  |  |  |  |
| Hypertension | 3.04 (1.35-6.84) | 81.0% | 4 | < 0.01 | 1270 | 1.88 (1.37-2.59) | 49.7% | 10 | 0.04 | 4031 | 0.2826 |
| Diabetes | 3.04 (0.83-11.09) | 85.7% | 3 | < 0.01 | 905 | 1.63 (1.10-2.41) | 48.0% | 10 | 0.04 | 4031 | 0.3673 |
| Malignancy | 2.31 (0.95-5.64) | 0.0% | 4 | 0.43 | 1595 | 1.71 (1.01-2.92) | 0.0% | 7 | 0.44 | 2789 | 0.5728 |
| Cardiovascular diseases | 3.33 (2.15-5.16) | 17.2% | 4 | 0.3 | 1595 | 2.69 (0.89-8.09) | 53.6% | 2 | 0.14 | 1126 | 0.7236 |
| Cerebrovascular disease | 6.74 (0.59-76.91) | NR | 1 | NA | 365 | 2.93 (1.30-6.60) | 0.0% | 5 | 0.6 | 1222 | 0.5242 |
| COPD | 1.63 (0.59-4.46) | 0.0% | 3 | 0.85 | 814 | 2.27 (1.13-4.56) | 32.5% | 6 | 0.19 | 2633 | 0.5912 |
| Chronic kidney disease | 6.03 (1.59-22.82) | 0.0% | 2 | 0.53 | 821 | 1.78 (0.79-4.00) | 12.5% | 5 | 0.33 | 1873 | 0.1246 |
| Chronic liver disease | 3.65 (0.44-30.50) | 24.5% | 2 | 0.25 | 476 | 1.15 (0.60-2.19) | 0.0% | 4 | 0.71 | 1747 | 0.3059 |

NR: not reporTable. I² values were NR, only one study was available. OR: odds ratio. 95%CI: 95% confidence interval. COPD: Chronic obstructive pulmonary disease.

# Supplementary Table 9 Subgroup of gender ratio in mortality for COVID-19 with comorbidities and complications vs without comorbidities and complications

|  | **OR (95% CI)** | **I²** | **p value of Heterogeneity** | **Number of studies** | **Number of participants** | **OR (95% CI)** | **I²** | **p value of Heterogeneity** | **Number of studies** | **Number of participants** | **p value between subgroup** |
| --- | --- | --- | --- | --- | --- | --- | --- | --- | --- | --- | --- |
| **Comorbidities** | **Female>Male** |  |  |  |  | **Male>Female** |  |  |  |  |  |
| Hypertension | 2.31 (1.85-2.89) | 55.2% | < 0.01 | 21 | 7653 | 2.09 (1.79-0.36) | 59.7% | < 0.01 | 41 | 16584 | 0.4481 |
| Diabetes | 2.16 (1.78-2.62) | 22.8% | 0.16 | 22 | 8592 | 1.87 (1.56-0.43) | 59.3% | < 0.01 | 42 | 16903 | 0.2784 |
| Malignancy | 2.97 (2.14-4.11) | 0.0% | 0.8 | 19 | 7823 | 2.36 (1.78-0.29) | 14.4% | 0.25 | 29 | 11989 | 0.3012 |
| Cardiovascular diseases | 3.85 (2.74-5.40) | 44.7% | 0.05 | 11 | 5283 | 2.98 (2.14-0.37) | 45.2% | 0.04 | 12 | 5793 | 0.2859 |
| Chronic heart disease | 1.57 (0.42-5.90) | NR | NR | 1 | 226 | 2.47 (1.37-4.45) | 67.0% | < 0.01 | 6 | 2651 | 0.54 |
| Coronary heart disease | 2.88 (1.89-4.39) | 53.2% | 0.02 | 10 | 3589 | 2.38 (1.74-0.48) | 57.8% | < 0.01 | 18 | 6344 | 0.4698 |
| Cerebrovascular disease | 3.18 (1.94-5.21) | 46.6% | 0.03 | 13 | 4201 | 2.72 (1.99-3.71) | 23.6% | 0.18 | 17 | 8035 | 0.597 |
| Chronic lung disease | 2.10 (1.23-3.58) | 0.0% | 0.65 | 3 | 2039 | 2.36 (1.80-3.09) | 0.0% | 0.56 | 12 | 6905 | 0.701 |
| COPD | 3.48 (2.38-5.10) | 0.0% | 0.78 | 15 | 4945 | 2.78 (1.96-0.49) | 37.9% | 0.04 | 22 | 8841 | 0.3894 |
| Respiratory disease | 3.80 (1.94-7.46) | 54.4% | 0.09 | 4 | 1988 | 5.10 (2.14-12.13) | 0.0% | 0.67 | 2 | 346 | 0.6004 |
| Chronic kidney disease | 4.32 (2.93-6.38) | 10.6% | 0.34 | 13 | 4421 | 2.51 (1.72-3.66) | 43.7% | < 0.01 | 27 | 11662 | 0.0491 |
| Chronic liver disease | 1.67 (0.99-2.79) | 25.4% | 0.19 | 13 | 4697 | 1.29 (0.90-1.85) | 0.0% | 0.95 | 13 | 4981 | 0.4262 |
| **Complications** | **Female>Male** |  |  |  |  | **Male>Female** |  |  |  |  |  |
| Acute cardiac injury | 30.96 (8.64-110.98) | 78.7% | < 0.01 | 3 | 1039 | 23.70 (11.93-47.05) | 84.3% | < 0.01 | 12 | 6872 | 0.7176 |
| Heart failure | 9.02 (0.68-119.92) | 93.2% | < 0.01 | 2 | 667 | 10.70 (4.30-26.65) | 68.1% | 0.01 | 5 | 1015 | 0.9024 |
| ARDS | 66.79 (24.72-180.48) | 67.6% | < 0.01 | 7 | 2027 | 60.76 (24.39-151.37) | 91.1% | < 0.01 | 13 | 8807 | 0.8906 |
| Respiratory failure | 41.45 (15.45-111.22) | 72.0% | 0.01 | 4 | 1577 | 31.02 (13.89-69.26) | 79.1% | < 0.01 | 8 | 6177 | 0.6552 |
| Acute kidney injury | 10.14 (3.54-29.01) | 77.9% | < 0.01 | 4 | 1010 | 20.27 (8.75-46.98) | 86.5% | < 0.01 | 13 | 7057 | 0.313 |
| Liver injury | 1.62 (0.14-18.42) | 95.9% | < 0.01 | 2 | 731 | 3.91 (2.56-5.97) | 49.3% | 0.05 | 9 | 5102 | 0.4833 |
| Gastrointestinal bleeding | 41.81 (9.27-188.67) | 0.0% | 0.93 | 3 | 873 | 27.22 (10.26-72.21) | 24.3% | 0.27 | 3 | 3802 | 0.6392 |
| Bacterial infection | 10.88 (6.17-19.20) | NR | NR | 1 | 511 | 5.41 (1.36-21.63) | 90.5% | < 0.01 | 5 | 2083 | 0.3606 |
| Sepsis | 24.60 (1.98-306.25) | 87.7% | < 0.01 | 3 | 1033 | 43.32 (4.02-466.95) | 85.0% | < 0.01 | 7 | 3664 | 0.749 |
| Septic shock | 69.11 (13.32-358.59) | 58.3% | 0.09 | 3 | 887 | 116.07 (11.37-1184.46) | 87.6% | < 0.01 | 5 | 5248 | 0.7212 |
| Coagulopathy | 14.10 (5.25-37.82) | NR | NR | 1 | 123 | 13.40 (3.78-47.55) | 83.3% | < 0.01 | 4 | 1992 | 0.9508 |
| Shock | 88.32 (20.05-389.04) | 0.0% | 0.96 | 4 | 1125 | 205.11 (38.99-1079.12) | 76.0% | < 0.01 | 6 | 3805 | 0.4582 |

NR: not reporTable. I² values were NR, only one study was available. OR: odds ratio. 95%CI: 95% confidence interval. COPD: chronic obstructive pulmonary disease, ARDS: acute respiratory distress syndrome.

# Supplementary File 1 Protocol amendments

We adjusted the quality assessment during the research process, which the 9-item tool in the Joanna Briggs Institute (JBI) Prevalence Critical Appraisal Checklist is more suitable for our study. Because of our research is more focused on the prevalence of comorbidities and complications in disease severity, progression, and mortality, and JBI was the development of a critical appraisal tool for use in systematic reviews addressing questions of prevalence. Compared with the Newcastle-Ottawa Scale, we finally chose the JBI to assess.

# Supplementary File 2 The detailed strategies in English and Chinese language databases

**Four main English-language databases**

***PubMed:***

#1: ((((((((((((((((((((COVID-19[MeSH Terms]) OR (COVID-19[Title/Abstract])) OR (COVID 19[Title/Abstract])) OR (COVID 2019[Title/Abstract])) OR (Novel Coronavirus[Title/Abstract])) OR (Novel Coronavirus 2019[Title/Abstract])) OR (Corona Virus Disease 2019[Title/Abstract])) OR (Coronavirus Disease[Title/Abstract])) OR (Severe Acute Respiratory Syndrome Coronavirus[Title/Abstract])) OR (Severe Acute Respiratory Syndrome Coronavirus 2[Title/Abstract])) OR (Severe Acute Respiratory Syndrome Corona Virus 2[Title/Abstract])) OR (SARS-Cov-2[Title/Abstract])) OR (Corona Virus[Title/Abstract])) OR (Coronavirus[Title/Abstract])) OR (Corona Virinae 19[Title/Abstract])) OR (Corona Virinae 2019[Title/Abstract])) OR (Corona Virus 19[Title/Abstract])) OR (Corona Virus 2019[Title/Abstract])) OR (ncov19[Title/Abstract])) OR (ncov2019[Title/Abstract])) OR (2019-ncov[Title/Abstract])

Results: 220486

#2: ((((((((((("clinical features"[Title/Abstract]) OR ("clinical characteristics"[Title/Abstract])) OR ("epidemiological characteristics"[Title/Abstract])) OR ("clinical character"[Title/Abstract])) OR (comorbidities[Title/Abstract])) OR (complications[Title/Abstract])) OR (mortality[Title/Abstract])) OR (death[Title/Abstract])) OR ("underlying diseases"[Title/Abstract])) OR ("related diseases"[Title/Abstract])) OR ("basic diseases"[Title/Abstract])) OR ("clinical outcomes"[Title/Abstract])

Results: 2568932

#3: (China[Title/Abstract]) OR (Chinese[Title/Abstract])

Results: 422901

#4: = #1 AND #2 AND #3

Results (PubMed): 3813

***Embase:***

#1: 'covid 19'/exp OR 'covid 19':ab,ti OR 'covid 2019':ab,ti OR 'novel coronavirus':ab,ti OR 'novel coronavirus 2019':ab,ti OR 'corona virus disease 2019':ab,ti OR 'coronavirus disease':ab,ti OR 'severe acute respiratory syndrome coronavirus':ab,ti OR 'severe acute respiratory syndrome coronavirus 2':ab,ti OR 'severe acute respiratory syndrome corona virus 2':ab,ti OR 'sars cov 2':ab,ti OR 'corona virus':ab,ti OR coronavirus:ab,ti OR 'corona virinae 19':ab,ti OR 'corona virinae 2019':ab,ti OR 'corona virus 19':ab,ti OR 'corona virus 2019':ab,ti OR ncov19:ab,ti OR ncov2019:ab,ti OR '2019 ncov':ab,ti

Results: 232559

#2: 'clinical features':ab,ti OR 'clinical characteristics':ab,ti OR 'epidemiological characteristics':ab,ti OR 'clinical character':ab,ti OR comorbidities:ab,ti OR complications:ab,ti OR mortality:ab,ti OR death:ab,ti OR 'underlying diseases':ab,ti OR 'related diseases':ab,ti OR 'basic diseases':ab,ti OR 'clinical outcomes':ab,ti

Results: 3615977

#3: China:ab,ti OR Chinese:ab,ti

Results: 511874

#4: = #1 AND #2 AND #3

Results (Embase): 4059

***Cochrane Library：***

#1: (“COVID-19” or “COVID 19” or “COVID 2019” or “Novel Coronavirus” or “Novel Coronavirus 2019” or “Corona Virus Disease 2019” or “Coronavirus Disease” or “Severe Acute Respiratory Syndrome Coronavirus” or “Severe Acute Respiratory Syndrome Coronavirus 2” or “Severe Acute Respiratory Syndrome Corona Virus 2” or SARS-Cov-2 or “Corona Virus” or Coronavirus or “Corona Virinae 19” or “Corona Virinae 2019” or “Corona Virus 19” or “Corona Virus 2019” or ncov19 or ncov2019):ti,ab,kw

Results: 8582

#2: ("clinical features" or "clinical characteristics" or "epidemiological characteristics" or "clinical character" or comorbidities or complications or mortality or death or "underlying diseases" or "related diseases" or "basic diseases" or "clinical outcomes"):ti,ab,kw

Results: 301024

#3: (China or Chinese):ti,ab,kw

Results: 40175

#4: = #1 AND #2 AND #3

Results (*Cochrane Library*): 279

***Web of Science：***

#1: “COVID-19” or “COVID 19” or “COVID 2019” or “Novel Coronavirus” or “Novel Coronavirus 2019” or “Corona Virus Disease 2019” or “Coronavirus Disease” or “Severe Acute Respiratory Syndrome Coronavirus” or “Severe Acute Respiratory Syndrome Coronavirus 2” or “Severe Acute Respiratory Syndrome Corona Virus 2” or “SARS-Cov-2” or “Corona Virus” or “Coronavirus” or “Corona Virinae 19” or “Corona Virinae 2019” or “Corona Virus 19” or “Corona Virus 2019” or ncov19 or ncov2019 or “2019- ncov”

Results: 247068

#2: "clinical features" or "clinical characteristics" or "epidemiological characteristics" or "clinical character" or comorbidities or complications or mortality or death or "underlying diseases" or "related diseases" or "basic diseases" or "clinical outcomes"

Results: 3079516

#3: China or Chinese

Results: 1115247

#4: = #1 AND #2 AND #3

Results (*Web of Science*): 5309

**Four main Chinese-language databases**

***China National Knowledge Infrastructure:***

#1: "新冠" + "新冠病毒肺炎" + “COVID-19” + “COVID 19” + “COVID 2019” + “新型冠状病毒” + “2019年新型冠状病毒” + “2019年冠状病毒” + “冠状病毒” + “严重急性呼吸系统综合症冠状病毒” + “SARS-Cov-2”

Results: 74035

#2: “流行病学” + “临床特征” + “流行病学特征” + 合并症 + 并发症 + 死亡率 + 死亡 + “基础疾病” + “相关疾病” + “基础疾病” + “临床结果”

Results: 2365814

#3: = #1 AND #2

Results: 4016

***Wanfang Data Knowledge Service Platform:***

#1: “新冠” or “新冠肺炎” or “COVID-19” or “COVID 19” or “COVID 2019” or “新型冠状病毒” or “2019年新型冠状病毒” or “2019年冠状病毒病” or “冠状病毒” or “严重急性呼吸系统综合症冠状病毒” or “SARS-Cov-2”

Results: 212934

#2: “流行病学” or “临床特征”or “流行病学特征” or 合并症 or 并发症 or 死亡率 or 死亡 or “基础疾病” or “相关疾病” or “基础疾病” or “临床结果”

Results: 2491137

#3: = #1 AND #2

Results: 5507

***VIP information resource integration service platform databases:***

#1: “新冠” or “新冠肺炎” or “COVID-19” or “COVID 19” or “COVID 2019” or “新型冠状病毒” or “2019年新型冠状病毒” or “2019年冠状病毒病” or “冠状病毒” or “严重急性呼吸系统综合症冠状病毒” or “SARS-Cov-2”

Results: 54194

#2: “流行病学” or “临床特征”or “流行病学特征” or 合并症 or 并发症 or 死亡率 or 死亡 or “基础疾病” or “相关疾病” or “基础疾病” or “临床结果”

Results: 2005145

#3: = #1 AND #2

Results: 3176

***Chinese biomedical literature service system (SinoMed):***

#1: ( "新冠"[常用字段:智能] OR "新冠肺炎"[常用字段:智能] OR "COVID-19"[常用字段:智能] OR "COVID 19"[常用字段:智能] OR "COVID 2019"[常用字段:智能] OR "新型冠状病毒"[常用字段:智能] OR "2019年新型冠状病毒"[常用字段:智能] OR "2019年冠状病毒病"[常用字段:智能] OR "冠状病毒"[常用字段:智能] OR "严重急性呼吸系统综合症冠状病毒"[常用字段:智能] OR "SARS-Cov-2"[常用字段:智能])

Results: 29928

#2: ( "流行病学"[常用字段:智能] OR "临床特征OR 合并症"[常用字段:智能] OR "并发症"[常用字段:智能] OR "死亡率"[常用字段:智能] OR "死亡"[常用字段:智能] OR "基础疾病"[常用字段:智能] OR "相关疾病"[常用字段:智能] OR "基础疾病"[常用字段:智能] OR "临床结果"[常用字段:智能])

Results: 2118763

#3: = #1 AND #2

Results: 6316
